# Anti-inflammatory effects of progesterone through NF-κB and MAPK pathway in lipopolysaccharide- or *Escherichia coli*-stimulated bovine endometrial stromal cells

**DOI:** 10.1371/journal.pone.0266144

**Published:** 2022-04-27

**Authors:** Luying Cui, Xinyu Shao, Wenye Sun, Fangling Zheng, Junsheng Dong, Jun Li, Heng Wang, Jianji Li

**Affiliations:** 1 College of Veterinary Medicine, Yangzhou University, Yangzhou, Jiangsu, PR China; 2 Jiangsu Co-innovation Center for Prevention and Control of Important Animal Infectious Disease and Zoonoses, Yangzhou University, Yangzhou, Jiangsu, PR China; 3 Joint International Research Laboratory of Agriculture and Agriproduct Safety of the Ministry of Education, Yangzhou, Jiangsu, PR China; Chang Gung University, TAIWAN

## Abstract

Postpartum uterine infection in dairy cows is commonly caused by pathogenic bacteria such as *Escherichia coli* (*E*. *coli*). Progesterone elicits immunosuppressive function within bovine endometrium, and has been suggested to be related to postpartum uterine infection. Endometrial stroma is exposed to bacteria due to the disruption of epithelium during parturition, but the effect and mechanism of progesterone on innate immune response of stromal cells has not been reported. This study evaluated the impact of progesterone on inflammatory response of primary endometrial stromal cells stimulated by lipopolysaccharide or heat-killed *E*. *coli*. Quantitative PCR analysis revealed that progesterone repressed mRNA induction of *IL1B*, *IL6*, *TNF*, *CXCL8*, *NOS2*, and *PTGS2* in stromal cells in response to lipopolysaccharide or *E*. *coli* challenge. Consistently, Western blot and immunofluorescence staining results showed that progesterone suppressed lipopolysaccharide- or *E*. *coli*-induced MAPK and NF-κB activations characterized with decreased phosphorylations of ERK1/2, JNK, P38, IκBα, and P65, and inhibition of P65 nuclear translocation. In unstimulated stromal cells, progesterone alone did not affect the mRNA transcription for *IL6*, *TNF*, *CXCL8*, *NOS2*, and *PTGS2*, and the signaling cascade of MAPK and NF-κB, but decreased *IL1B* mRNA expression. These results revealed that the anti-inflammatory effect of progesterone in lipopolysaccharide- or *E*. *coli*-challenged endometrial stromal cells was probably mediated through MAPK and NF-κB pathways.

## Introduction

After parturition, the bovine uterine lumen becomes contaminated, and persistence of pathogenic bacteria often leads to uterine infection, such as metritis and endometritis. These uterine impairments are associated with poor reproductive performance even after successful resolution of the disease, and eventually cause huge financial losses [1]. Among various bacteria that contaminate bovine uterus, *Escherichia coli* (*E*. *coli*) are abundant and are the first step in the pathogenesis of postpartum uterine disease in cattle [2].

Classical innate immunity is a principal component of uterine defense and encompasses anatomical, physiological, phagocytic, and inflammatory barriers [1]. The endometrial epithelial barrier is disrupted due to parturition, making the underlying stroma exposed to pathogenic microorganisms. Lipopolysaccharide (LPS) is the main endotoxin of *E*. *coli*. Exposure of bovine endometrial cells with LPS or *E*. *coli* results in inflammatory response through pattern recognition receptors [2]. Toll-like receptor 4 (TLR4) binds to LPS together with myeloid differentiation factor 2 and CD14 on endometrial cell surface, followed by the activation of nuclear factor-κB (NF-κB) transcription factors and the mitogen-activated protein kinases (MAPK) extracellular signal, including extracellular regulated kinase (ERK), p38, and c-Jun N-terminal kinase (JNK), and finally results in an enhanced expression of transcripts for cytokines and chemokines such as interleukin 1 beta (IL1β), IL6, tumor necrosis factor-α (TNF-α), IL8, nitric oxide, and prostaglandins [2–4].

Progesterone has been proved to show immunosuppressive function within the endometrium of ruminants, and the pathogenesis of postpartum uterine infection can be related to progesterone [5]. Normally after parturition, progesterone concentration keeps low or undetectable until after the first ovulation [6–8]. But an early ovulation could make the cow at a higher risk of uterine infection if uterine involution is incomplete [9]. Spontaneous uterine infection in cattle do not usually develop until after the first postpartum corpus luteum forms and begins producing progesterone [10]. In practice, we found the presence of corpus luteum in about 25% of cows diagnosed with endometritis.

Mechanism underlying the immunosuppressive role of progesterone on uterus of ruminant species was associated with decreased of lymphocyte proliferation, modulation on amount and function of polymorphonuclear leukocyte, and regulation of inflammatory cytokines and chemokines [10–13]. Generally, progesterone exhibited an anti-inflammatory property in various cell types [14–16], and has been shown to perturbate prostaglandin secretions in bovine endometrial epithelial and stromal cells in response to LPS or *E*. *coli* challenge [3]. But the study by Saut et al. found no impact of progesterone on the inflammatory response of bovine endometrial epithelial and stromal cells stimulated by LPS [4]. Previously we verified that progesterone inhibited LPS- or *E*. *coli*-induced expression of proinflammatory genes and MAPK and NF-κB pathways in primary bovine endometrial epithelial cells [17]. Compared with epithelial cells, stromal cells are more susceptible to pathogenic *E*. *coli* with a stronger binding affinity and a more intense inflammatory response [18]. However, the effect of progesterone on bovine endometrial stromal cells (BESC) remains controversial.

In the present study, we hypothesized that progesterone ameliorates inflammatory response in bovine endometrial stromal cells. Primary BESC was stimulated with LPS or heat-killed *E*. *coli*, and was treated with physiological concentrations of progesterone (1, 3, or 5 ng/mL). The changes in mRNA expressions of *IL1B*, *IL6*, *TNF*, C-X-C motif chemokine ligand 8 (*CXCL8*), nitric oxide synthase 2 (*NOS2*), and prostaglandin-endoperoxide synthase 2 (*PTGS2*), and activations of NF-κB and MAPK pathways were determined.

## Materials and methods

### Cell culture

All experimental procedures were approved by the Animal Care and Use Committee of Yangzhou University (NSFC2020-SYXY24). The bovine uteri without gross evidence of genital disease or infection were collected aseptically at local abattoir and were kept on ice until further processing in the laboratory. The uterine surface was disinfected with iodophor and 75% alcohol and was flushed clean with sterile saline. The uterine horn was dissected longitudinally to expose the endometrium. The epithelium was removed by using a moisturized cotton ball. Then the intercaruncular endometrial stripes were dissected from the myometrial layer with a scalpel blade and were chopped into small pieces. After repeated rinses with phosphate-buffered saline (PBS) supplemented with 100 U/mL penicillin/streptomycin, the minced tissue was digested in DMEM/F12 (D8900, sigma, USA) containing 0.25% collagenase II (C6885-5G, Sigma, USA) at 37°C for 50 min. The cell suspension was filtered through a 450 μm mesh to remove undigested tissue fragments, and the filtrate was washed 3 times by centrifugation (100× *g* for 5 min) with PBS. The cells were resuspended in DMEM/F12 containing 15% fetal bovine serum and 100 U/mL penicillin/streptomycin and were cultured at 37°C with 5% CO_2_. The medium was changed 12 h after plating to allow attachment of stromal cells and removal of epithelial cells. The purity of stromal cell population was determined to be more than 95% by the detection of vimentin using immunocytochemistry. The culture media were changed every 24 to 48 h once confluence had been reached approximately 90%.

### Experiment design and treatments

Both LPS and heat-killed *E*. *coli* were used to induce the inflammatory response in BESC. LPS (L2630) and progesterone (P0130) were purchased from Sigma-Aldrich. LPS was dissolved in DMEM/F12 at a concentration of 1 mg/mL as a stock solution at -20°C, and was diluted to 1 μg/mL with DMEM/F12 during experiment. Progesterone was dissolved and diluted in ethanol to 10^4^ ng/mL as a stock solution at -20°C, and was diluted to 1, 3, or 5 ng/mL with DMEM/F12 as working solutions [17]. The heat-killed *E*. *coli* O55:B5 was prepared as previously described [19]. The inactive bacteria were resuspended in DMEM/F12 to a final inoculum of 1×10^8^ CFU/mL. To determine the effect of progesterone on the inflammatory response of BESC, the cells were plated in 6-, 24-, or 96-well plates or 6-cm dishes and were challenged with 1 μg/mL LPS or 1×10^8^ CFU/mL heat-killed *E*. *coli* in control medium or medium containing progesterone. One, 3, and 5 ng/mL progesterone were selected because the serum concentration of progesterone normally ranges from 1 to 5 ng/mL in cattle [4]. Based on our previous reports in epithelial cells and a pilot study in BESC, LPS of 1 μg/mL or heat-killed *E*. *coli* of 1×10^8^ CFU/mL was able to induce the inflammatory response [19]. The cell viability was determined 24 h after treatment. The relative mRNA abundance of inflammatory genes was detected 12 and 24 h after the cotreatment of LPS and progesterone, or 18 and 24 h after the cotreatment of heat-killed *E*. *coli* and progesterone. The changes in key protein levels of NF-κB and MAPK pathways were detected at 45 and 60 min, respectively, in cells with cotreated with LPS and progesterone. The time point for the detection of NF-κB and MAPK pathways was 120 min in cells cotreated with heat-killed *E*. *coli* and progesterone. P65 nuclear translocation was detected using immunofluorescence at 45 or 120 min in cells stimulated with LPS or bacteria, respectively. In addition, to observe the impact of progesterone in unstimulated conditions, BESC was treated with progesterone alone for 12 h to detect proinflammatory gene expressions, and for 30 min to detect phosphorylation of key proteins in MAPK and NF-κB pathways. The selection of these time points was based on pre-experimental results.

### Cell viability assay

The Cell Counting Kit-8 (CCK-8, Dojindo Molecular Technologies, Inc., Kumamoto, Japan) was used to evaluate the impact of progesterone, LPS, and heat-killed *E*. *coli*, either alone or in combination, on the cell viability of BESC. The cells were seeded into wells of a 96-well plate (2×10^3^ cells per well) and grown to 80% confluence. The medium was replaced with DMEM/F12 containing progesterone, LPS and progesterone, or heat-killed *E*. *coli* and progesterone. After 24 h treatment, the CCK-8 solution was added to each well, followed by an additional incubation for 2 h. The optical density was read at 450 nm using a microplate reader (Tecan, Austria).

### RNA extraction and quantitative PCR

The cells were plated in 6-well plates (2×10^5^ cells per well) and grown to 80% fusion. After the treatment as previously described, the cells were washed with PBS, and the total RNA was subsequently extracted using a Trizol reagent (ET111, TRAN, China) according to the manufacturer’s protocol. The extracted RNA was quantified using a Nanodrop 2000 spectrophotometer (Thermo, USA). The absorption ratio (A260/280) was determined to be between 1.8 and 2.0. The RNA was reverse transcribed into cDNA using the PrimerScript RT regent Kit gDNA Eraser (DRR047A, TaKaRa, Japan). The quantitative PCR was carried out using a CFX 96 Real-Time PCR Detection System (Bio-Rad, USA) as previously described [20]. The 2^-△△Ct^ method was used to analyze the relative mRNA abundance. The actin beta (*ACTB*) was used as an internal control. A single product was amplified by each primer pair. The products were purified and sequenced (TsingKe Biotech, Beijing, China), and the sequence results were analyzed using BLAST (http://blast.ncbi.nlm.nih.gov/blast.cg) and compared to GenBank database. The sequences of primers were shown in Table 1.

**Table 1:** Primer sequences used for quantitative PCR.

Gene	Primer sequence (5′ → 3′)	Length (bp)	NCBI accession
*ACTB *	F: CATCACCATCGGCAATGAGC	156	NM_173979.3
	R: AGCACCGTGTTGGCGTAGAG		
*IL1B*	F: TGATGACCCTAAACAGATGAAGAGC	134	NM_174093.1
	R: CCACGATGACCGACACCACCT		
*IL6*	F: TGAAAGCAGCAAGGAGACACT	90	NM_173923.2
	R: TGATTGAACCCAGATTGGAAGC		
*TNF*	F: GGGCTTTACCTCATCTACTCACAG	132	NM_173966.3
	R: GATGGCAGACAGGATGTTGACC		
*CXCL8*	F: TTCCTCAGTAAAGATGCCAATG	86	NM_173925.2
	R: TGACAACCCTACACCAGACCCA		
*NOS2*	F: GAGTGACTTTCCAAGACACGC	186	NM_001076799.1
	R: TGAAGGAGCCGTAATACTGGT		
*PTGS2*	F: CCAGAGCTCTTCCTCCTGTG	213	NM_174445.2
	R: AAGCTGGTCCTCGTTCAAAA		

### Western blot analysis

The cells were seeded in 6-cm culture dishes (6×10^5^ cells per dish) and grown to 80% confluence. After treatment, the cells were collected and lysed using a RIPA buffer (P0013B, Beyotime, China) containing protease and phosphatase inhibitor cocktail (P1046, Beyotime, China) for routine protein extraction. The total proteins were quantified using a bicinchoninic acid protein assay kit (P0010, Beyotime, China). Cell protein extract (20 to 30 μg) was subjected to a 10% SDS-polyacrylamide gel and was transferred to a polyvinylidene difluoride membrane (Millipore, Germany). The samples were blocked with Tris-buffered saline containing 0.05% Tween 20 and 5% skimmed milk, followed by the incubation with a primary antibody (1:1000 dilution with 5% bovine serum albumin) at 4°C overnight. The primary antibodies specific for β-actin (# 4970), IκBα (# 4812), P-IκBα (# 2859), P65 (# 8242), P-P65 (# 3033), ERK1/2 (# 4695), P-ERK 1/2 (# 4370), JNK (# 9258), P-JNK (# 4668), P38 (# 8690), and P-P38 (# 4511) were purchased from Cell Signaling Technology. The membranes were subsequently incubated with an HRP-conjugated secondary antibody (111-035-003, Jackson ImmunoResearch, USA; 1:10000 dilution with 5% skimmed milk) at room temperature for 1 h. The protein blots were detected and visualized using a chemiluminescence HRP substrate (1810202, Clinx Science Instruments, China) and a ChemiScope 5300 Pro CCD camera (Clinx Science Instruments, China). The blots were quantified by Quantity One software (Bio-Rad, CA, USA).

### Immunofluorescence staining

The cells were plated in a 24-well culture plate and were treated according to the experiment design. Then the cells were fixed with 4% paraformaldehyde at room temperature for 12 min. After washing with PBS, the cell membrane was penetrated with 0.4% Triton X-100 (ST797, Beyotime, China) for 15 min. The cells were washed with PBS and were blocked using PBS containing 10% goat serum (ZLI-9021, ZSGB-BIO, China) for 45 min at room temperature. The cells were incubated with a primary antibody specific for NF-κB P65 (#8242, Cell Signaling Technology, USA) with 1:400 dilution at 4°C overnight, and were subsequently incubated with an FITC-conjugated secondary antibody (A11034, Thermo Fisher Scientific, USA) with 1:500 dilution for 1 h at room temperature in dark environment. The nuclei were stained with DAPI (C1005, Beyotime, China). The cells were visualized using a fluorescence microscope (Leica TCS Sp8, Leica company, Germany).

### Statistical analysis

All data were presented as the means ± standard error of means (SEM), and were analyzed using the SPSS 26.0 software (IBM, NY, USA). Statistically significant differences were calculated by one-way ANOVA, followed by Least Significant Difference test. A two-sided *P*-value less than 0.05 was designated as significant. Each experiment was repeated at least three times.

## Results

### BESC viability

**Fig 1:**
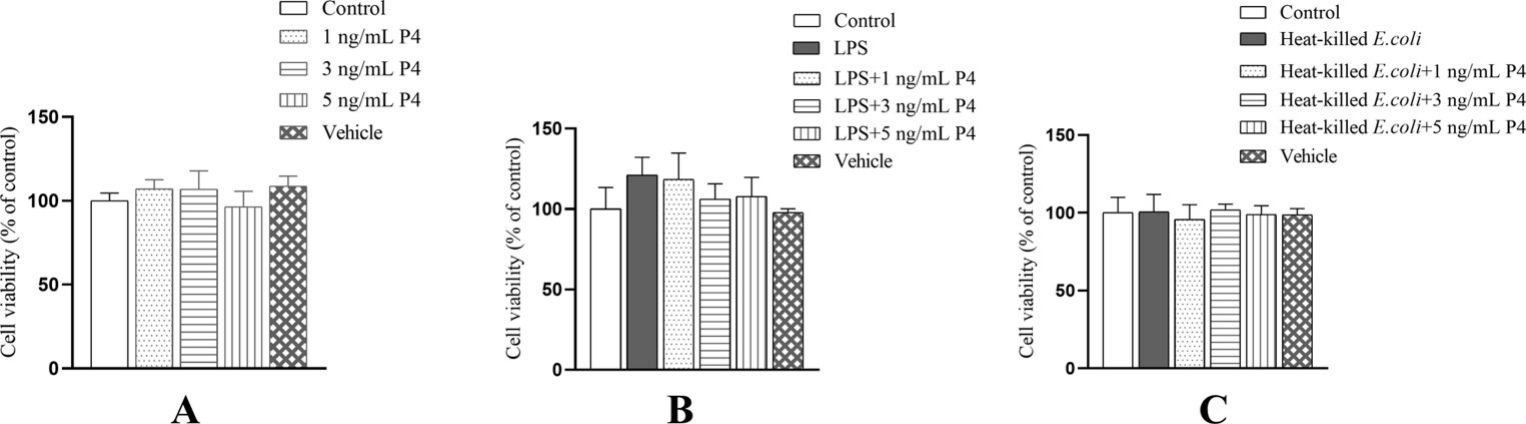
The changes in cell viability of primary bovine endometrial stromal cells using Cell Counting Kit-8 method. The cells were treated with 1, 3, or 5 ng/mL progesterone (A), 1 μg/mL lipopolysaccharide and progesterone (B), or 1×10^8^ CFU/mL heat-killed *E*. *coli* and progesterone (C) for 24 h. The vehicle is 0.5% alcohol. P4, progesterone. LPS, lipopolysaccharide. All data were presented as means ± SEM (n ≥ 3).

The BESC viability was not influenced (*P* > 0.05) by the treatment of progesterone, LPS, or heat-killed *E*. *coli* individually or in combination (Fig 1).

### Proinflammatory gene expression

**Fig 2:**
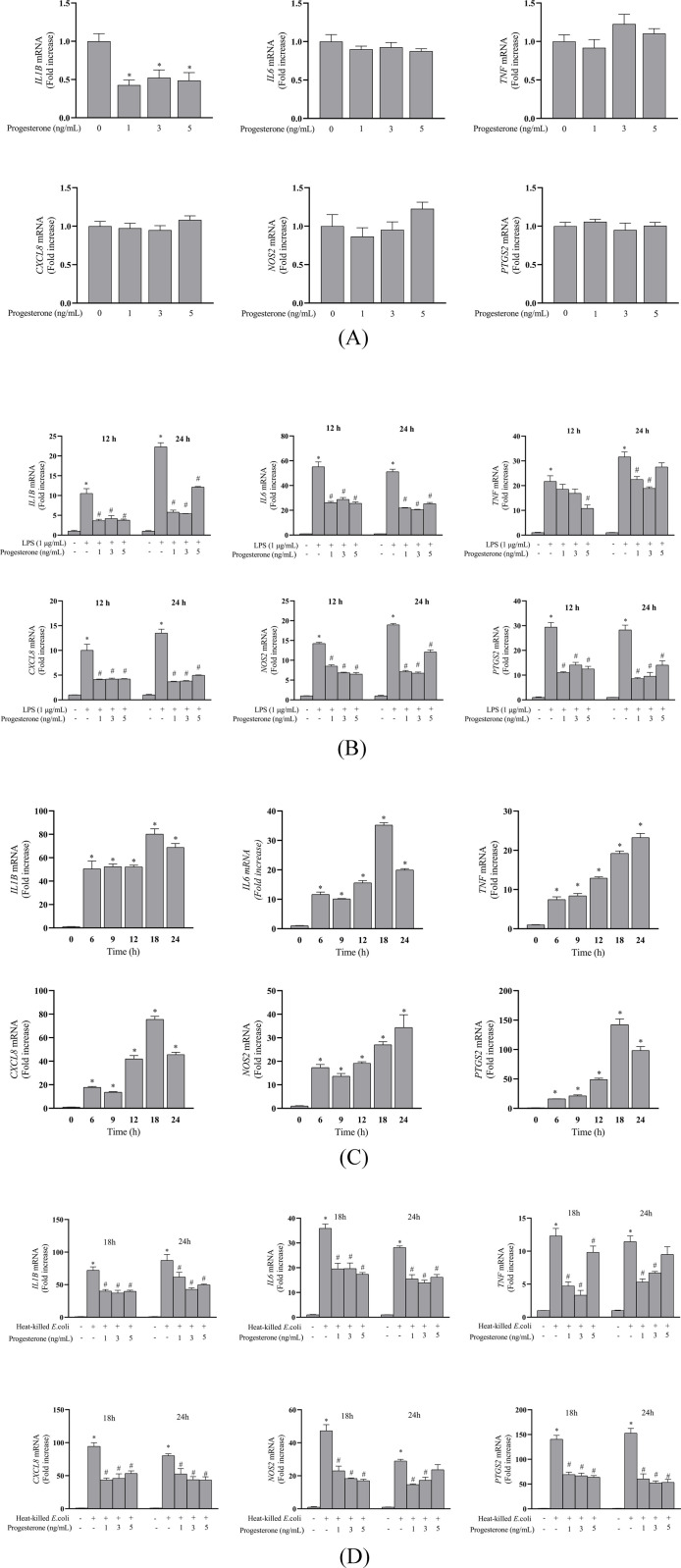
Progesterone treatment attenuated gene expression of inflammatory mediators in primary bovine endometrial stromal cells stimulated with lipopolysaccharide or heat-killed *E*. *coli*. The mRNA expressions of *IL1B*, *IL6*, *CXCL8*, *TNF*, *NOS2*, and *PTGS2* were assessed by qPCR (normalized to *ACTB*). A. Unstimulated cells were treated with 1, 3, and 5 ng/mL progesterone for 12 h. B. Cells were cotreated with 1 μg/mL lipopolysaccharide and progesterone (1, 3, and 5 ng/mL) for 12 and 24 h. C. Time-course changes in inflammatory gene expressions in cells treated with 1×10^8^ CFU/mL heat-killed *E*. *coli* for 6, 9, 12, 18, and 24 h. D. Cells were cotreated with 1×10^8^ CFU/mL heat-killed *E*. *coli* and progesterone (1, 3, and 5 ng/mL) for 18 and 24 h. LPS, lipopolysaccharide. All data were presented as means ± SEM (n ≥ 3). * *P* < 0.05, difference compared with the control; # *P* < 0.05, difference compared with the LPS group.

The impact of progesterone alone on proinflammatory gene expressions was evaluated at 12 h. This time point was selected according to a pre-experimental result (unpublished data, S1 Appendix). The mRNA expressions of *IL6*, *TNF*, *CXCL8*, *NOS2*, or *PTGS2* were unaffected (*P* > 0.05) by 1, 3, or 5 ng/mL progesterone. However, progesterone decreased (*P* < 0.05) *IL1B* mRNA expression (Fig 2A). Based on a pilot study, time points of 12 and 24 h exhibited peak abundance of mRNA transcripts for proinflammatory genes in LPS-treated BESC (unpublished data, S1 Appendix). As shown in Fig 2B, exposure of cells to LPS upregulated (*P* < 0.05) relative mRNA abundance of *IL1B*, *IL6*, *TNF*, *CXCL8*, *NOS2*, and *PTGS2*. Compared with LPS group, progesterone (1, 3, and 5 ng/mL) decreased (*P* < 0.05) mRNA expressions of *IL1B*, *IL6*, *CXCL8*, *NOS2*, and *PTGS2* in response to LPS at 12 and 24 h. Down-regulation (*P* < 0.05) of *TNF* mRNA expression was only observed in cells cotreated with LPS and 5 ng/mL progesterone at 12 h, and in cells cotreated with LPS and progesterone (1 or 3 ng/mL) at 24 h. The time course experiment showed that heat-killed *E*. *coli* induced expression of proinflammatory genes (*P* < 0.05), and the increase was most prominent at 18 and 24 h (Fig 2C). Progesterone (1, 3, and 5 ng/mL) generally reduced (*P* < 0.05) *IL1B*, *IL6*, *TNF*, *CXCL8*, *NOS2*, and *PTGS2* mRNA expression at 18 and 24 h in BESC with bacterial challenge, except (*P* > 0.05) *TNF* and *NOS2* at 24 h in cells cotreated with *E*. *coli* and 5 ng/mL P4 (Fig 2D).

### NF-κB activation

**Fig 3:**
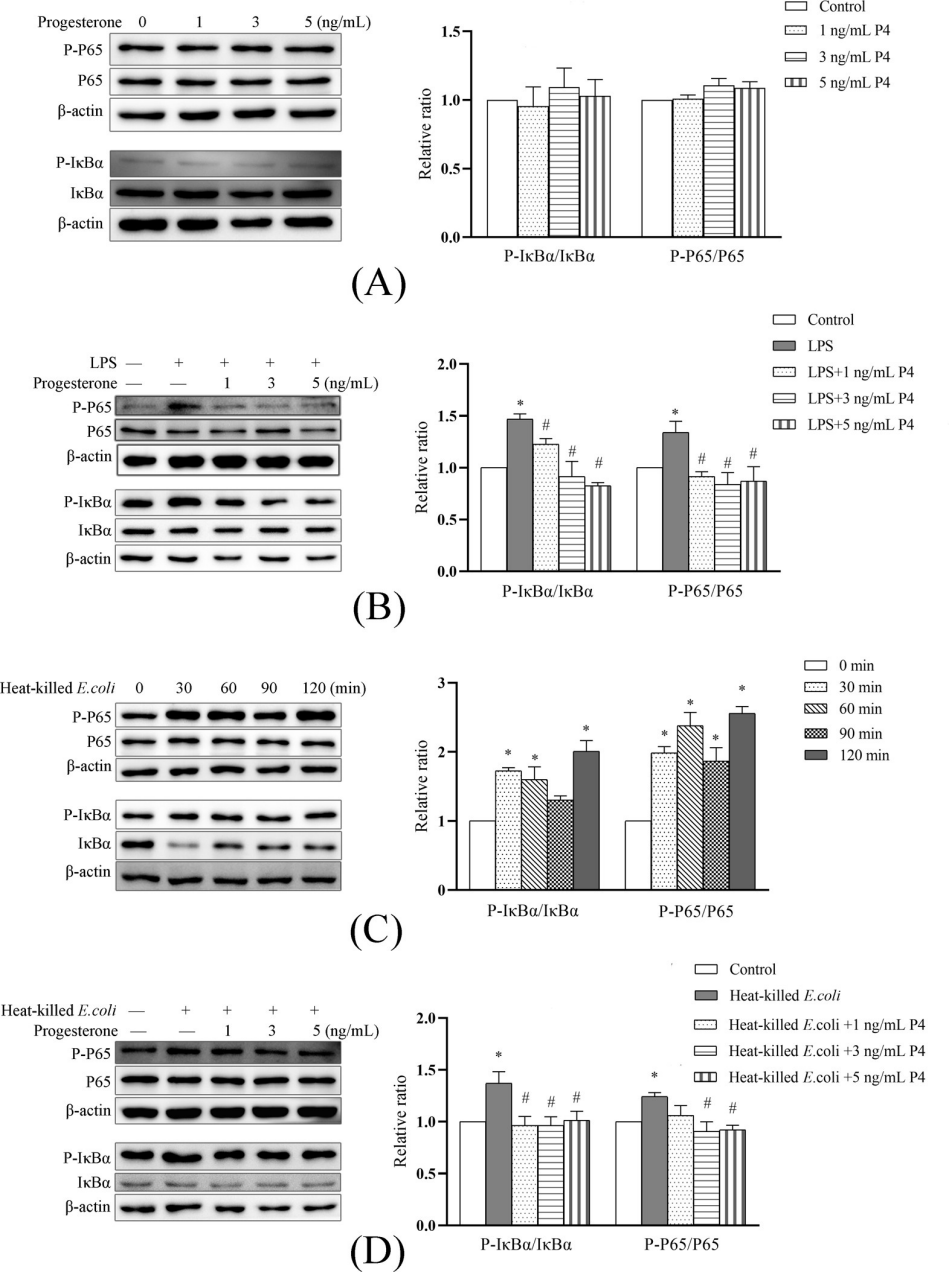
Progesterone inhibited phosphorylations of IκBα and P65 in primary bovine endometrial stromal cells in response to lipopolysaccharide or heat-killed *E*. *coli* stimulation. The protein level of IκBα, P-IκBα, P65, and P-P65 was determined using Western blot. The protein blots were quantified by Quantity One software. A. Unstimulated cells were treated with 1, 3, and 5 ng/mL progesterone for 30 min. B. The cells were cotreated with 1 μg/mL lipopolysaccharide and progesterone (1, 3, or 5 ng/mL) for 45 min. This time point was based on the results of our previous study. C. Time-course changes in key protein phosphorylation of NF-κB pathway in cells challenged with 1×10^8^ CFU/mL heat-killed *E*. *coli* for 30, 60, 90, and 120 min. D. The cells were cotreated with heat-killed *E*. *coli* and progesterone for 120 min. LPS, lipopolysaccharide. P4, progesterone. All data were presented as means ± SEM (n ≥ 3). * *P* < 0.05, difference compared with the control; # *P* < 0.05, difference compared with the LPS group (B) or the heat-killed *E*. *coli* group (D).

As shown in Fig 3A, progesterone (1, 3, or 5 ng/mL) alone did not influence (*P* > 0.05) key protein levels in NF-κB pathway. Our preliminary study showed that the phosphorylations of IκBα and P65 elevated at 45 min in response to LPS stimulation (unpublished data, S1 Appendix). The addition of progesterone decreased (*P* < 0.05) LPS-induced phosphorylations of P65 and IκBα. The heat-killed *E*. *coli* resulted in phosphorylations of IκBα and P65 from 30 to 120 min, and was maximal (*P* < 0.05) at 120 min (Fig 3B). The amount of phosphorylations of IκBα and P65 in cells cotreated with progesterone (1, 3, or 5 ng/mL) and heat-killed *E*. *coli* was generally less (*P* < 0.05) than in cells treated only with heat-killed *E*. *coli*.

**Fig 4:**
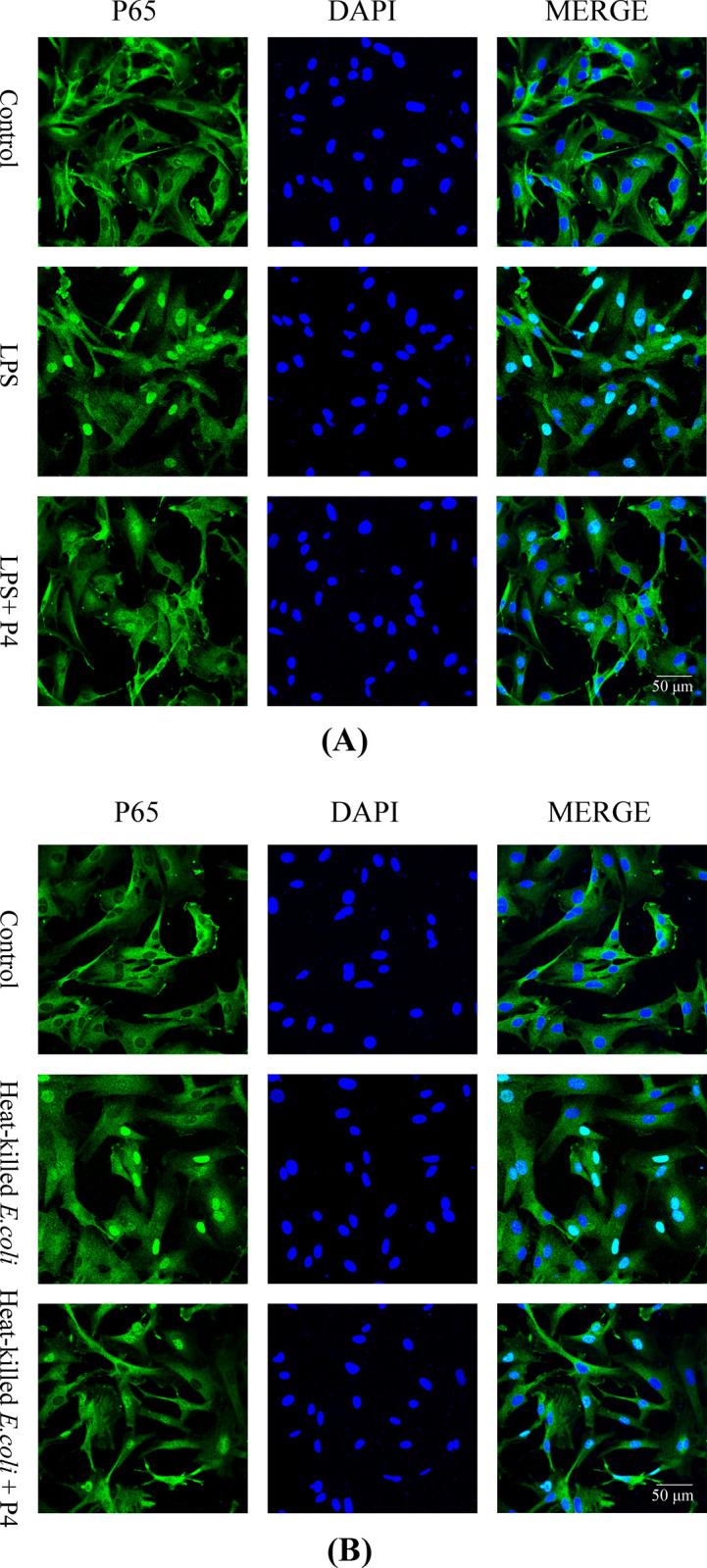
Progesterone inhibited nuclear translocation of P65 in primary bovine endometrial epithelial cells stimulated with lipopolysaccharide or *E*. *coli*. The cells were cotreated with 1 μg/mL lipopolysaccharide and 3 ng/mL progesterone for 45 min (A), or cotreated with 1×10^8^ CFU/mL heat-killed *E*. *coli* and 3 ng/mL progesterone for 120 min (B). LPS, lipopolysaccharide. P4, progesterone.

The immunofluorescence results showed translocation of NF-κB P65 into the nucleus in BESC stimulated with LPS and heat-killed *E*. *coli* (Fig 4). The addition of 3 ng/mL progesterone reduced the amount of P65 in nucleus in cells challenged with LPS or bacteria.

### MAPK phosphorylation

**Fig 5:**
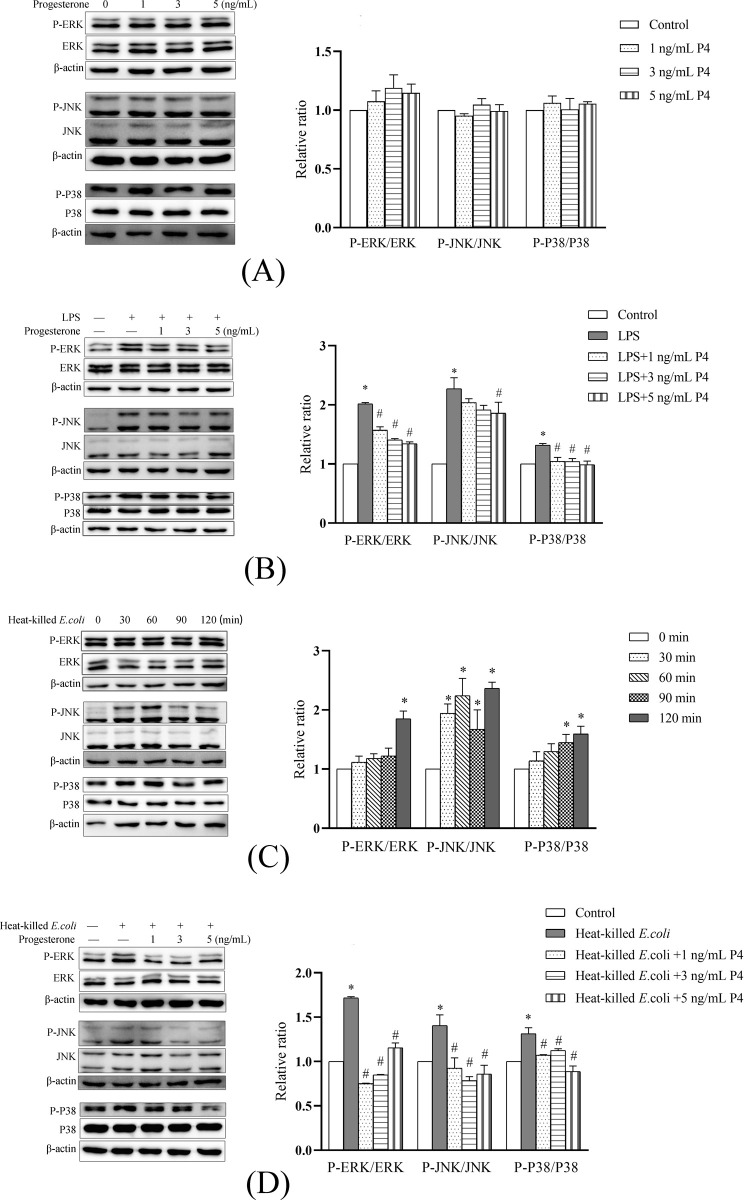
Progesterone suppressed phosphorylations of ERK, JNK, and P38 in primary bovine endometrial stromal cells in response to lipopolysaccharide or heat-killed *E*. *coli* stimulation. The protein levels of ERK, P-ERK, JNK, P-JNK, P38, and P-P38 was determined using Western blot. The blots were quantified by Quantity One software. A. Unstimulated cells were treated with 1, 3, and 5 ng/mL progesterone for 30 min. B. The cells were cotreated with 1 μg/mL lipopolysaccharide and progesterone (1, 3, or 5 ng/mL) for 60 min. The time point was based on the results of our previous study. C. Time-course changes in key protein phosphorylation of MAPK pathway in cells challenged with 1×10^8^ CFU/mL heat-killed *E*. *coli* for 30, 60, 90, and 120 min. D. The cells were cotreated with heat-killed *E*. *coli* and progesterone for 120 min. LPS, lipopolysaccharide. P4, progesterone. All data were presented as means ± SEM (n ≥ 3). * *P* < 0.05, difference compared with the control; # *P* < 0.05, difference compared with the LPS group (B) or the heat-killed *E*. *coli* group (D).

The cells treated with only progesterone (1, 3, and 5 ng/mL) showed no change (*P* > 0.05) in key protein levels of MAPK pathway (Fig 5A). Treatment of BESC with 1 μg/mL LPS induced phosphorylations of ERK, JNK, and P38 at 60 min (data unpublished, S1 Appendix). As shown in Fig 5B, compared with LPS group, the ratios of P-ERK/ERK, P-JNK/JNK, and P-P38/P38 were generally lower in cells of cotreatment groups, except the P-JNK/JNK ratio in cells cotreated with LPS and progesterone (1 or 3 ng/mL). Heat-killed *E*. *coli* caused elevation (*P* < 0.05) in the phosphorylations of ERK, JNK, and P38, with peak levels at 120 min (Fig 5C). Similarly, progesterone (1, 3, and 5 ng/mL) treatment reduced (*P* < 0.05) the phosphorylations of ERK, JNK, and P38 in response to bacterial challenge (Fig 5D).

## Discussion

This study evaluated the impact of progesterone on inflammatory response of primary bovine endometrial stromal cells. As expected, treatment of BESC with LPS or heat-killed *E*. *coli* resulted in upregulated mRNA expressions of *IL1B*, *IL6*, *TNF*, *CXCL8*, *NOS2*, and *PTGS2*, as well as the phosphorylation of key proteins in MAPK and NF-κB pathways. The addition of progesterone decreased these proinflammatory gene expressions and inhibited activations of MAPK and NF-κB signaling in BESC with inflammatory response. However, in resting state, progesterone did not influence these proinflammatory genes or related pathways except *IL1B* mRNA expression.

Increased expressions of proinflammatory genes in response to LPS or *E*. *coli* in BESC has been reported extensively [2, 21–25]. In agreement with these reports, we observed upregulated mRNA encoding *IL1B*, *IL6*, *TNF*, *CXCL8*, *NOS2*, and *PTGS2*. Proinflammatory cytokines such as IL1β, IL6, and TNF participate in the recruitment of white blood cells to inflamed tissue, the production of acute-phase proteins, the cell death of inflamed tissue, and the modification of vascular endothelial permeability [26]. IL8, encoded by *CXCL8* gene, is a powerful chemo-attractant that recruits neutrophils and lymphocytes [27]. Nitric oxide and prostaglandins are inflammatory mediators processed through inducible nitric oxide synthase and cyclooxygenase, respectively. Cyclooxygenase-2 (COX-2), encoded by *PTGS2* gene, is the primary enzyme involved in the biosynthesis of most prostaglandins in bovine endometrium [28].

Previously we observed inhibited mRNA expressions of *IL1B*, *IL6*, *TNF*, and *CXCL8* by progesterone in primary bovine endometrial epithelial cells with inflammatory response [17], which was similar to the result of current study that progesterone decreased *IL1B*, *IL6*, *TNF*, *CXCL8*, *NOS2*, and *PTGS2* mRNA expressions induced by LPS or heat-killed *E*. *coli* in BESC. Bovine endometrial epithelial and stromal cells preincubated with progesterone secreted less prostaglandin after LPS treatment [3]. Progesterone (10^−9^ to 10^−7^ M) was found to suppress IL6 and CXCL8 at both mRNA and protein levels in human endometrial epithelial cell line exposed to LPS and high-mobility group box 1 [29]. In the study of human endometriosis, progesterone (10^−6^ mol/L) was found to be effective in attenuating TNFα/estrogen-induced expression of CXCL8 in endometriotic stromal cells [30]. The suppressed expression or production of inflammatory mediators has also been reported in murine macrophages [31, 32], mature rat dendritic cells [33], human intestinal epithelial cells [34], and human umbilical vein endothelial cells [14] by progesterone or through progesterone receptor (PGR). Contrary to the findings mentioned above, Saut et al. observed that pretreatment of BESC with progesterone (1 to 30 ng/mL) did not modulate IL6 or CXCL8 responses to 100 ng/mL LPS [4]. Such discrepancy is probably due to the differences in LPS concentration, treatment time and detection time. It is noteworthy that progesterone of 5 ng/mL seemed to be not as effective as progesterone of 1 and 3 ng/mL in inhibiting expressions of some proinflammatory genes, such as *IL1B*, *TNF*, and *NOS2* at 24 h in LPS- or *E*. *coli*-stimulated BESC. Such result was not observed in epithelial cells with similar treatment [17], probably indicating the specific mechanism of BESC. Progesterone concentration has been found to influence mRNA expressions of both PGRA and PGRB isoforms and thus regulates their effects within bovine endometrial cells [35]. PGRB is a progesterone-dependent gene activator, whereas PGRA is a weak gene activator and a potent PGRB inhibitor, thereby reducing the effects of progesterone [35]. Therefore, the difference between progesterone of 5 ng/mL and progesterone of 1 and 3 ng/mL in regulating *IL1B*, *TNF*, and *NOS2* mRNA expressions in BESC may be related to the changes in the relative levels of PGR isoforms, and requires further study.

Inconsistent results have been shown in literature about the effect of progesterone alone on the production or gene expression of inflammatory mediators *in vitro*. The secretions of IL6, IL8, or prostaglandins were unaffected in bovine endometrial epithelial and stromal cells pretreated with 5 ng/mL progesterone [3, 4]. Mild upregulations of *IL1B*, *IL6*, *TNF*, and *CXCL8* mRNA by progesterone (5 ng/mL) in bovine endometrial epithelial cells have been reported by our lab [17]. Increased production of TNFα has been observed in human endometrial epithelial cells treated with 1 μmol/L progesterone [36]. Unchanged productions of IL1β and TNFα were found by Butts et al. in rat dendritic cells treated with progesterone (10^−10^ and 10^−8^ mol/L) [33]. A negative regulation of progesterone (10^−7^ mol/L) on mRNA expressions of *IL6* and *CXCL8* was demonstrated by Goddard et al. [14]. We observed that progesterone (1, 3, and 5 ng/mL) alone showed no influence on basal *IL6*, *TNF*, *CXCL8*, *NOS2*, and *PTGS2* mRNA transcriptions, except a down-regulation of *IL1B* mRNA transcript. Signaling cascades of MAPK and NF-κB probably don’t mediate progesterone’s inhibition of basal *IL1B* transcription because these pathways were unaffected in unstimulated BESC. Regulation of *IL1B* transcription by progesterone or through PGR was rarely reported in unstimulated cells. Methylation of specific CpG sites in the proximal promoters of *IL1B* gene correlates with its expression in human chondrocytes [37]. Myeloid cells transcribe *IL1B* gene constitutively at a low level with an absolute requirement of Spi1 transcription factor [38]. Further investigation could focus on molecular mechanisms at transcriptional level, such as transcription factors or microRNAs that constitutively regulate *IL1B* gene.

Activation of TLR4 by LPS results in inductions of NF-κB and MAPK signaling cascades, where enhanced cytokine expression is a major response [39]. In unstimulated conditions, NF-κB is sequestered in the cytoplasm by the inhibitor of NF-κB (IκB) family. Upon stimulation, the IκB kinase complex is activated and phosphorylates IκB molecules, targeting them for ubiquitination and subsequent degradation. Degradation of IκB releases NF-κB, which translocates into the nucleus to initiate a transcriptional response [40]. Our result coincided with previous reports that LPS activated NF-κB pathway in BESC [2, 24]. Progesterone has been shown to prevent NF-κB activation and so inhibit proinflammatory mediator production in reproductive tissues and immune cells [13]. Suppression of NF-κB by progesterone has been reported in bovine and human endometrial epithelial cells [17, 29], murine macrophages [31], mouse microglia [15], and human CD4^+^ T cells [41]. In line with previous studies, we found that progesterone suppressed NF-κB activation in BESC stimulated with LPS or heat-killed *E*. *coli*. There was no change in key protein levels of NF-κB pathway in BESC treated with progesterone alone, which was similar to the observations in bovine endometrial epithelial cells [17], murine macrophages [31], and mouse microglia [15].

The well-known MAPK pathway, mainly composed of ERK1/2, JNK, and P38, transduces environmental and developmental signals into adaptive and programmed responses such as survival, proliferation, differentiation, inflammation, and apoptosis [42]. Activation of MAPK has been shown to initiate inflammatory gene expression in LPS-induced bovine endometrial stromal cells [2, 24, 43]. Consistently, we found LPS-induced phosphorylations of ERK1/2, JNK, and P38 in BESC. The involvement of MAPK in anti-inflammatory action of progesterone *in vitro* has been reported. Activation of ERK1/2, JNK, and p38 induced by LPS or *E*. *coli* was abrogated by the addition of progesterone in primary bovine endometrial epithelial cells [17]. Pretreatment of human endometrial stromal cells with 100 ng/mL progesterone for 9 days suppressed activation of p38 MAPK induced by IL1β [44]. Progesterone (10^−8^ to 10^−6^ M) attenuated LPS-mediated p38, JNK, and ERK phosphorylations in mouse microglia, and mifepristone reversed progesterone’s effect [15]. Similar to these observations, we found inhibited activations of ERK1/2, JNK, and p38 by progesterone in BESC in response to LPS or heat-killed *E*. *coli*. In the study of primary human myometrial cells with inflammatory response, Lei et al. reported that progesterone induced MAPK phosphatase-1, which can reverse phosphorylation events of MAPK, and this anti-inflammatory effect of progesterone was mediated by glucocorticoid receptor rather than progesterone receptor [45], suggesting a more complex mechanism of progesterone antagonism on inflammation. Similar to our previous observation in bovine endometrial epithelial cells [17], progesterone alone did not affect MAPK pathway in BESC, indicating that the molecular mechanism of progesterone is different between the cells in resting state and the cells with inflammatory response. Both MAPK and NF-κB can be directly activated by LPS stimulation [46]. These pathways have been found to synergize through synchronized binding to κB and AP-1 sites found together in the promoters of many genes, including *CXCL8* [2]. Furthermore, the production of proinflammatory cytokines, such as IL1β and TNFα, in turn activate MAPK and NF-κB, forming a positive feedback [46]. Due to the complex interactions of these pathways, the exact intracellular signaling mechanism of anti-inflammation by progesterone in bovine endometrial cells deserves further investigation by using pathway inhibitors and gene silencing and overexpression techniques.

## Conclusions

Progesterone elicited anti-inflammatory effect in LPS- or heat-killed *E*. *coli*-stimulated primary bovine endometrial stromal cells by downregulating gene expressions of *IL1B*, *IL6*, *TNF*, *CXCL8*, *NOS2*, and *PTGS2* corresponding to inhibitions of NF-κB and MAPK activation. Except the downregulation of basal *IL1B* transcription, progesterone alone showed no influence on these proinflammatory gene expressions and NF-κB and MAPK signaling cascades in unstimulated BESC.

## Supporting information

S1 Appendix Data unpublished. (DOCX)

S2 Appendix Raw data used for analysis. (XLSX)

S3 Appendix Uncropped original images. (ZIP)
