# Supplementary material for: Anti-inflammatory effects of progesterone through NF-κB and MAPK pathway in lipopolysaccharide- or Escherichia coli-stimulated bovine endometrial stromal cells
Source: PLoS One. 2022 Apr 27;17(4):e0266144. doi: 10.1371/journal.pone.0266144 (PMC9045630; doi:10.1371/journal.pone.0266144)
Supplement: S1 Appendix — (DOCX) [file pone.0266144.s001.docx]

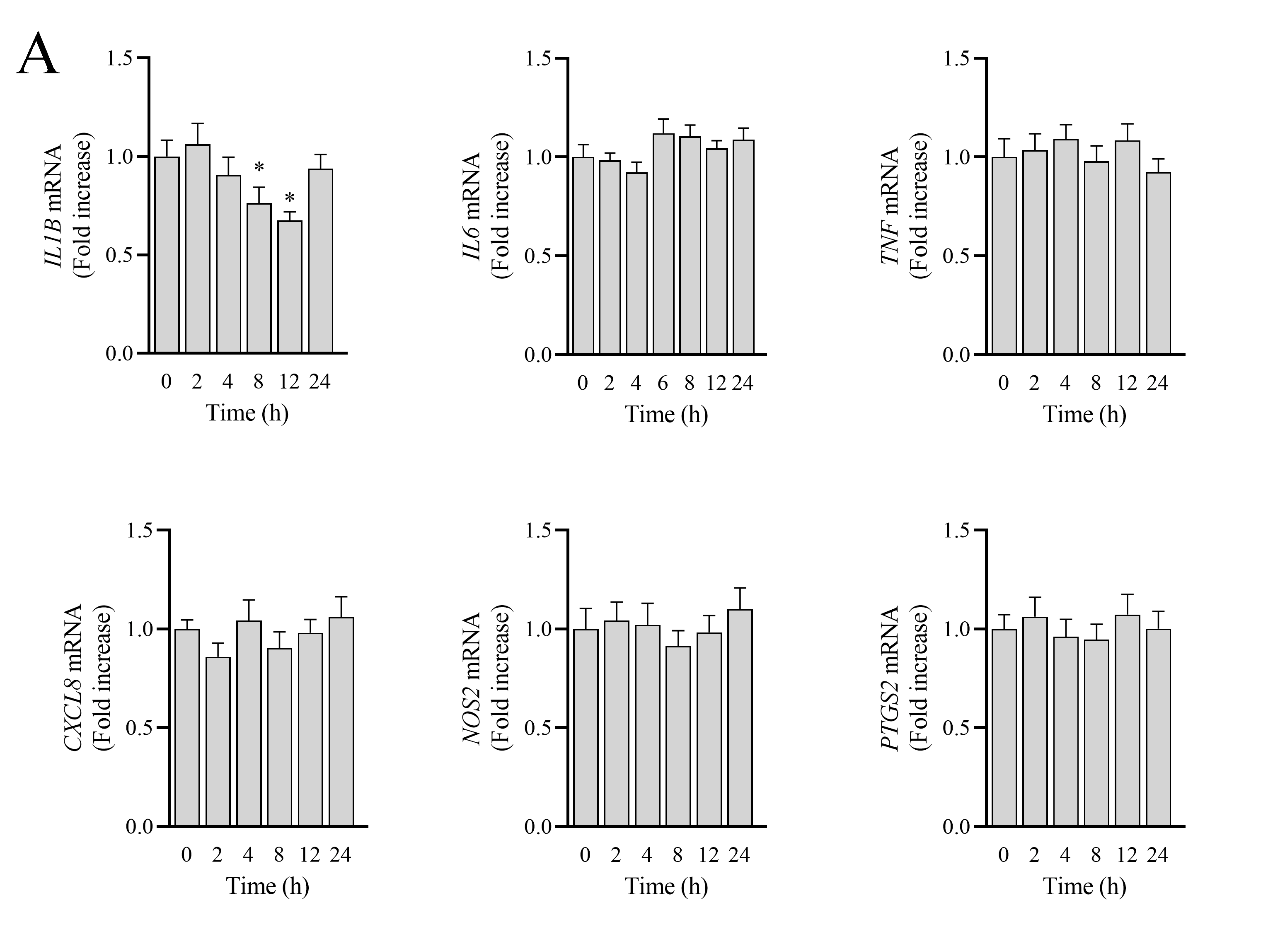


**Supplementary file 1A** Changes in mRNA expressions of *IL1B*, *IL6*, *TNF*, *CXCL8*, *NOS2*, and *PTGS2* in primary bovine endometrial stromal cells treated with 5 ng/mL progesterone. Values were presented as means ± SEM (n = 3). **P*<0.05 vs. the control group.


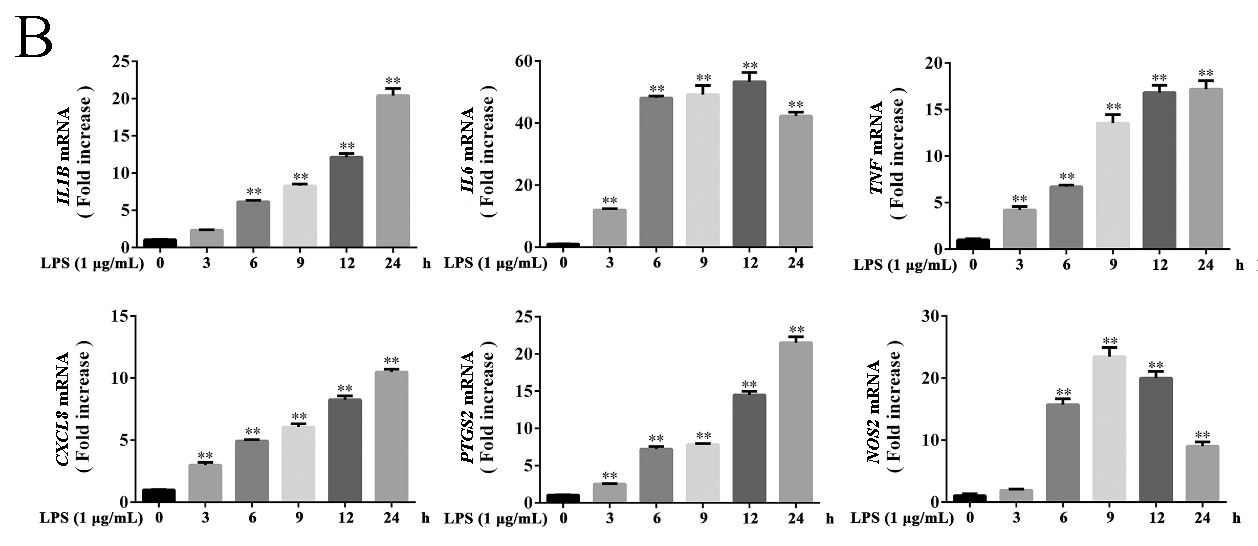


**Supplementary file 1B** Changes in mRNA expressions of *IL1B*, *IL6*, *TNF*, *CXCL8*, *PTGS2*, and *NOS2* in primary bovine endometrial stromal cells stimulated with 1 μg/mL LPS for 0, 3, 6, 9, 12, and 24 h. LPS, lipopolysaccharide. Values are presented as means ± SEM (n = 3). **P*< 0.05, ***P*< 0.01 vs. the control group.


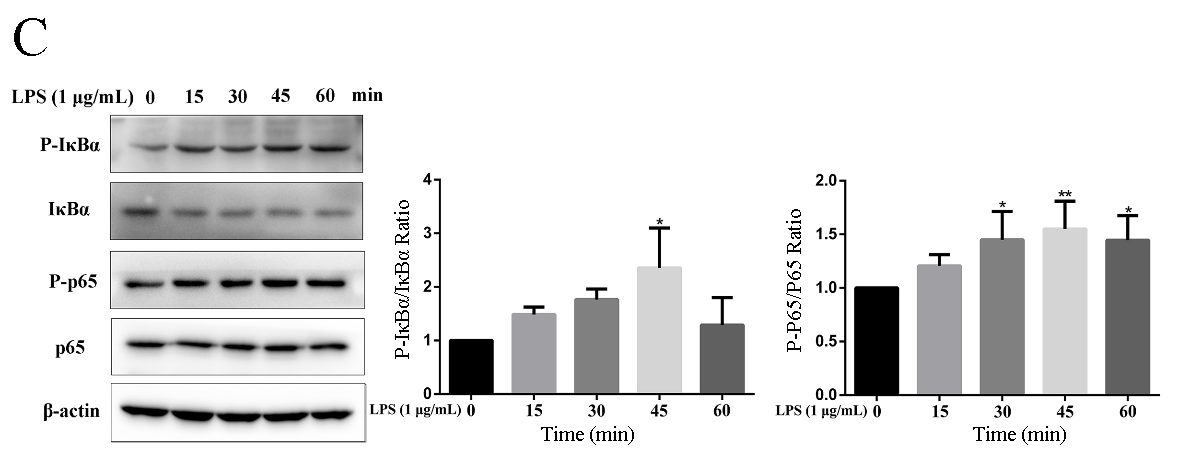


**Supplementary file 1C** Changes in the key protein levels of NF-κB pathway in primary bovine endometrial stromal cells stimulated with 1 μg/mL LPS for 0, 15, 30, 45, and 60 min. The phosphorylation levels of IκBα and P65 were determined by Western blot analysis. LPS, lipopolysaccharide. Values are presented as means ± SEM (n = 3). **P*< 0.05, ***P*< 0.01 vs. the control group.


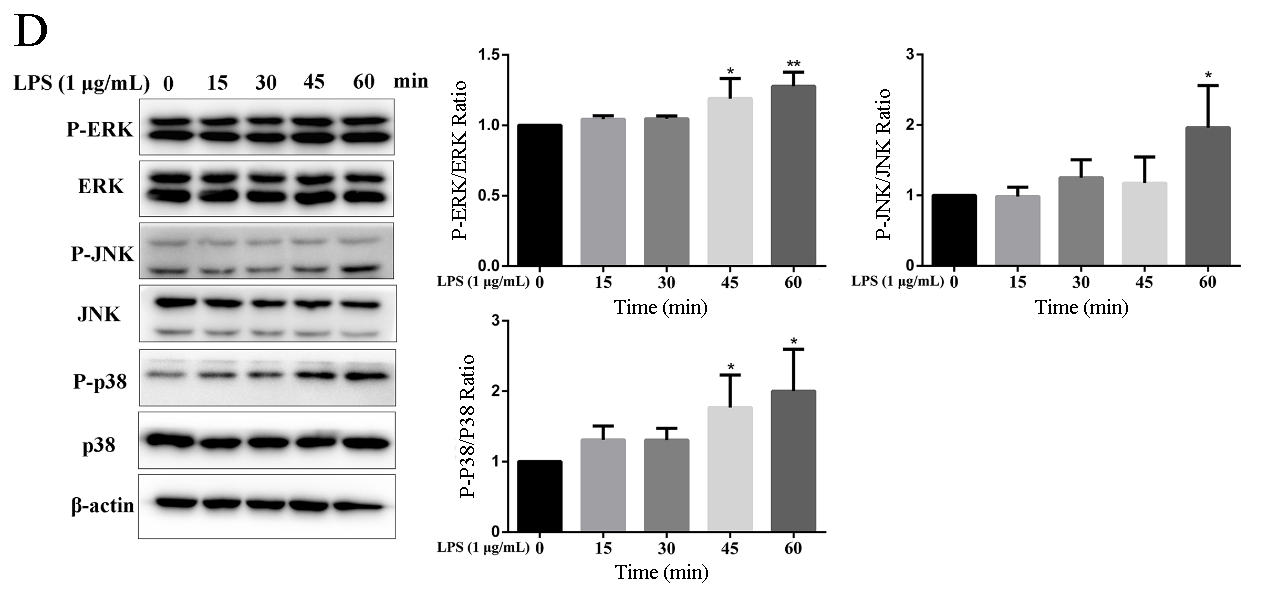


**Supplementary file 1D** Changes in the key protein levels of MAPK pathway in primary bovine endometrial stromal cells stimulated with 1 μg/mL LPS for 0, 15, 30, 45, and 60 min. The phosphorylation of ERK1/2, JNK, and P38 were determined by Western blot analysis. LPS, lipopolysaccharide. Values were presented as means ± SEM (n = 3). **P*< 0.05, ***P*< 0.01 vs. the control group.


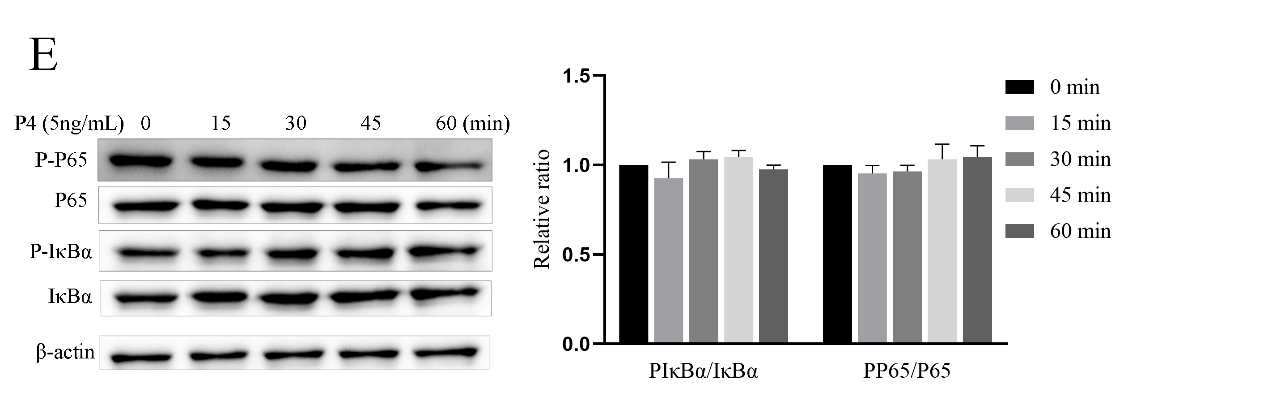


**Supplementary file 1E** Changes in the key protein levels of NF-κB pathway in primary bovine endometrial stromal cells stimulated with 5 ng/mL progesterone for 0, 15, 30, 45, and 60 min. P4, progesterone. The phosphorylation levels of IκBα and P65 were determined by Western blot analysis. Values are presented as means ± SEM (n = 3).


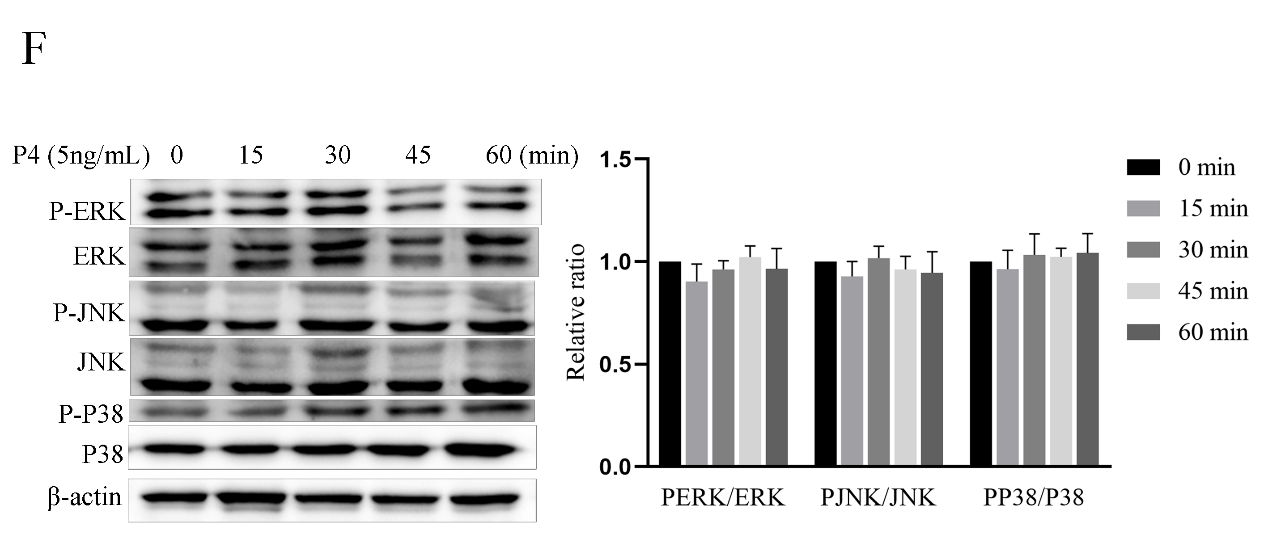


**Supplementary file 1F** Changes in the key protein levels of MAPK pathway in primary bovine endometrial stromal cells stimulated with 5 ng/mL progesterone for 0, 15, 30, 45, and 60 min. The phosphorylation of ERK1/2, JNK, and P38 were determined by Western blot analysis. P4, progesterone. Values were presented as means ± SEM (n = 3).
