# Supplementary figures and images for: Anti-inflammatory effects of progesterone through NF-κB and MAPK pathway in lipopolysaccharide- or Escherichia coli-stimulated bovine endometrial stromal cells
Source: PLoS One. 2022 Apr 27;17(4):e0266144. doi: 10.1371/journal.pone.0266144 (PMC9045630; doi:10.1371/journal.pone.0266144)

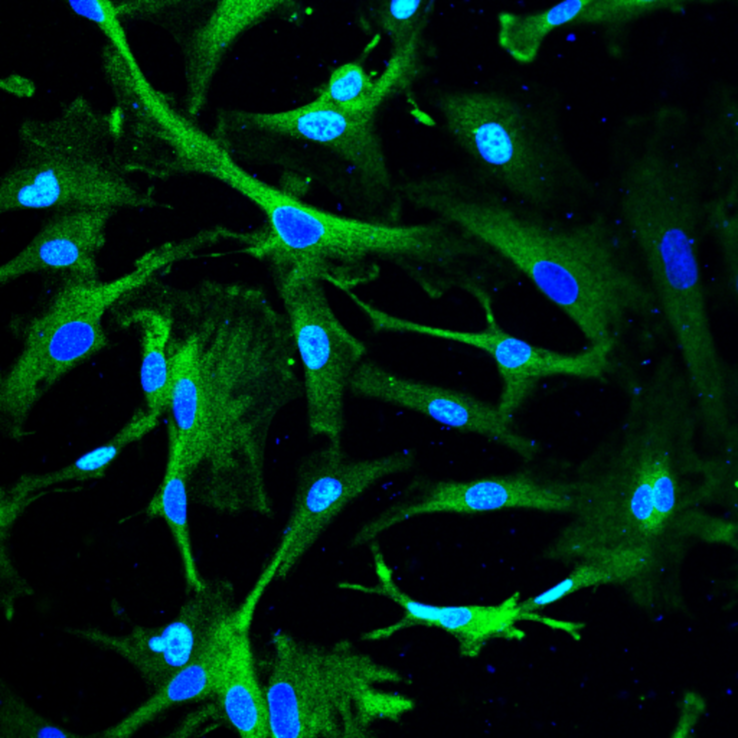

Supplement: S3 Appendix — (ZIP) [file pone.0266144.s003.zip › S3 Appendix/Immunofluorescence images/E.COLI+P4/E+P3Snapshot1/E+P3Snapshot1_RAW_ch00.tif]

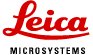

Supplement: S3 Appendix — (ZIP) [file pone.0266144.s003.zip › S3 Appendix/Immunofluorescence images/E.COLI+P4/E+P3Snapshot1/MetaData/LeicaLogo.jpg]

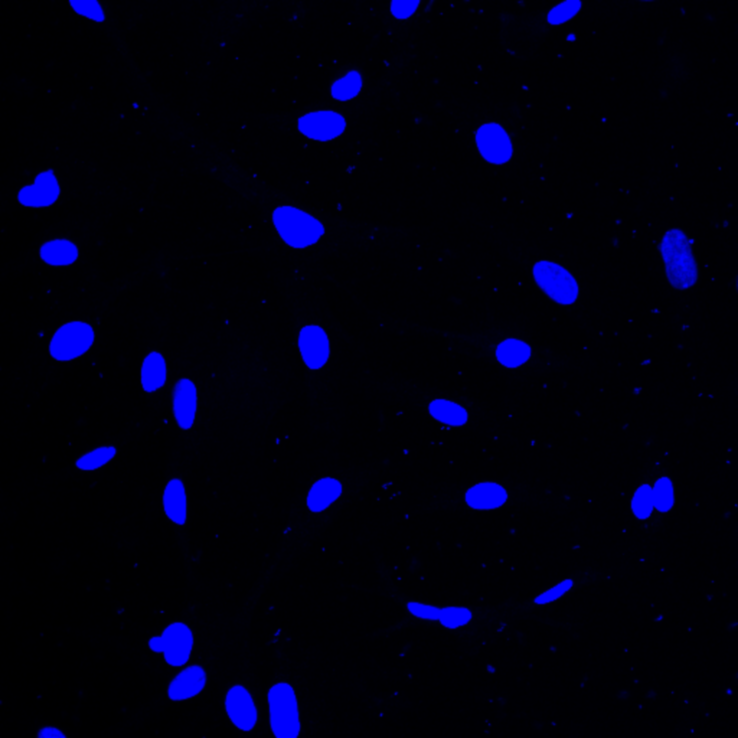

Supplement: S3 Appendix — (ZIP) [file pone.0266144.s003.zip › S3 Appendix/Immunofluorescence images/E.COLI+P4/E+P3Snapshot2/E+P3Snapshot2_RAW_ch00.tif]

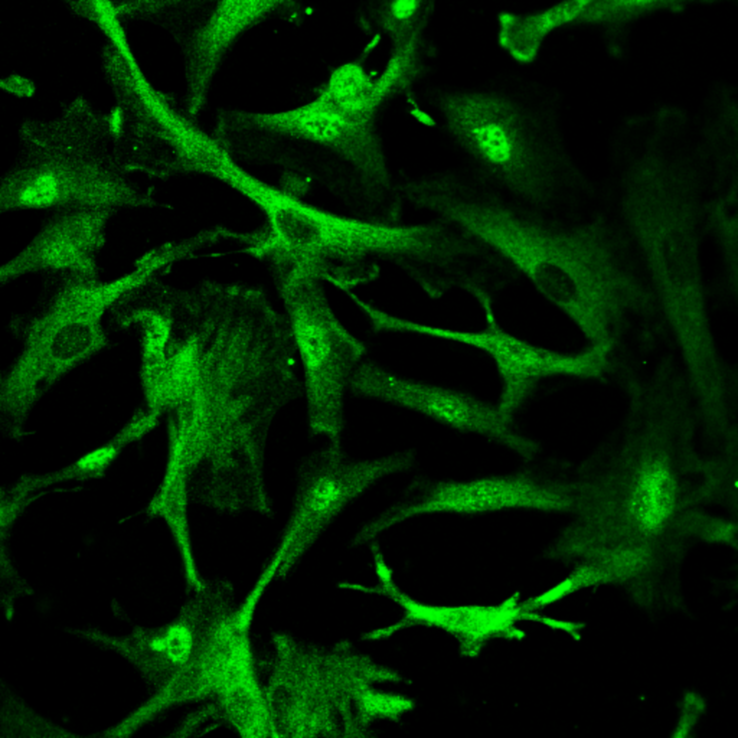

Supplement: S3 Appendix — (ZIP) [file pone.0266144.s003.zip › S3 Appendix/Immunofluorescence images/E.COLI+P4/E+P3Snapshot3/E+P3Snapshot3_RAW_ch00.tif]

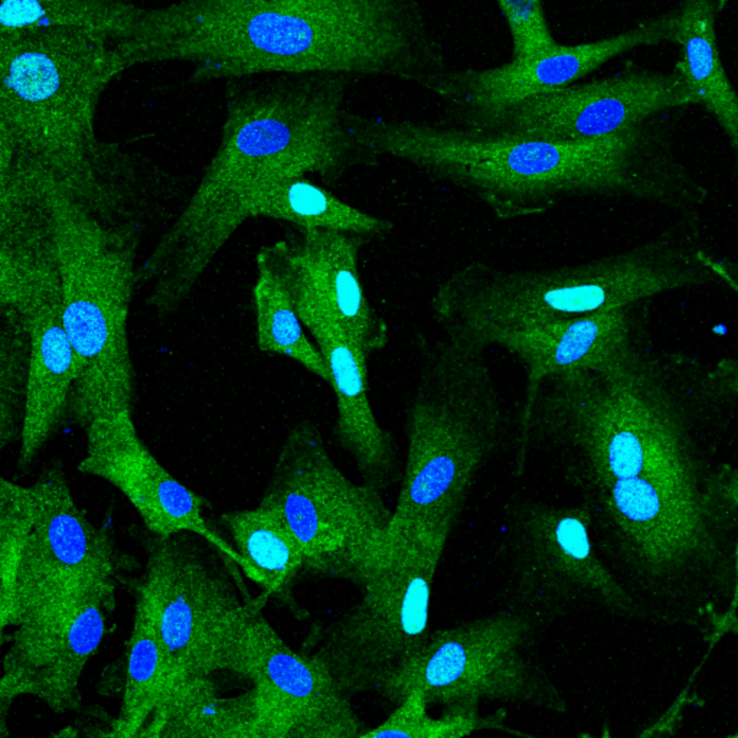

Supplement: S3 Appendix — (ZIP) [file pone.0266144.s003.zip › S3 Appendix/Immunofluorescence images/E.COLI+P4/E-4Snapshot1/E-4Snapshot1_RAW_ch00.tif]

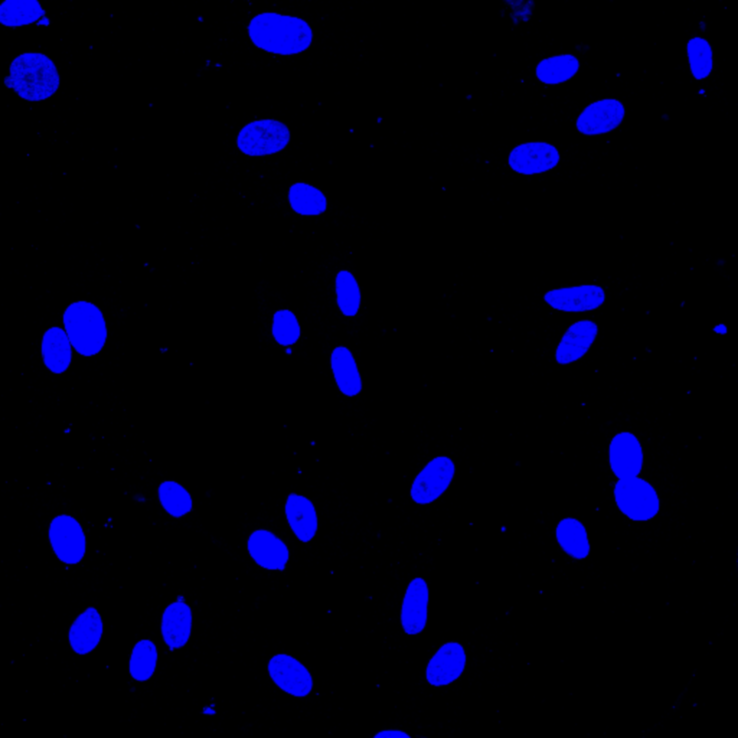

Supplement: S3 Appendix — (ZIP) [file pone.0266144.s003.zip › S3 Appendix/Immunofluorescence images/E.COLI+P4/E-4Snapshot2/E-4Snapshot2_RAW_ch00.tif]

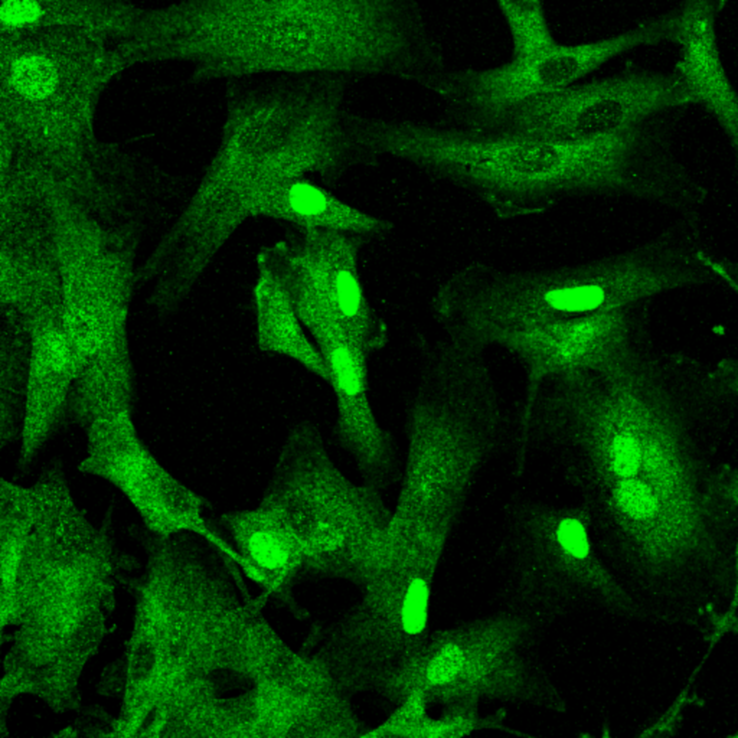

Supplement: S3 Appendix — (ZIP) [file pone.0266144.s003.zip › S3 Appendix/Immunofluorescence images/E.COLI+P4/E-4Snapshot3/E-4Snapshot3_RAW_ch00.tif]

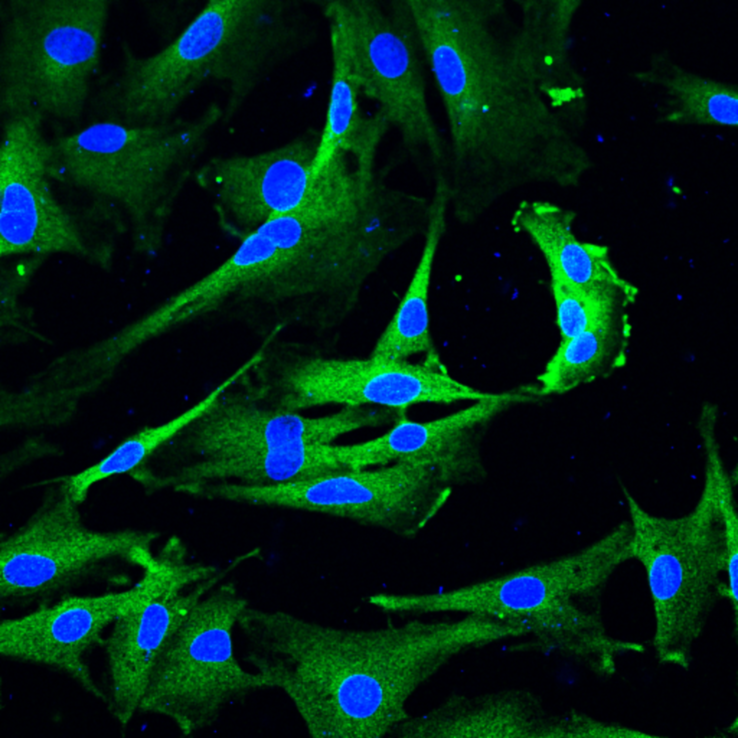

Supplement: S3 Appendix — (ZIP) [file pone.0266144.s003.zip › S3 Appendix/Immunofluorescence images/E.COLI+P4/K-E-1Snapshot1/K-E-1Snapshot1_RAW_ch00.tif]

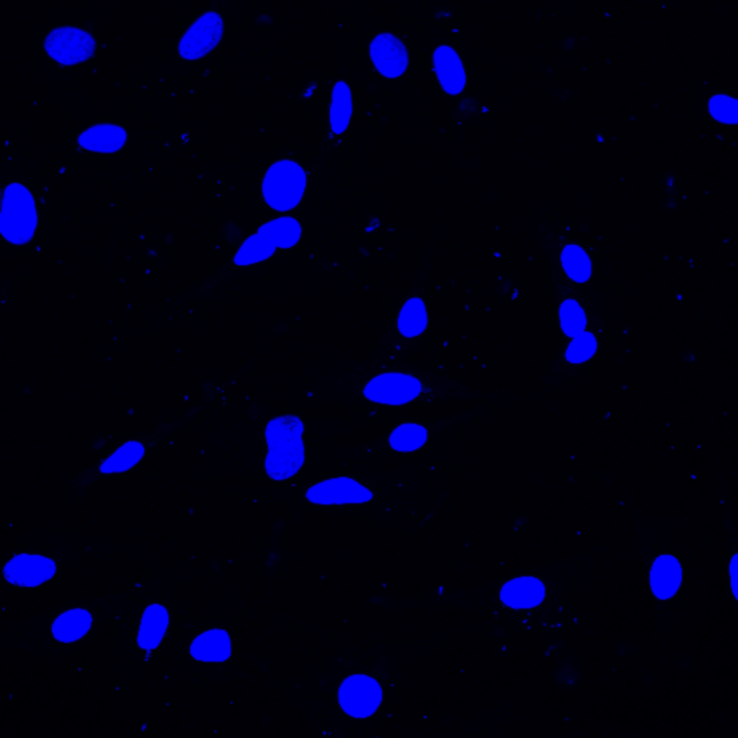

Supplement: S3 Appendix — (ZIP) [file pone.0266144.s003.zip › S3 Appendix/Immunofluorescence images/E.COLI+P4/K-E-1Snapshot2/K-E-1Snapshot2_RAW_ch00.tif]

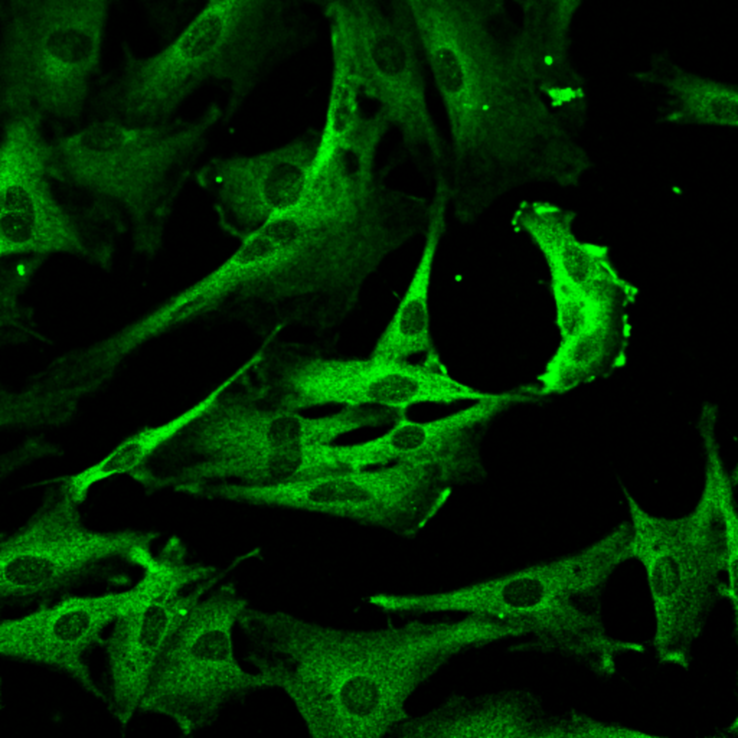

Supplement: S3 Appendix — (ZIP) [file pone.0266144.s003.zip › S3 Appendix/Immunofluorescence images/E.COLI+P4/K-E-1Snapshot3/K-E-1Snapshot3_RAW_ch00.tif]

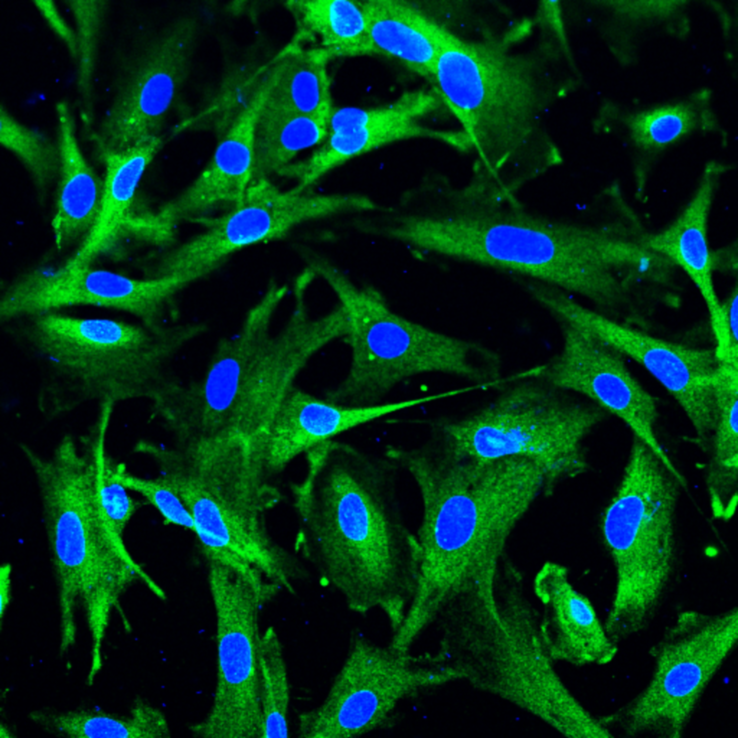

Supplement: S3 Appendix — (ZIP) [file pone.0266144.s003.zip › S3 Appendix/Immunofluorescence images/LPS+P4/K-L-1Snapshot1_000/K-L-1Snapshot1_000_RAW_ch00.tif]

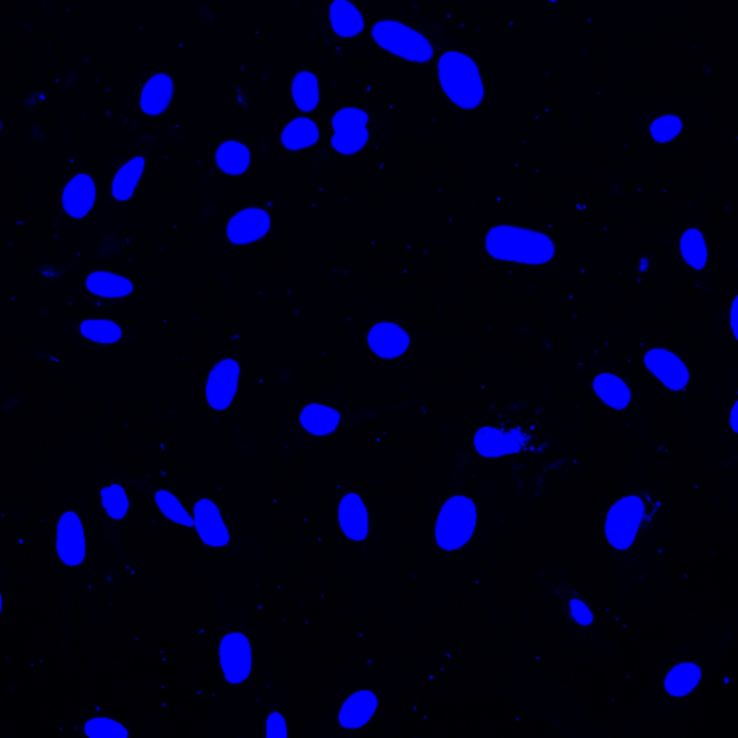

Supplement: S3 Appendix — (ZIP) [file pone.0266144.s003.zip › S3 Appendix/Immunofluorescence images/LPS+P4/K-L-1Snapshot2_000/K-L-1Snapshot2_000_RAW_ch00.tif]

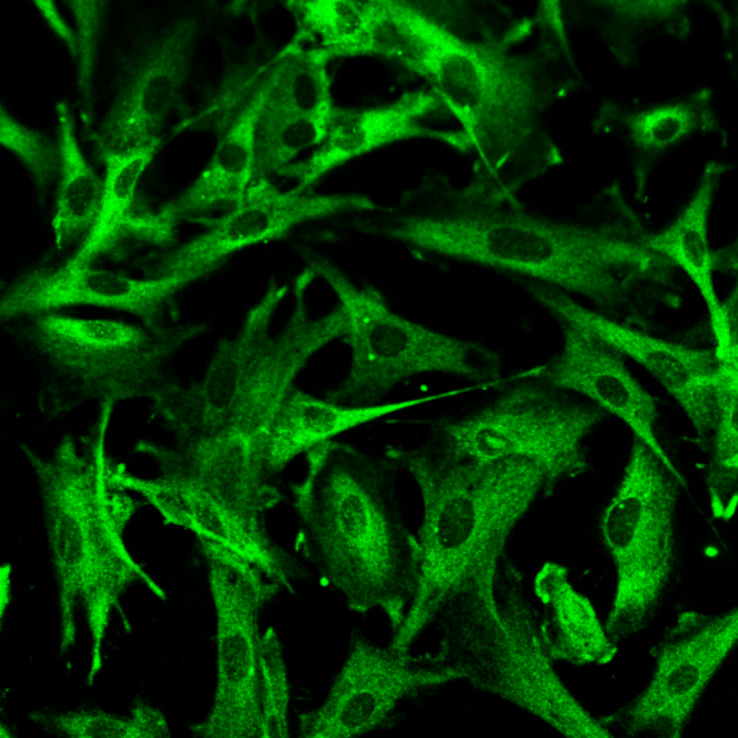

Supplement: S3 Appendix — (ZIP) [file pone.0266144.s003.zip › S3 Appendix/Immunofluorescence images/LPS+P4/K-L-1Snapshot3/K-L-1Snapshot3_RAW_ch00.tif]

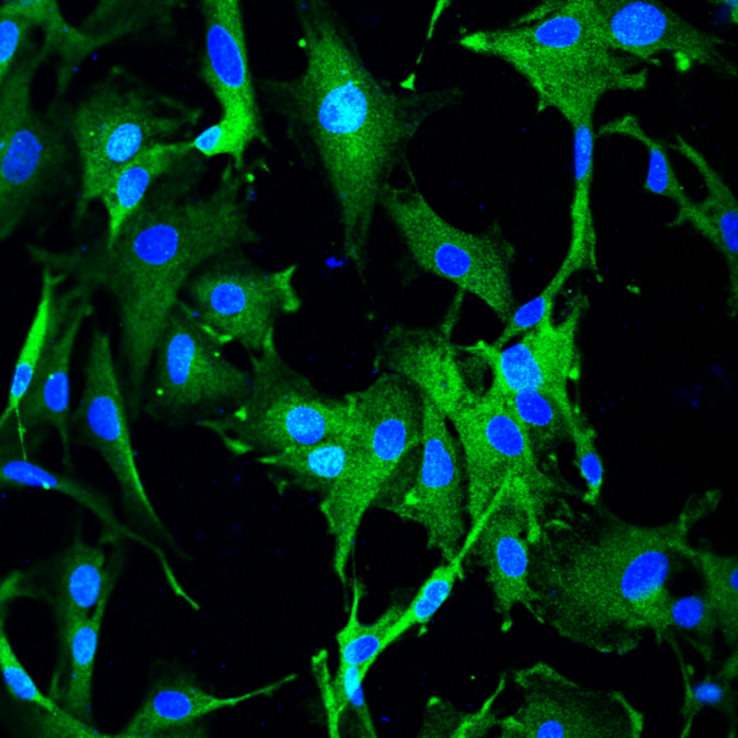

Supplement: S3 Appendix — (ZIP) [file pone.0266144.s003.zip › S3 Appendix/Immunofluorescence images/LPS+P4/L+P3-2Snapshot1/L+P3-2Snapshot1_RAW_ch00.tif]

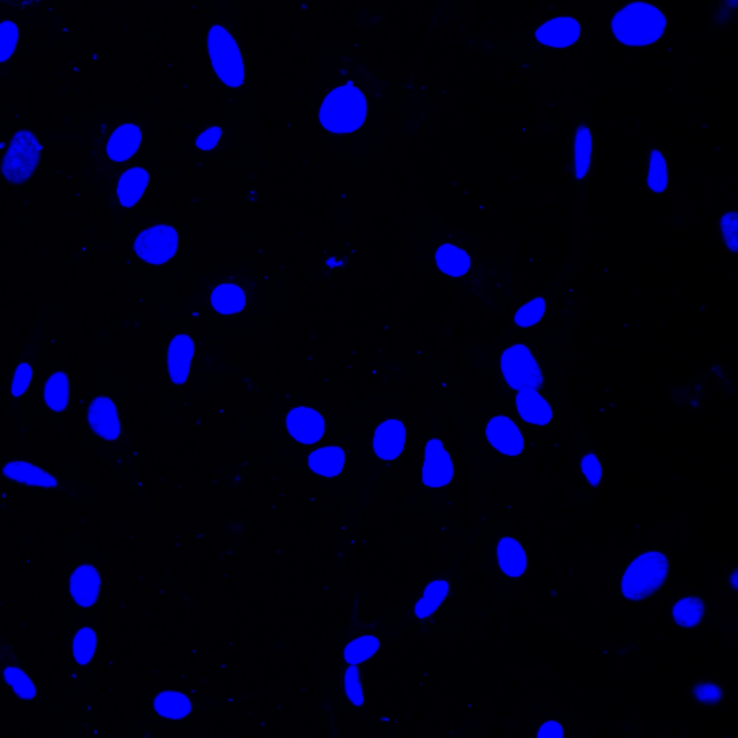

Supplement: S3 Appendix — (ZIP) [file pone.0266144.s003.zip › S3 Appendix/Immunofluorescence images/LPS+P4/L+P3-2Snapshot2/L+P3-2Snapshot2_RAW_ch00.tif]

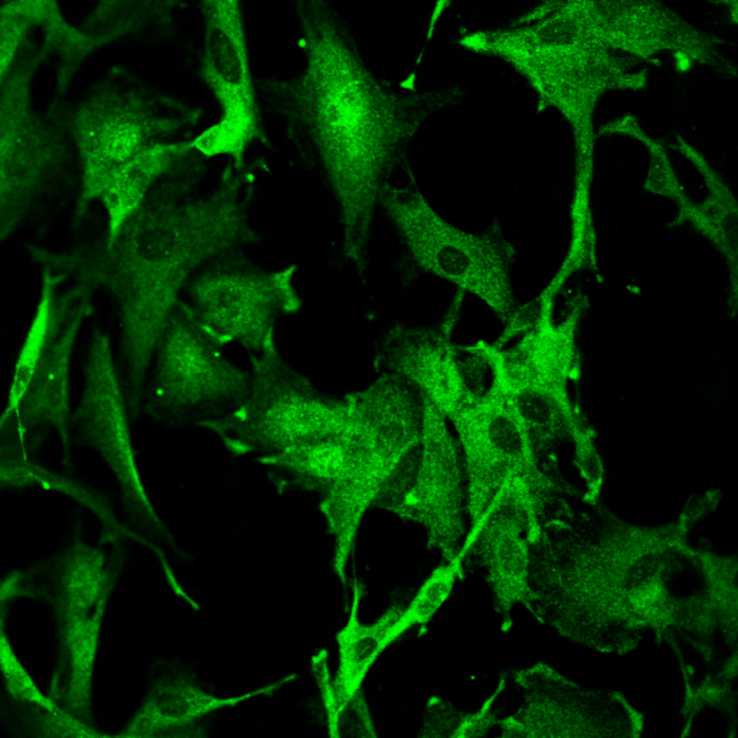

Supplement: S3 Appendix — (ZIP) [file pone.0266144.s003.zip › S3 Appendix/Immunofluorescence images/LPS+P4/L+P3-2Snapshot3/L+P3-2Snapshot3_RAW_ch00.tif]

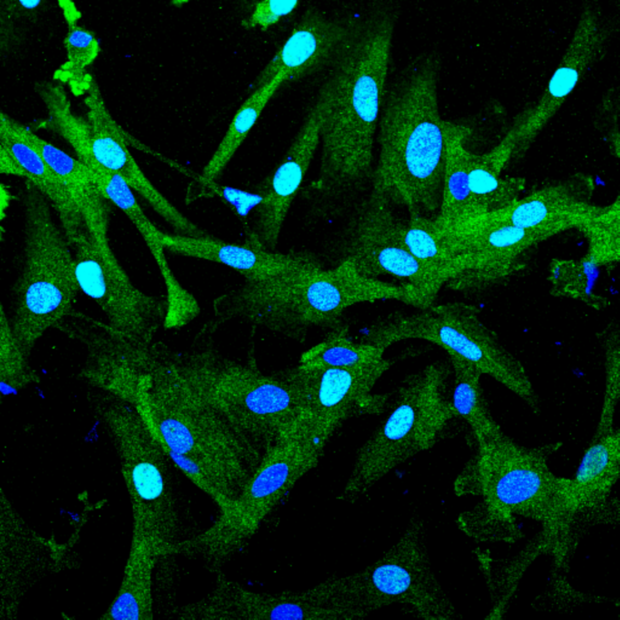

Supplement: S3 Appendix — (ZIP) [file pone.0266144.s003.zip › S3 Appendix/Immunofluorescence images/LPS+P4/L-3Snapshot1/L-3Snapshot1_RAW_ch00.tif]

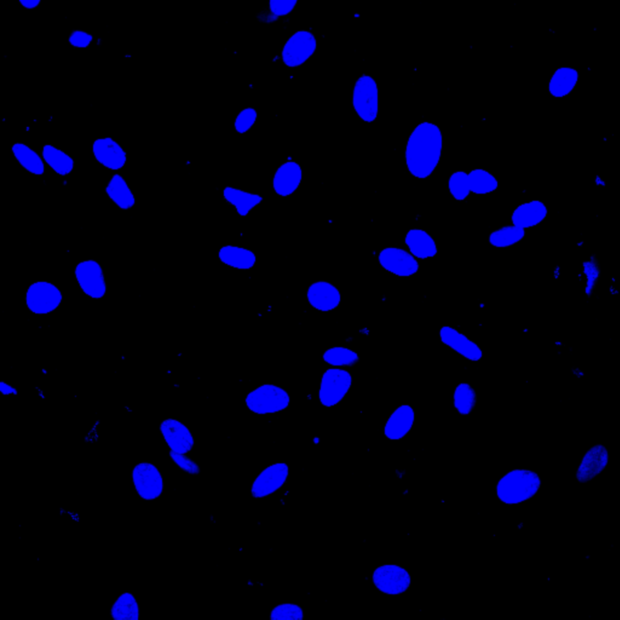

Supplement: S3 Appendix — (ZIP) [file pone.0266144.s003.zip › S3 Appendix/Immunofluorescence images/LPS+P4/L-3Snapshot2/L-3Snapshot2_RAW_ch00.tif]

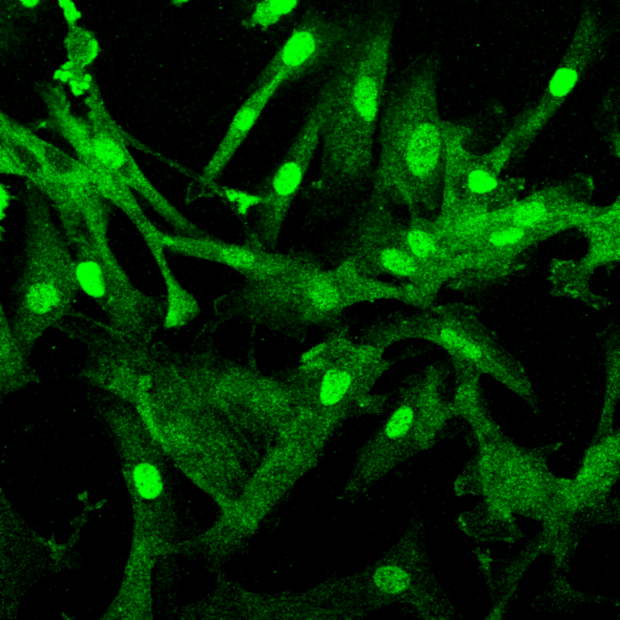

Supplement: S3 Appendix — (ZIP) [file pone.0266144.s003.zip › S3 Appendix/Immunofluorescence images/LPS+P4/L-3Snapshot3/L-3Snapshot3_RAW_ch00.tif]

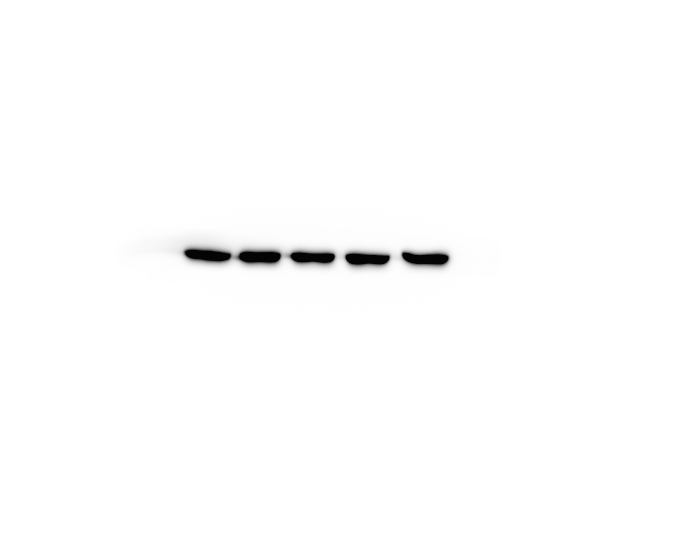

Supplement: S3 Appendix — (ZIP) [file pone.0266144.s003.zip › S3 Appendix/Western blot bands/WB E.COLI&P4-MAPK/BA_1_P38-210404_134630_00.09.000_2_65554.tif]

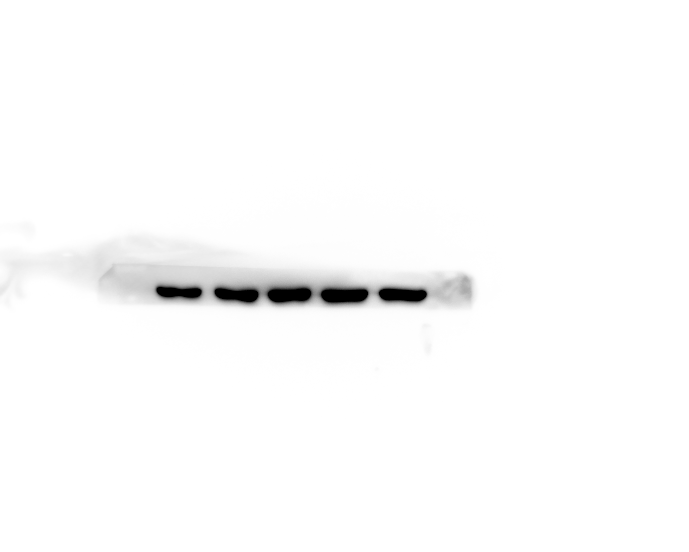

Supplement: S3 Appendix — (ZIP) [file pone.0266144.s003.zip › S3 Appendix/Western blot bands/WB E.COLI&P4-MAPK/BA_2_ERK-30_210330_165417_00.10.000_1_32778.tif]

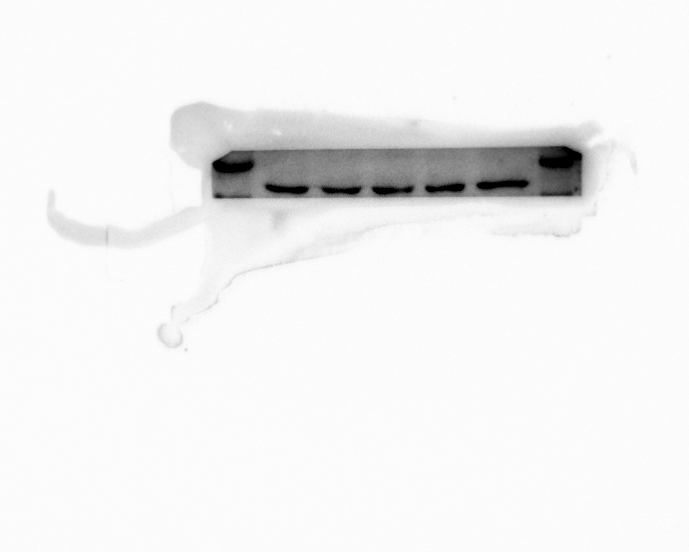

Supplement: S3 Appendix — (ZIP) [file pone.0266144.s003.zip › S3 Appendix/Western blot bands/WB E.COLI&P4-MAPK/BA_2_JNK-210424_165039_01.05.000_1_3278.tif]

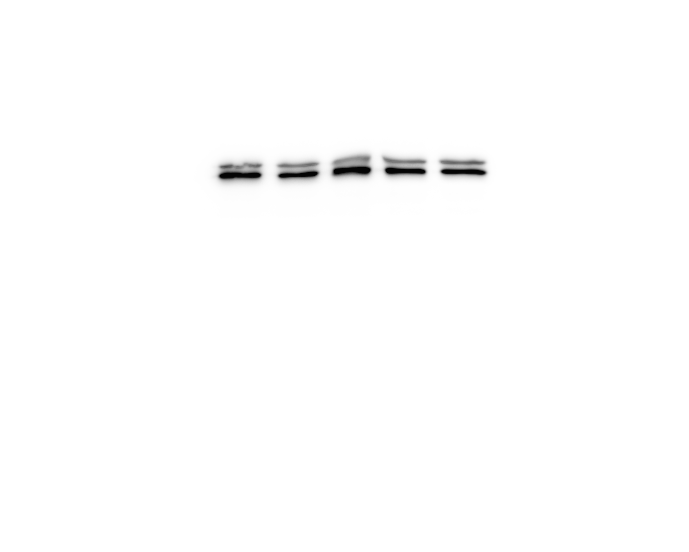

Supplement: S3 Appendix — (ZIP) [file pone.0266144.s003.zip › S3 Appendix/Western blot bands/WB E.COLI&P4-MAPK/ERK_1_210410_151309_00.01.000_2_65580.tif]

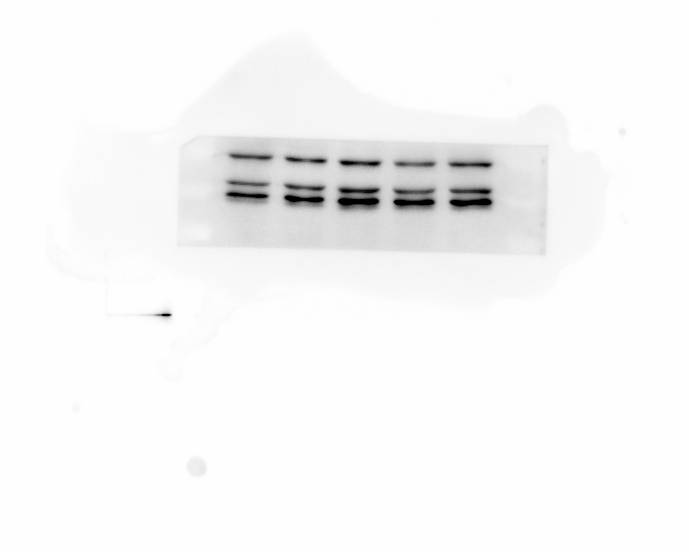

Supplement: S3 Appendix — (ZIP) [file pone.0266144.s003.zip › S3 Appendix/Western blot bands/WB E.COLI&P4-MAPK/JNK_1_210424_171117_00.31.000_1_9467.tif]

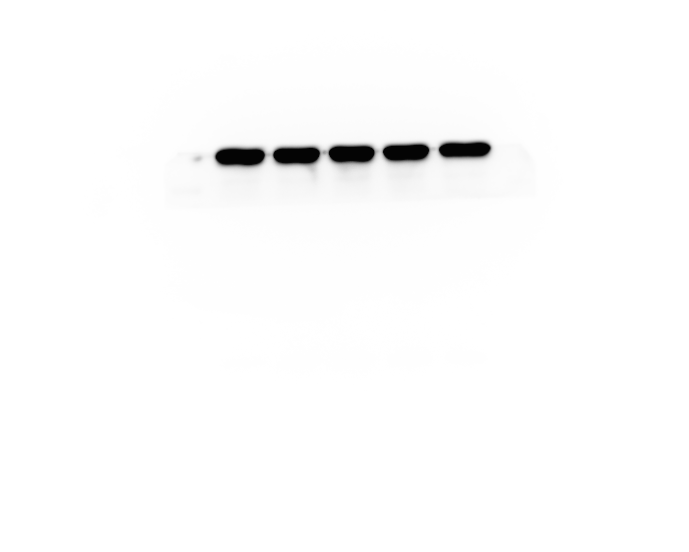

Supplement: S3 Appendix — (ZIP) [file pone.0266144.s003.zip › S3 Appendix/Western blot bands/WB E.COLI&P4-MAPK/P38_2_210406_172406_00.27.000_3_131080.tif]

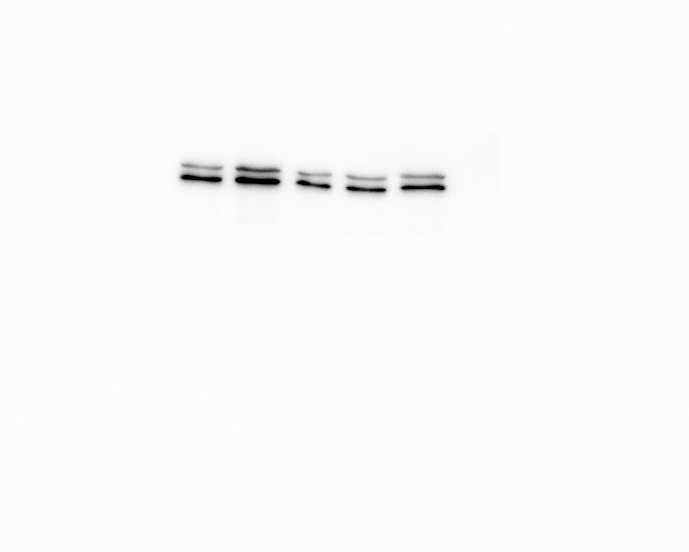

Supplement: S3 Appendix — (ZIP) [file pone.0266144.s003.zip › S3 Appendix/Western blot bands/WB E.COLI&P4-MAPK/PERK_2_2_210412_075556_00.01.000_1_5452.tif]

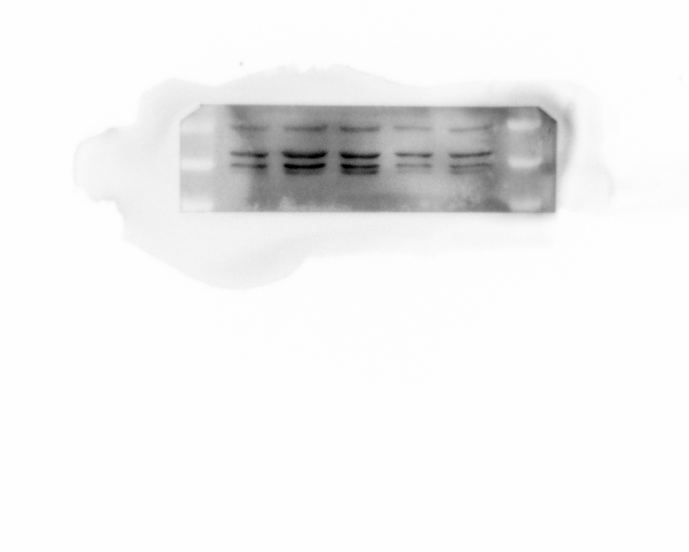

Supplement: S3 Appendix — (ZIP) [file pone.0266144.s003.zip › S3 Appendix/Western blot bands/WB E.COLI&P4-MAPK/PJNK_4_210425_152502_00.31.000_2_7902.tif]

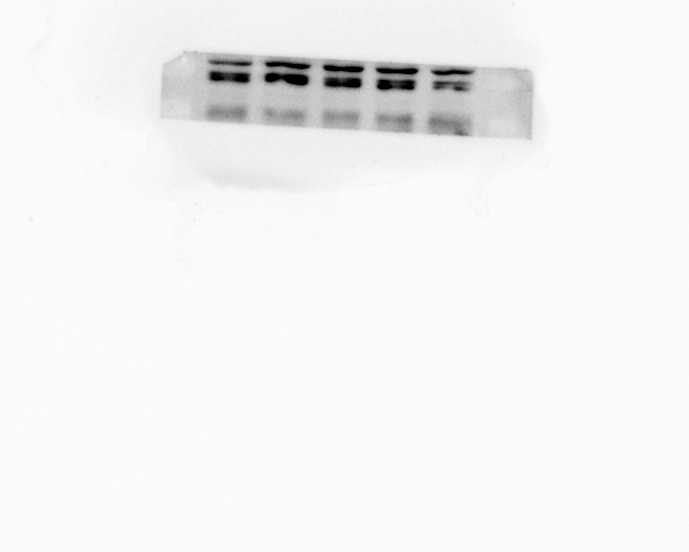

Supplement: S3 Appendix — (ZIP) [file pone.0266144.s003.zip › S3 Appendix/Western blot bands/WB E.COLI&P4-MAPK/PP38_1_210406_171916_00.27.000_1_2989.tif]

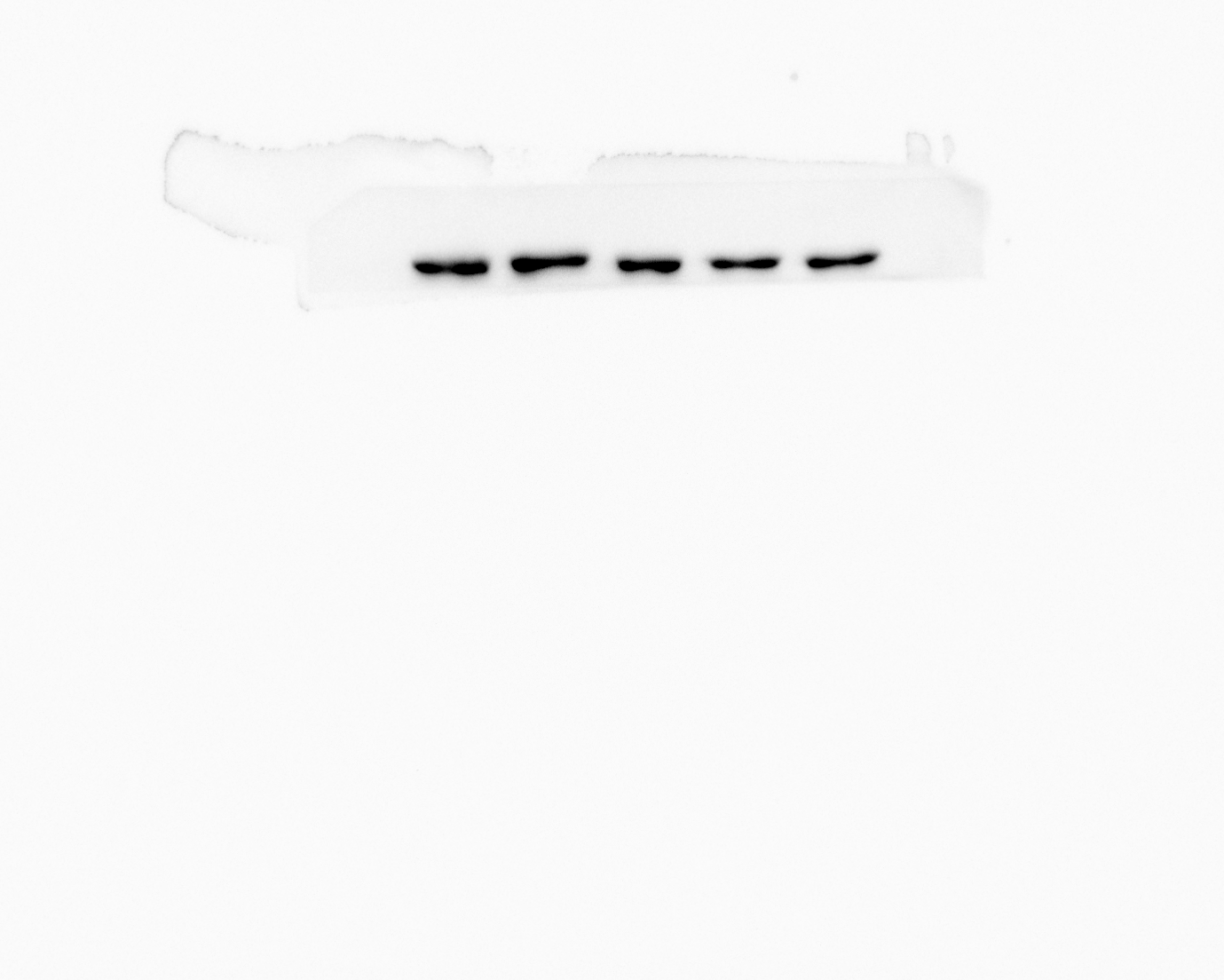

Supplement: S3 Appendix — (ZIP) [file pone.0266144.s003.zip › S3 Appendix/Western blot bands/WB E.COLI&P4-NFKB/BA_1_IKB-210509_152248_01.15.000_1_2218.tif]

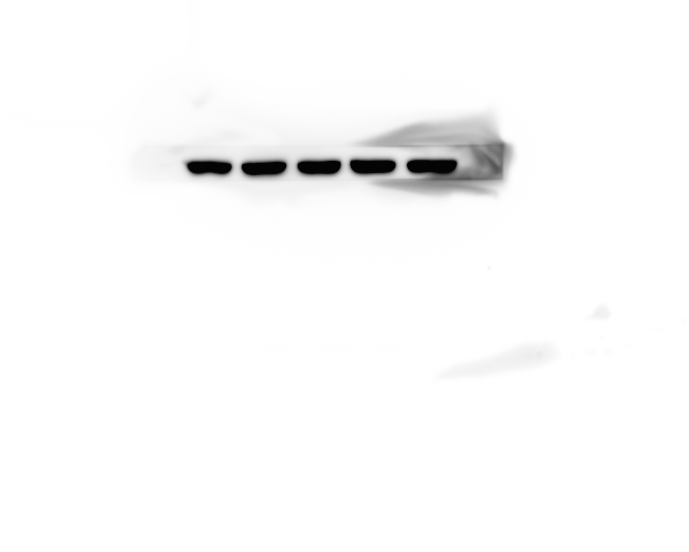

Supplement: S3 Appendix — (ZIP) [file pone.0266144.s003.zip › S3 Appendix/Western blot bands/WB E.COLI&P4-NFKB/BA_2_P65-210402_102357_00.13.000_2_65540.tif]

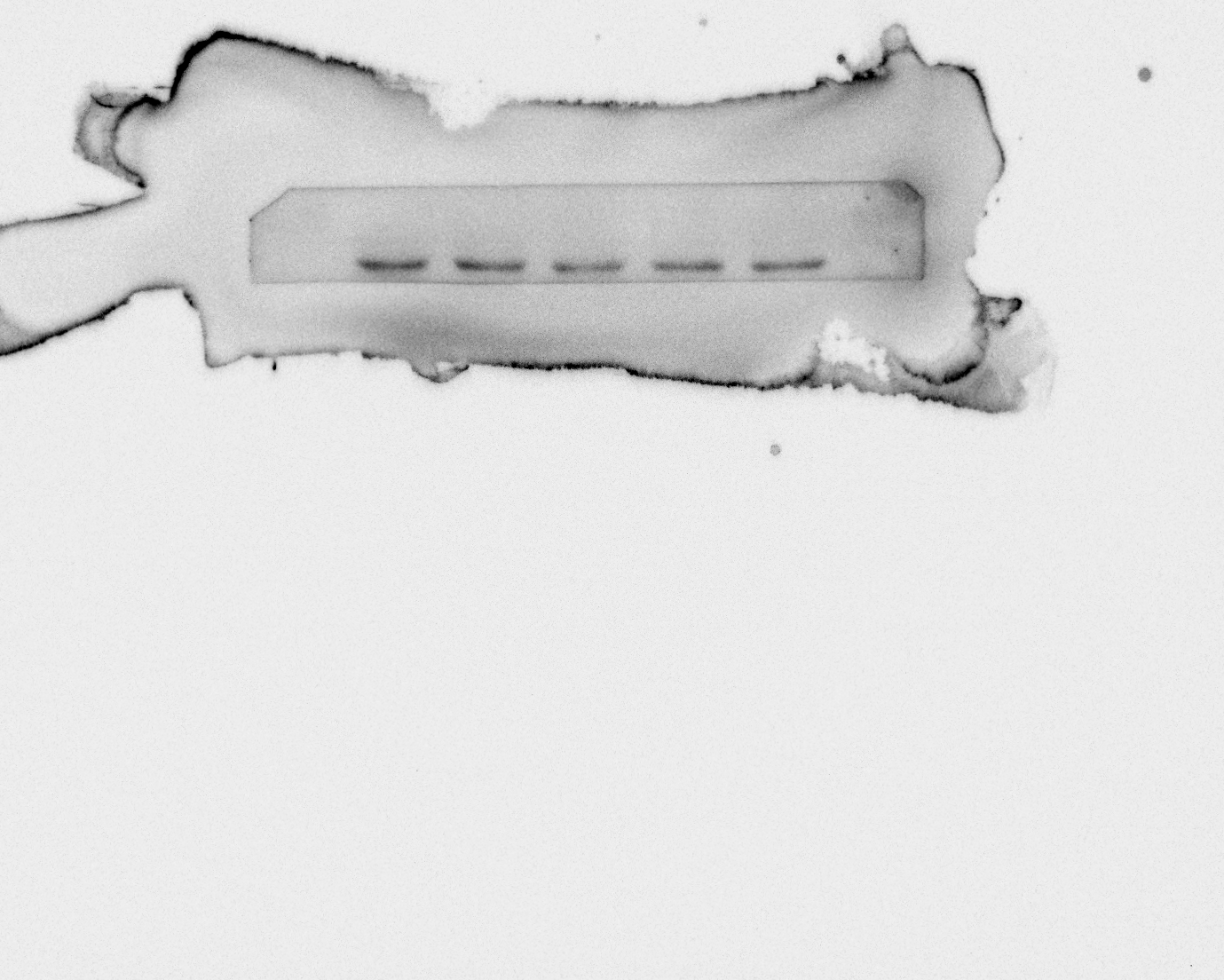

Supplement: S3 Appendix — (ZIP) [file pone.0266144.s003.zip › S3 Appendix/Western blot bands/WB E.COLI&P4-NFKB/IKB_3_210509_153521_01.15.000_2_600.tif]

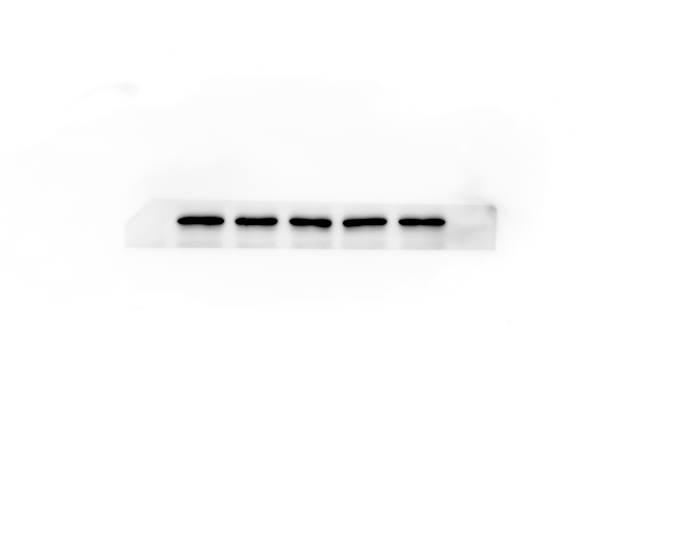

Supplement: S3 Appendix — (ZIP) [file pone.0266144.s003.zip › S3 Appendix/Western blot bands/WB E.COLI&P4-NFKB/P65_2_210402_104228_00.20.000_1_32783.tif]

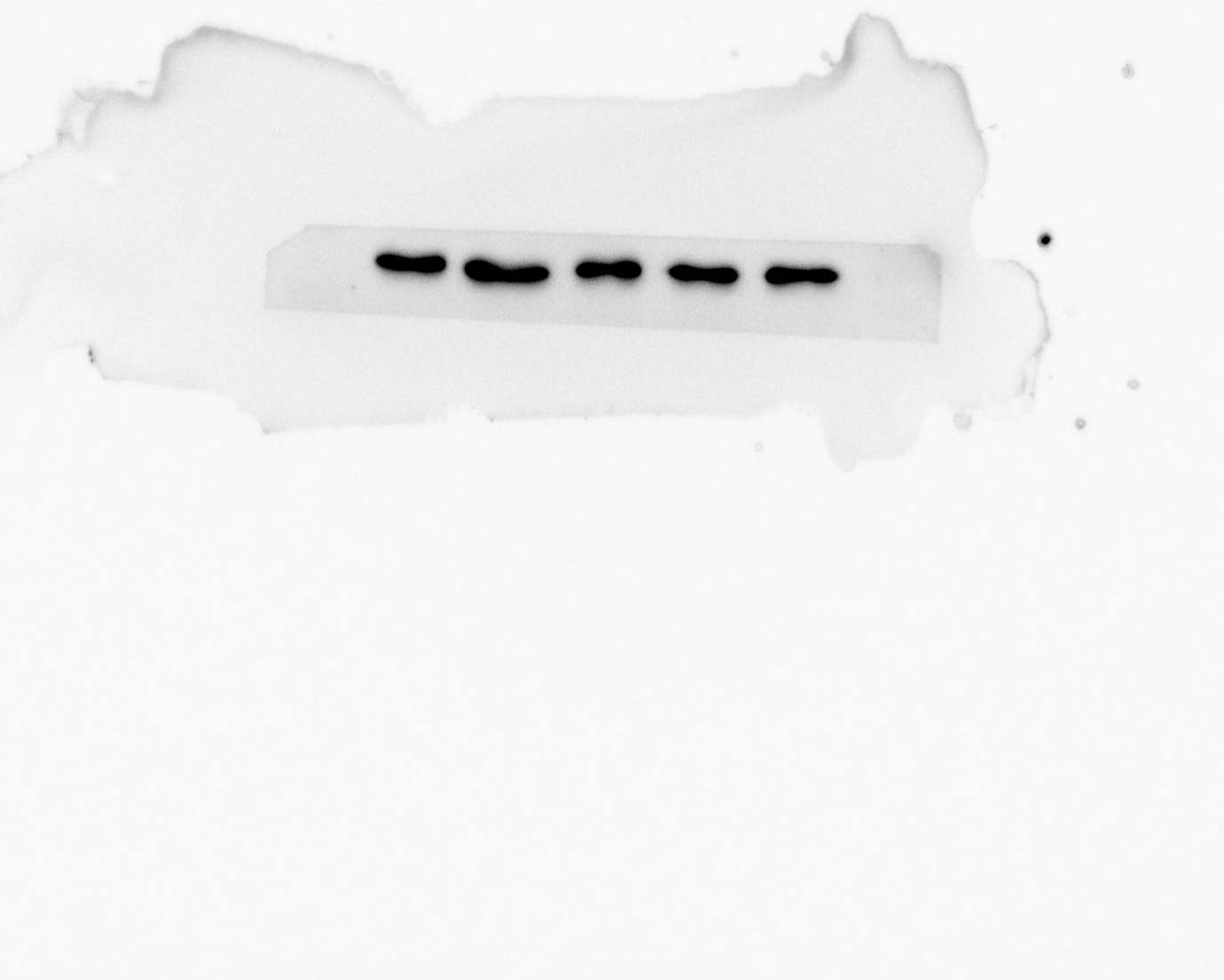

Supplement: S3 Appendix — (ZIP) [file pone.0266144.s003.zip › S3 Appendix/Western blot bands/WB E.COLI&P4-NFKB/PIKB_7_210509_155733_01.15.000_1_1788.tif]

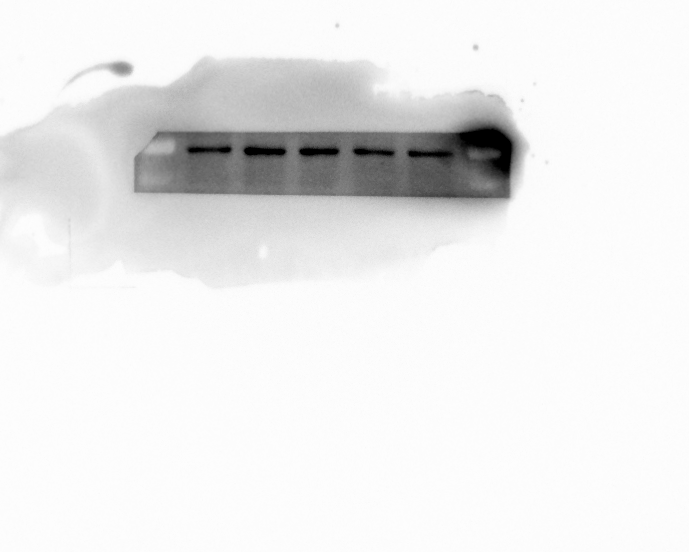

Supplement: S3 Appendix — (ZIP) [file pone.0266144.s003.zip › S3 Appendix/Western blot bands/WB E.COLI&P4-NFKB/PP65_2_210402_103848_00.33.000_1_4273.tif]

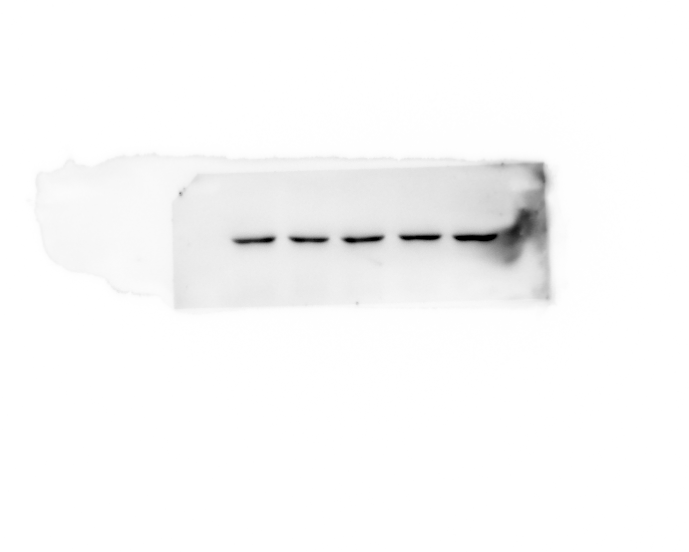

Supplement: S3 Appendix — (ZIP) [file pone.0266144.s003.zip › S3 Appendix/Western blot bands/WB E.COLI-MAPK/BA_1_211006_161736_02.50.000_2_19984.tif]

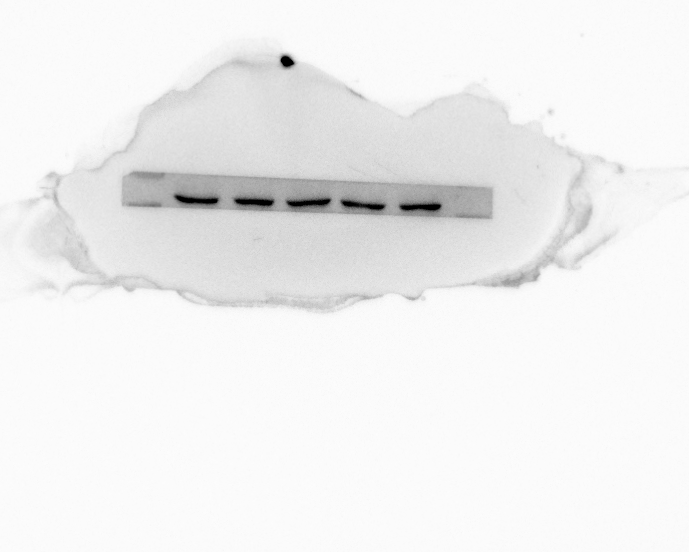

Supplement: S3 Appendix — (ZIP) [file pone.0266144.s003.zip › S3 Appendix/Western blot bands/WB E.COLI-MAPK/BA_1_JNK-210928_151950_00.37.000_1_3000.tif]

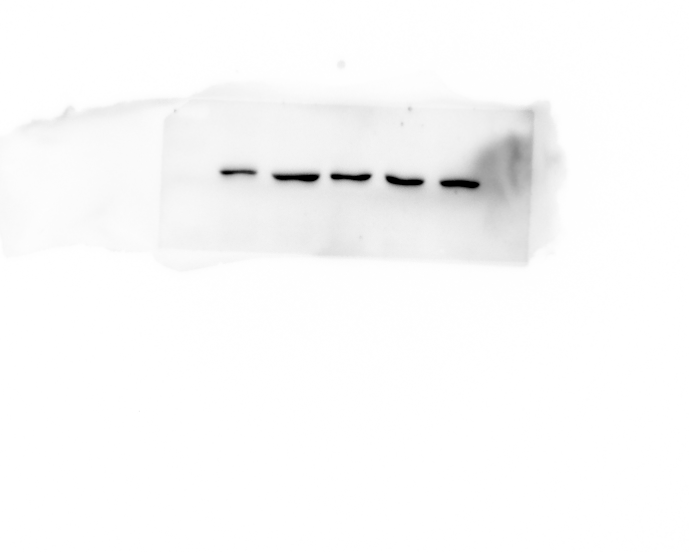

Supplement: S3 Appendix — (ZIP) [file pone.0266144.s003.zip › S3 Appendix/Western blot bands/WB E.COLI-MAPK/BA_2_P38-211006_162209_01.40.000_1_15938.tif]

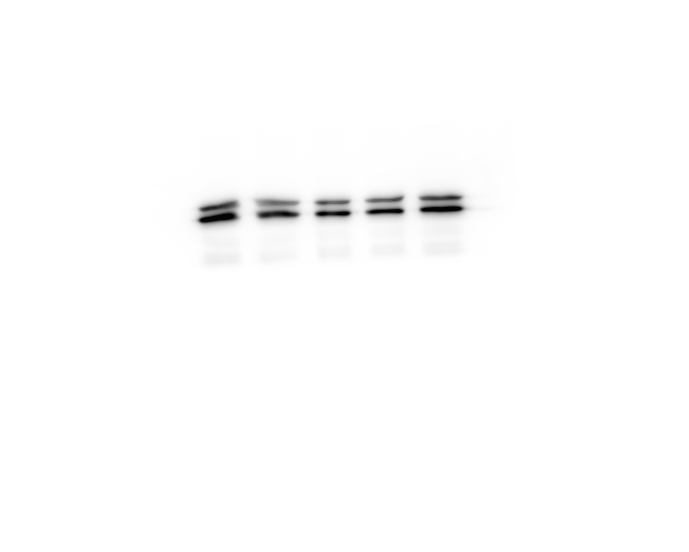

Supplement: S3 Appendix — (ZIP) [file pone.0266144.s003.zip › S3 Appendix/Western blot bands/WB E.COLI-MAPK/ERK_2_211006_162803_00.01.000_2_65604.tif]

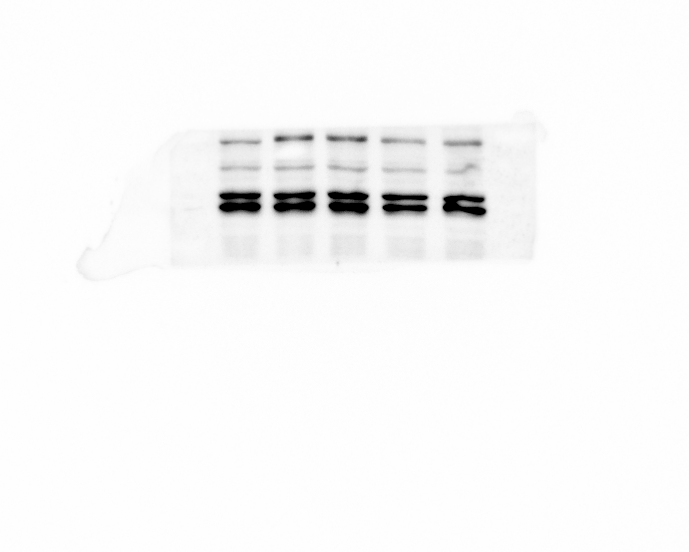

Supplement: S3 Appendix — (ZIP) [file pone.0266144.s003.zip › S3 Appendix/Western blot bands/WB E.COLI-MAPK/JNK_1_210928_150436_00.10.000_1_6605.tif]

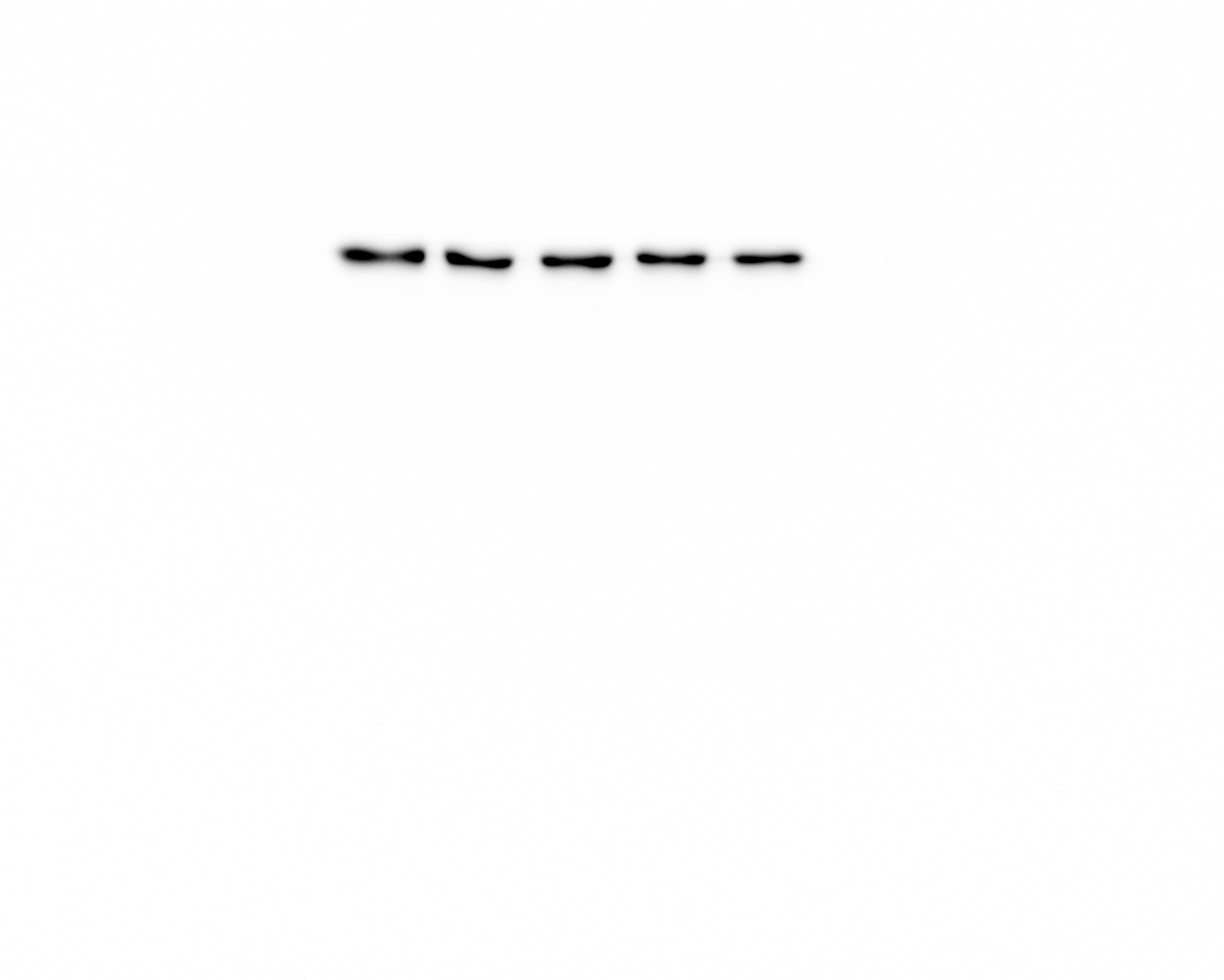

Supplement: S3 Appendix — (ZIP) [file pone.0266144.s003.zip › S3 Appendix/Western blot bands/WB E.COLI-MAPK/P38_2_210929_183538_00.02.000_1_13734.tif]

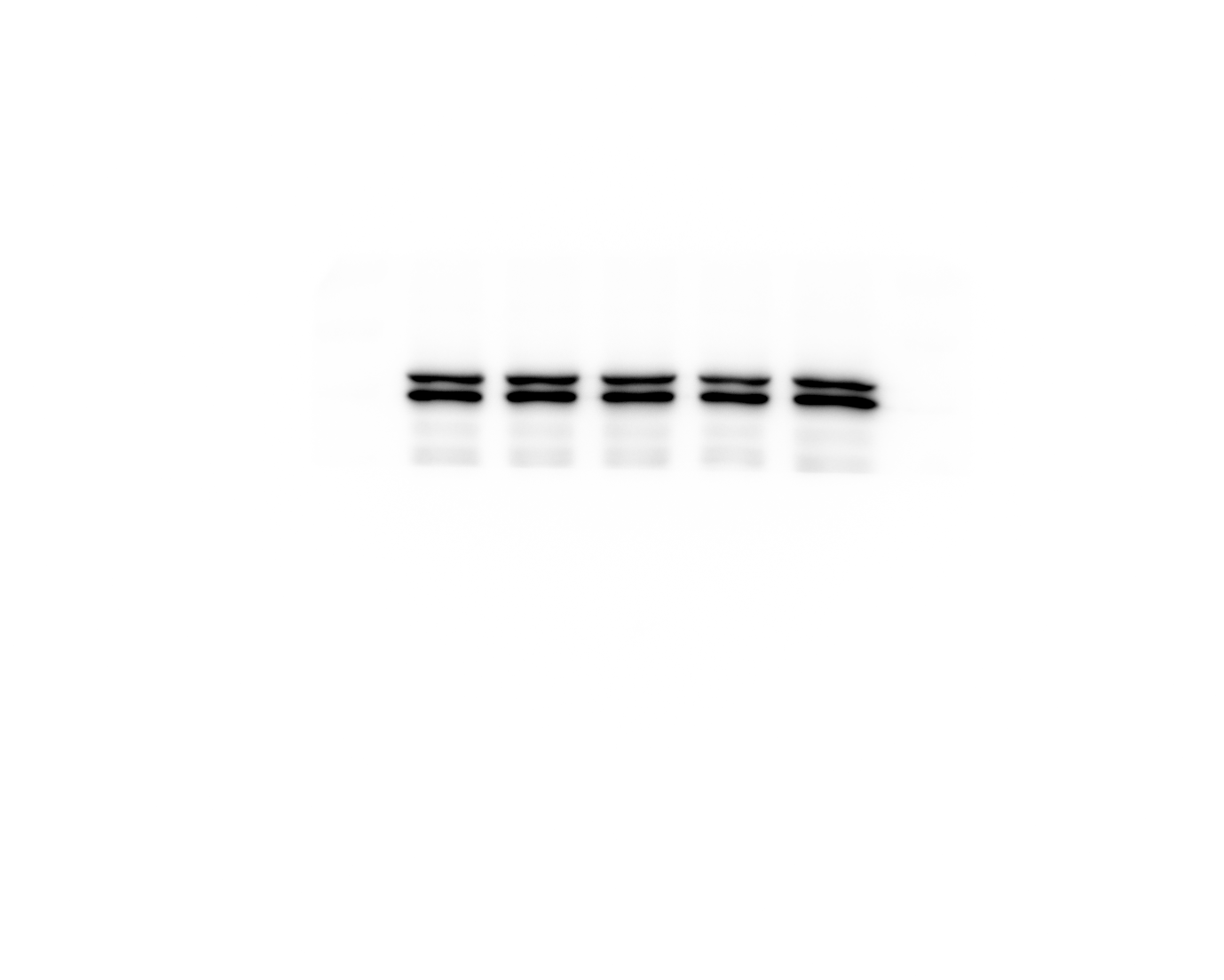

Supplement: S3 Appendix — (ZIP) [file pone.0266144.s003.zip › S3 Appendix/Western blot bands/WB E.COLI-MAPK/PERK_1_210929_180324_00.01.000_1_21706.tif]

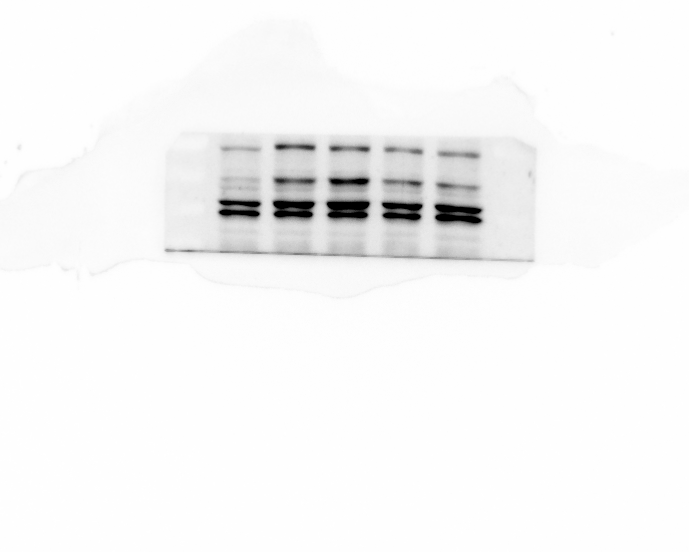

Supplement: S3 Appendix — (ZIP) [file pone.0266144.s003.zip › S3 Appendix/Western blot bands/WB E.COLI-MAPK/PJNK_1_210928_150818_00.23.000_1_8578.tif]

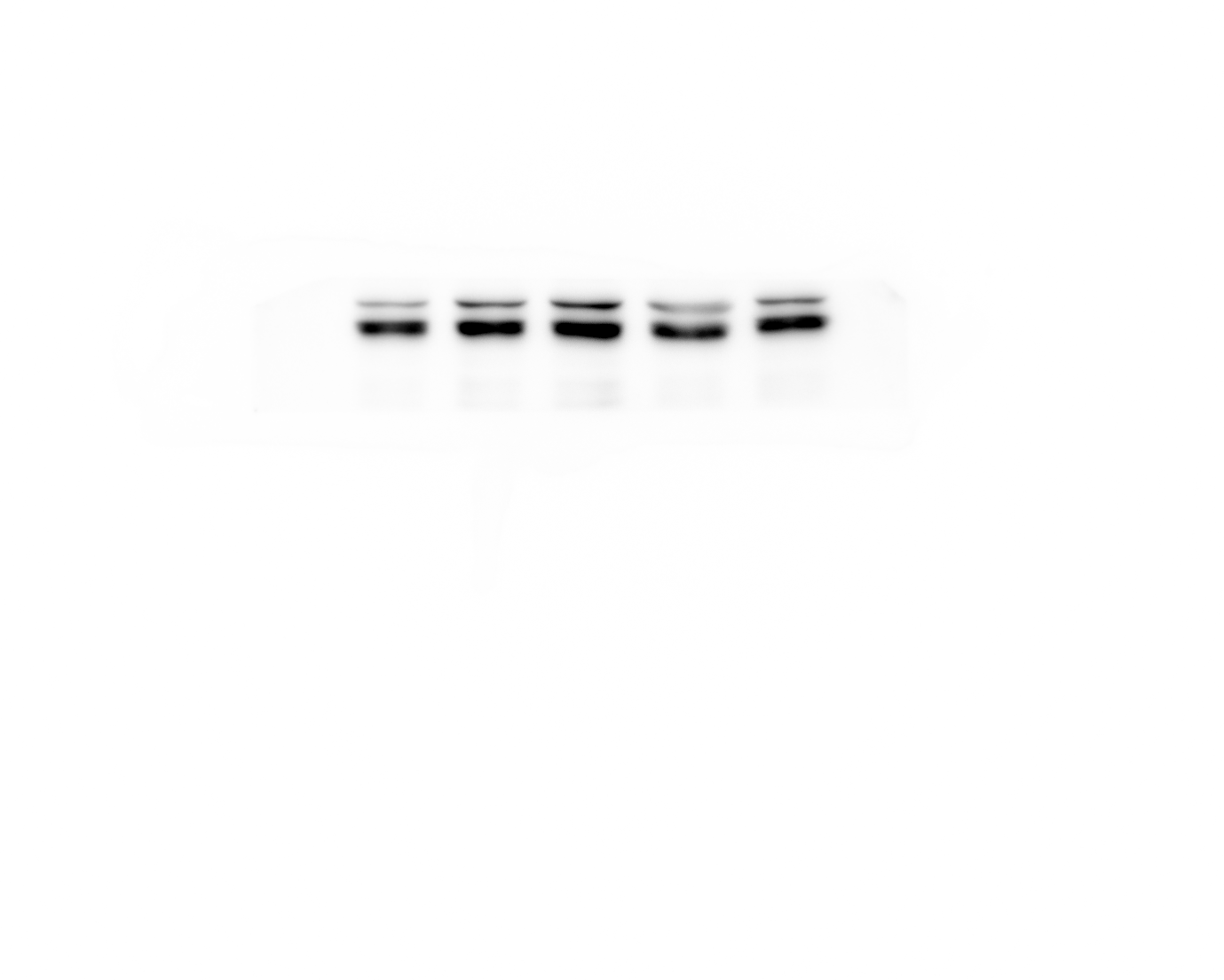

Supplement: S3 Appendix — (ZIP) [file pone.0266144.s003.zip › S3 Appendix/Western blot bands/WB E.COLI-MAPK/PP38_1_210929_183745_00.17.000_1_16557.tif]

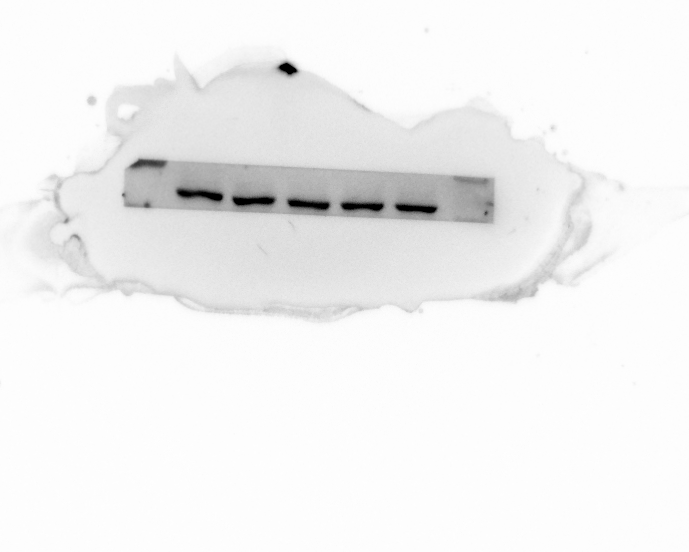

Supplement: S3 Appendix — (ZIP) [file pone.0266144.s003.zip › S3 Appendix/Western blot bands/WB E.COLI-NFKB/BA_2_IKB-210928_152130_00.55.000_1_5000.tif]

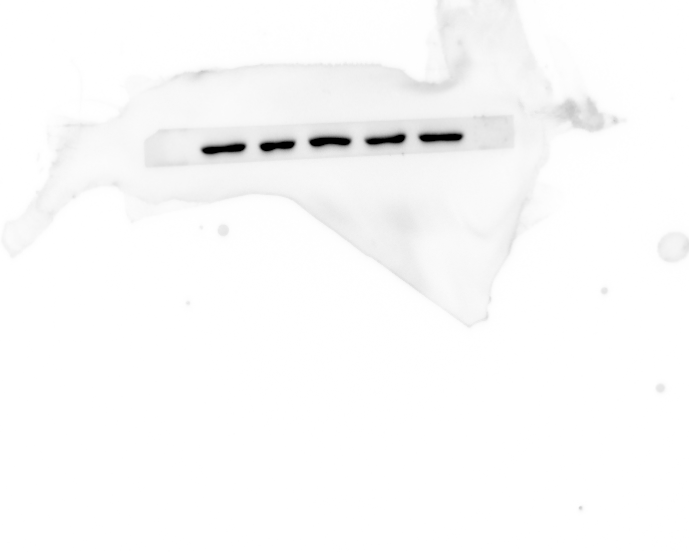

Supplement: S3 Appendix — (ZIP) [file pone.0266144.s003.zip › S3 Appendix/Western blot bands/WB E.COLI-NFKB/BA_2_P65-211002_103743_01.35.000_1_17539.tif]

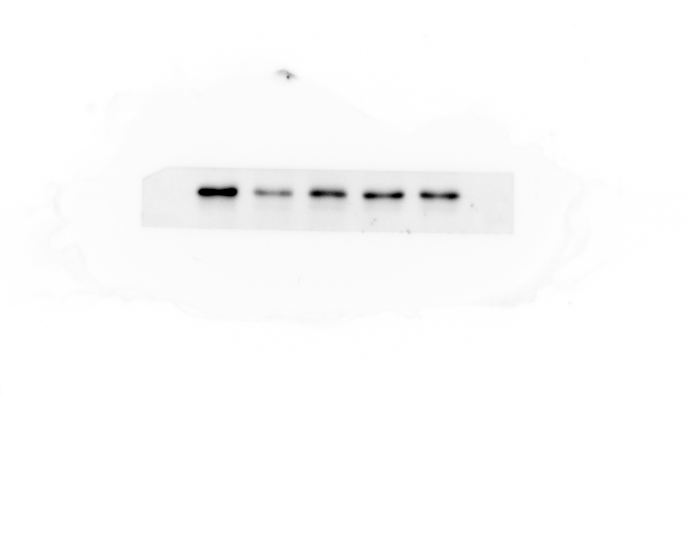

Supplement: S3 Appendix — (ZIP) [file pone.0266144.s003.zip › S3 Appendix/Western blot bands/WB E.COLI-NFKB/IKB_1_210928_152341_00.25.000_1_15737.tif]

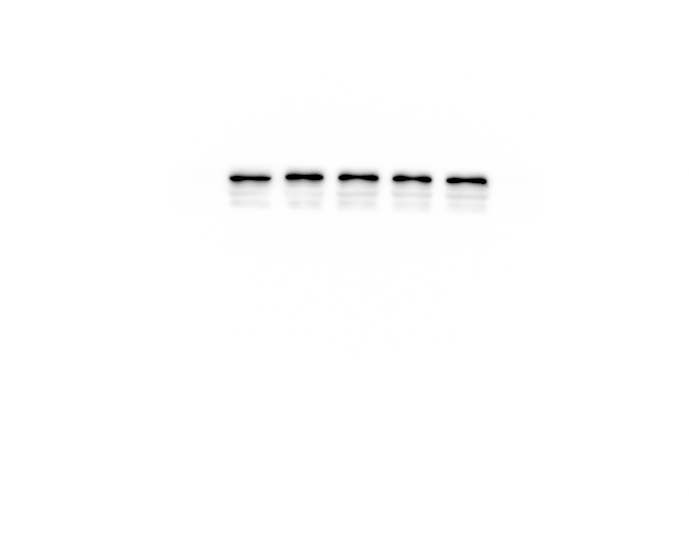

Supplement: S3 Appendix — (ZIP) [file pone.0266144.s003.zip › S3 Appendix/Western blot bands/WB E.COLI-NFKB/P65_1_211002_102843_00.01.000_1_18633.tif]

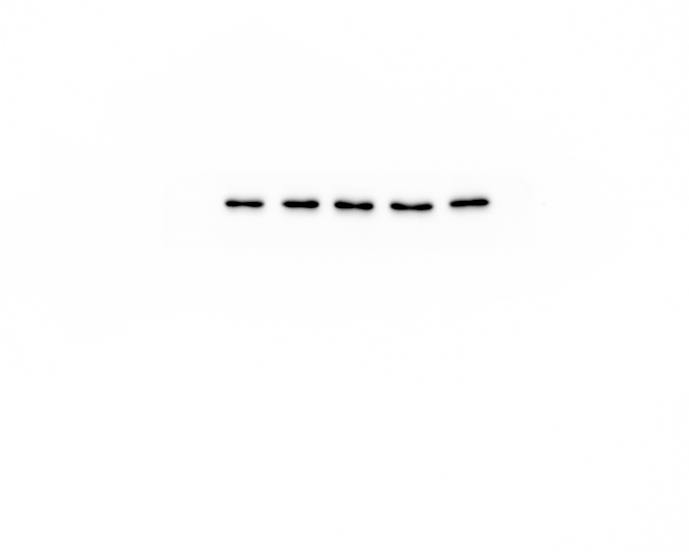

Supplement: S3 Appendix — (ZIP) [file pone.0266144.s003.zip › S3 Appendix/Western blot bands/WB E.COLI-NFKB/PIKB_1_210928_152644_00.03.000_1_13477.tif]

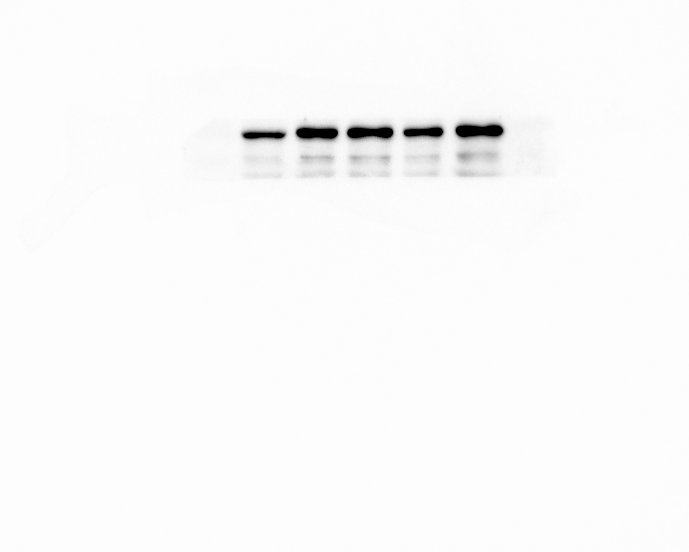

Supplement: S3 Appendix — (ZIP) [file pone.0266144.s003.zip › S3 Appendix/Western blot bands/WB E.COLI-NFKB/PP65_1_211002_103159_00.02.000_1_5000.tif]

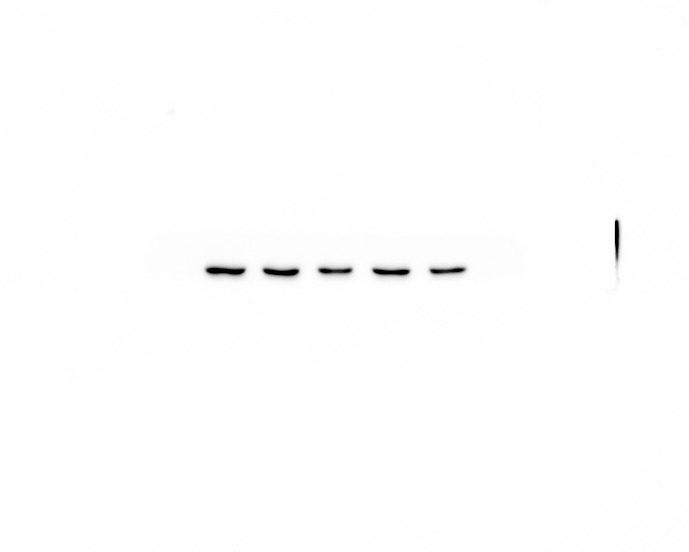

Supplement: S3 Appendix — (ZIP) [file pone.0266144.s003.zip › S3 Appendix/Western blot bands/WB LPS&P4 NFKB/BA_1_IKB-201231_132621_00.06.000_1_13869.tif]

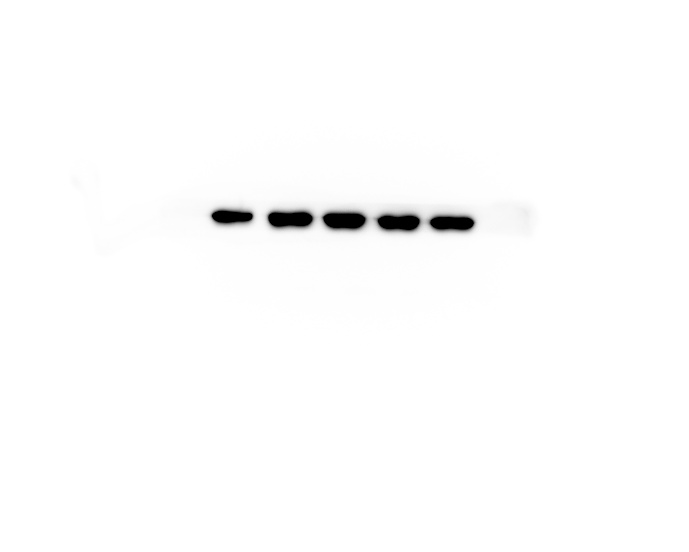

Supplement: S3 Appendix — (ZIP) [file pone.0266144.s003.zip › S3 Appendix/Western blot bands/WB LPS&P4 NFKB/BA_2_201205_114126_00.09.000_1_32774.jpg]

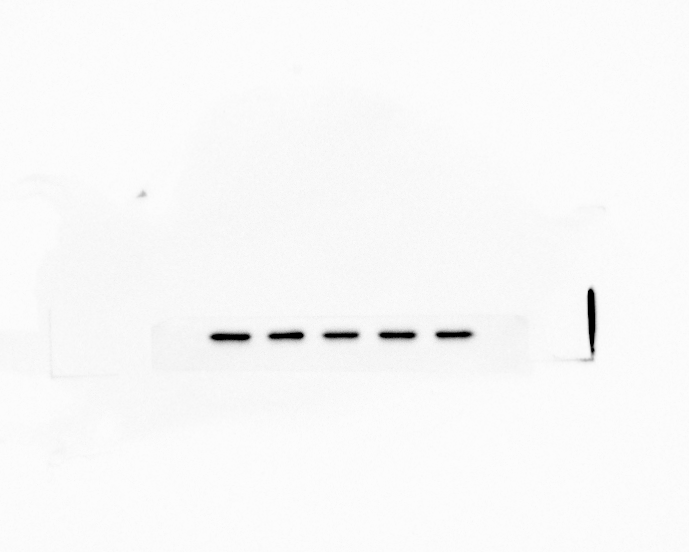

Supplement: S3 Appendix — (ZIP) [file pone.0266144.s003.zip › S3 Appendix/Western blot bands/WB LPS&P4 NFKB/IKB_2_201231_132412_00.08.000_1_3000.tif]

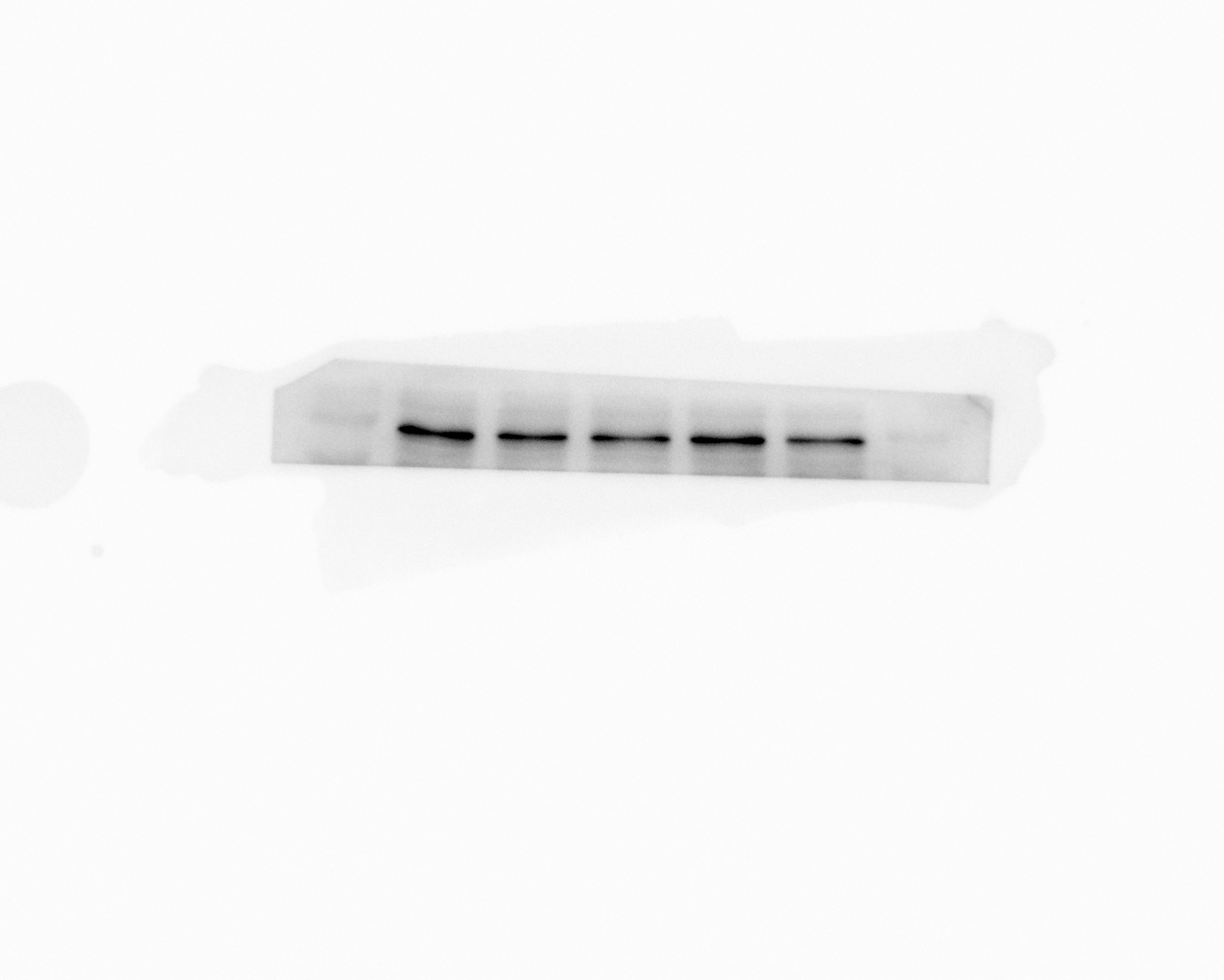

Supplement: S3 Appendix — (ZIP) [file pone.0266144.s003.zip › S3 Appendix/Western blot bands/WB LPS&P4 NFKB/P65__1_201209_153909_00.12.000_1_3281.jpg]

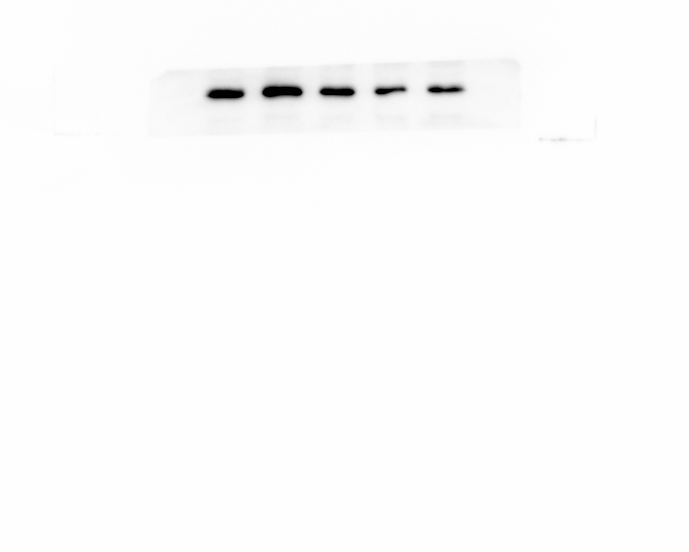

Supplement: S3 Appendix — (ZIP) [file pone.0266144.s003.zip › S3 Appendix/Western blot bands/WB LPS&P4 NFKB/PIKB_3_201231_132210_00.08.000_1_9795.tif]

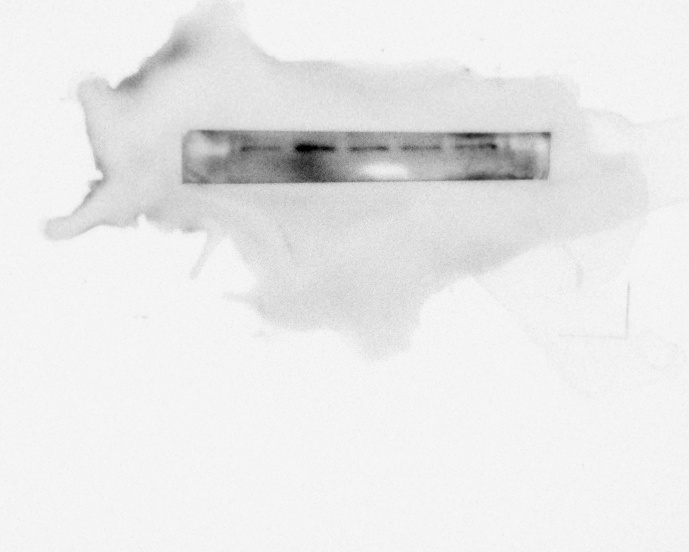

Supplement: S3 Appendix — (ZIP) [file pone.0266144.s003.zip › S3 Appendix/Western blot bands/WB LPS&P4 NFKB/PP65_1_201205_114935_00.09.000_1_1317.jpg]

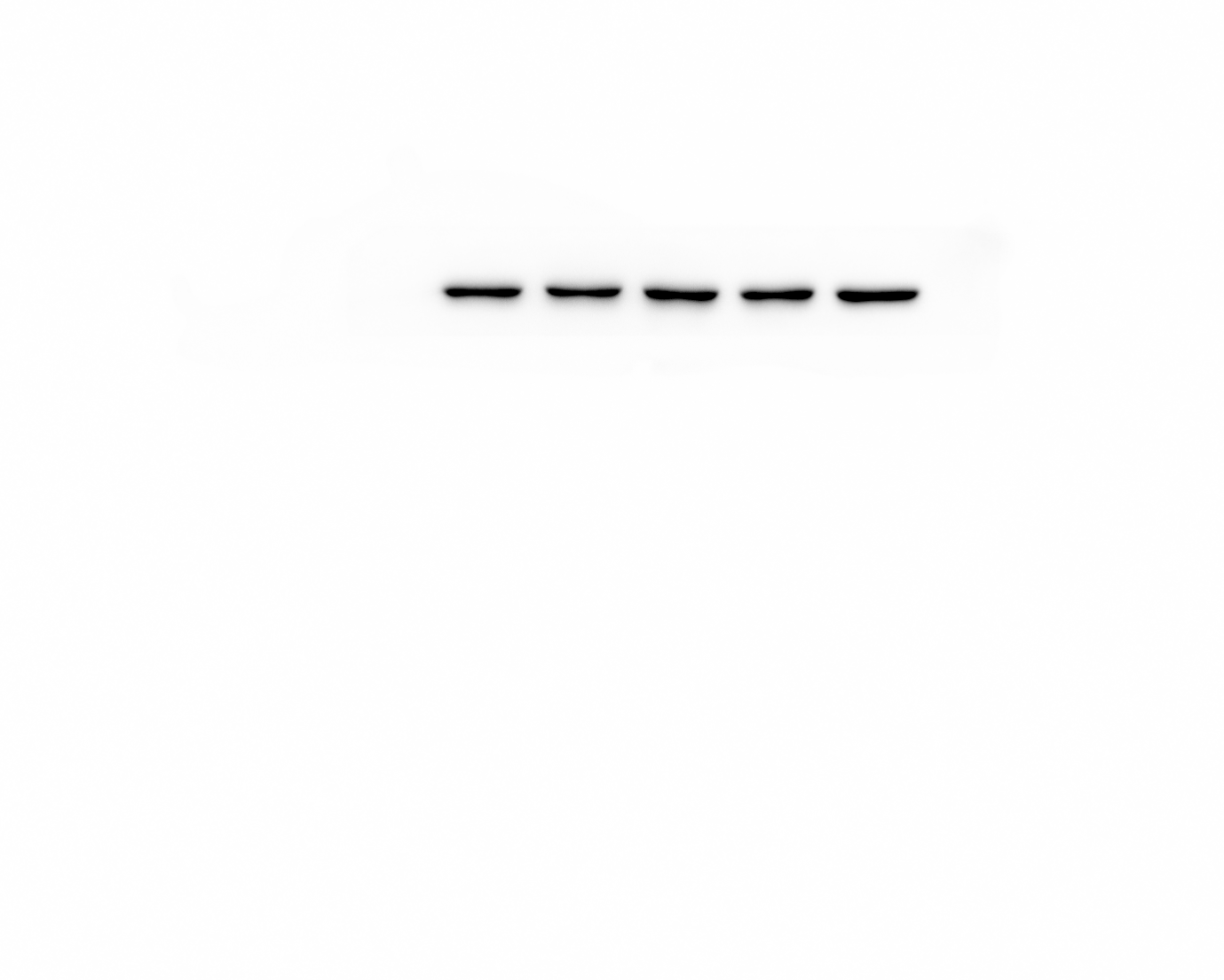

Supplement: S3 Appendix — (ZIP) [file pone.0266144.s003.zip › S3 Appendix/Western blot bands/WB LPS&P4-MAPK/BA_1_ERK-201223_102306_00.06.000_1_11927.tif]

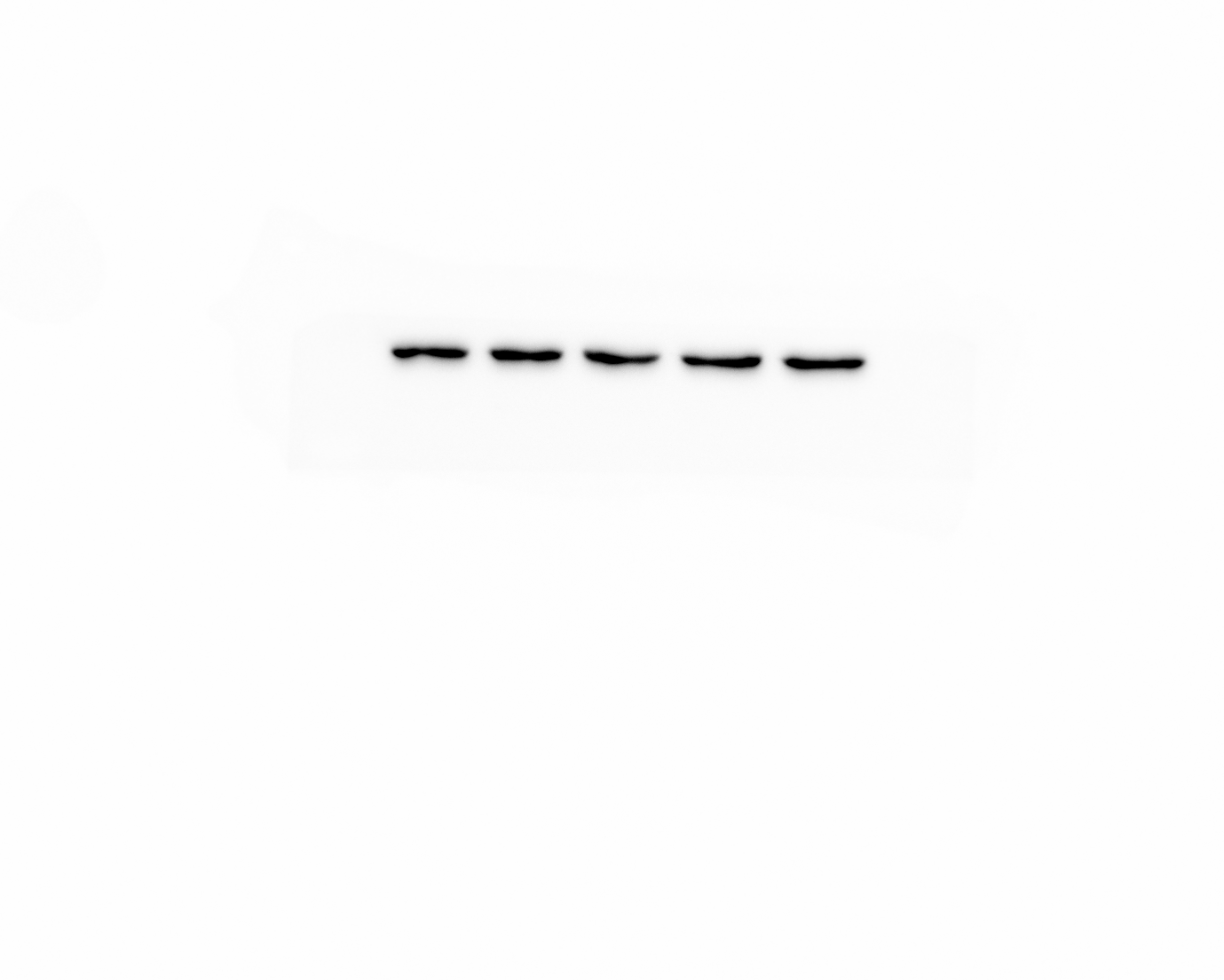

Supplement: S3 Appendix — (ZIP) [file pone.0266144.s003.zip › S3 Appendix/Western blot bands/WB LPS&P4-MAPK/BA_2_201226_132011_00.06.000_1_7865.tif]

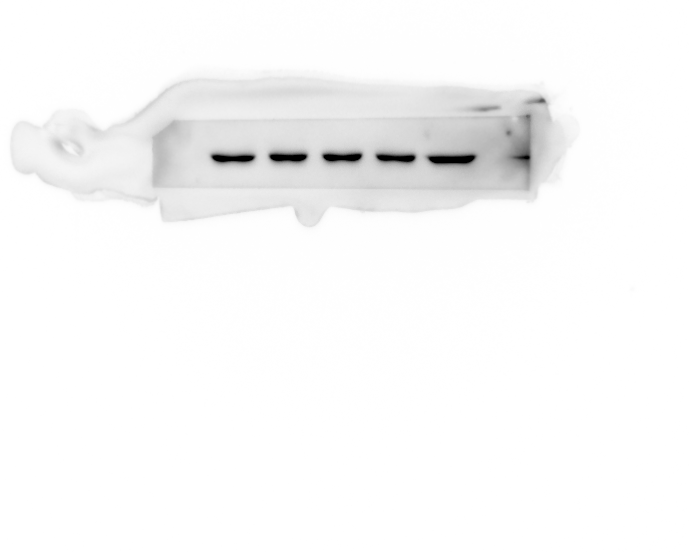

Supplement: S3 Appendix — (ZIP) [file pone.0266144.s003.zip › S3 Appendix/Western blot bands/WB LPS&P4-MAPK/BA_3_JNK-211115_140358_02.07.000_1_18720.tif]

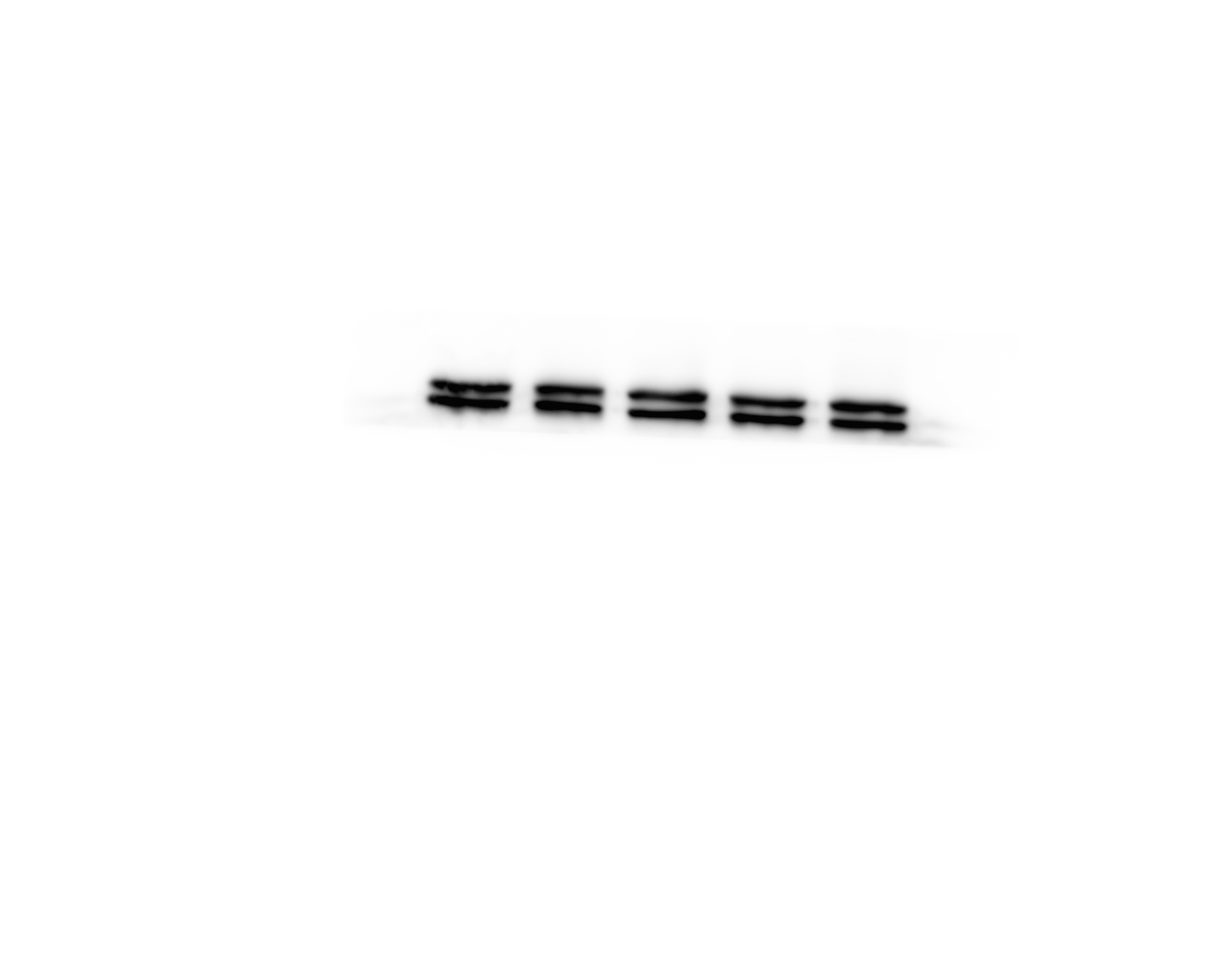

Supplement: S3 Appendix — (ZIP) [file pone.0266144.s003.zip › S3 Appendix/Western blot bands/WB LPS&P4-MAPK/ERK_1_201222_103426_00.03.000_3_203132.tif]

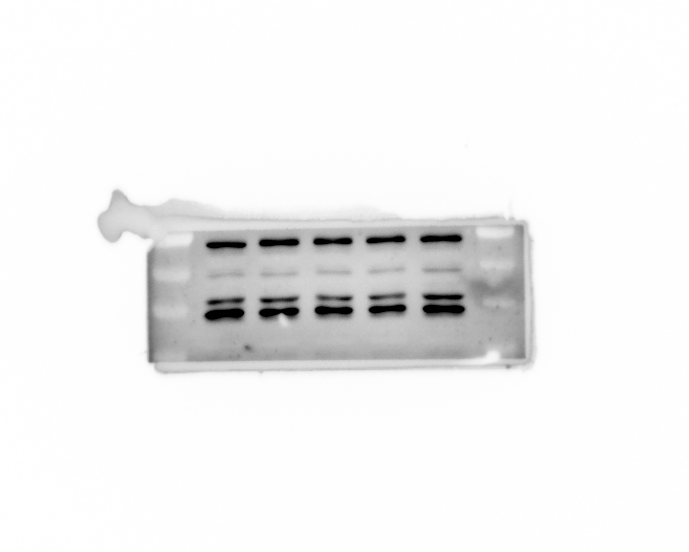

Supplement: S3 Appendix — (ZIP) [file pone.0266144.s003.zip › S3 Appendix/Western blot bands/WB LPS&P4-MAPK/JNK_1_211114_110013_02.35.000_1_13809.tif]

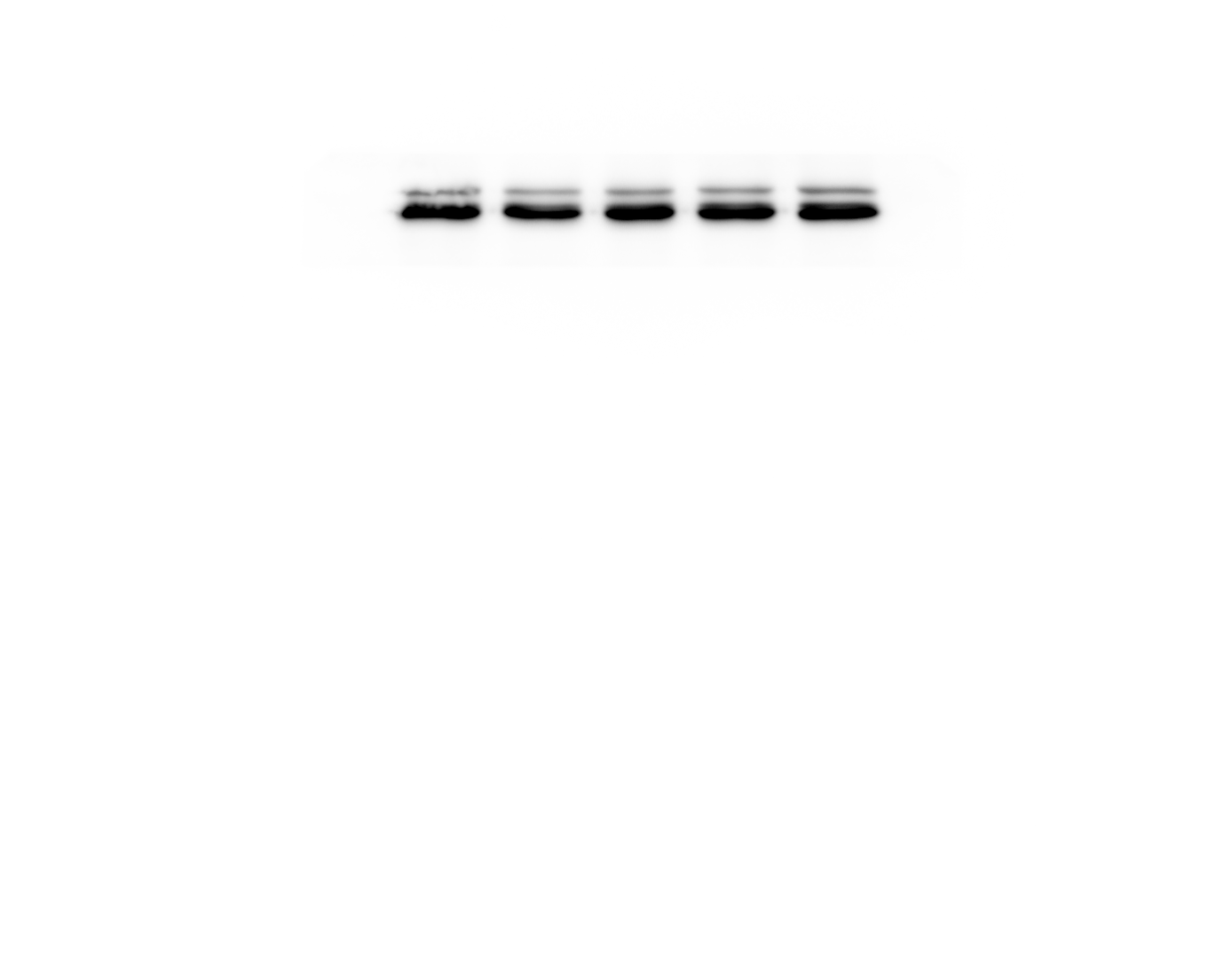

Supplement: S3 Appendix — (ZIP) [file pone.0266144.s003.zip › S3 Appendix/Western blot bands/WB LPS&P4-MAPK/P38_1_201223_102639_00.03.000_1_32788.tif]

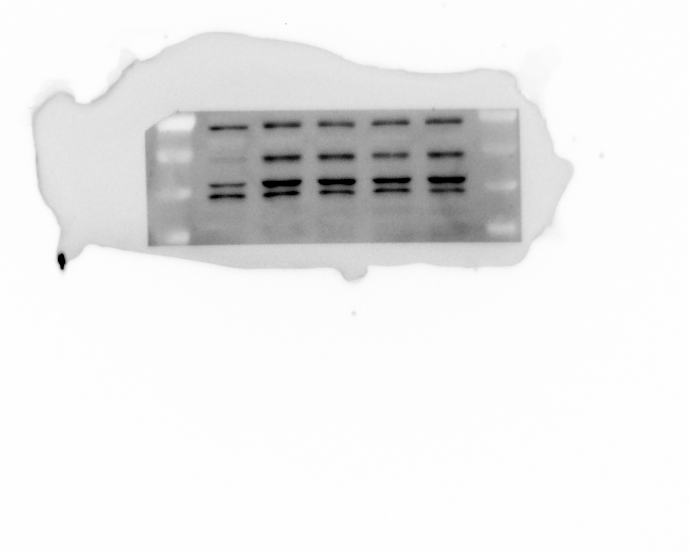

Supplement: S3 Appendix — (ZIP) [file pone.0266144.s003.zip › S3 Appendix/Western blot bands/WB LPS&P4-MAPK/PJNK_3_211114_111612_00.50.000_1_7718.tif]

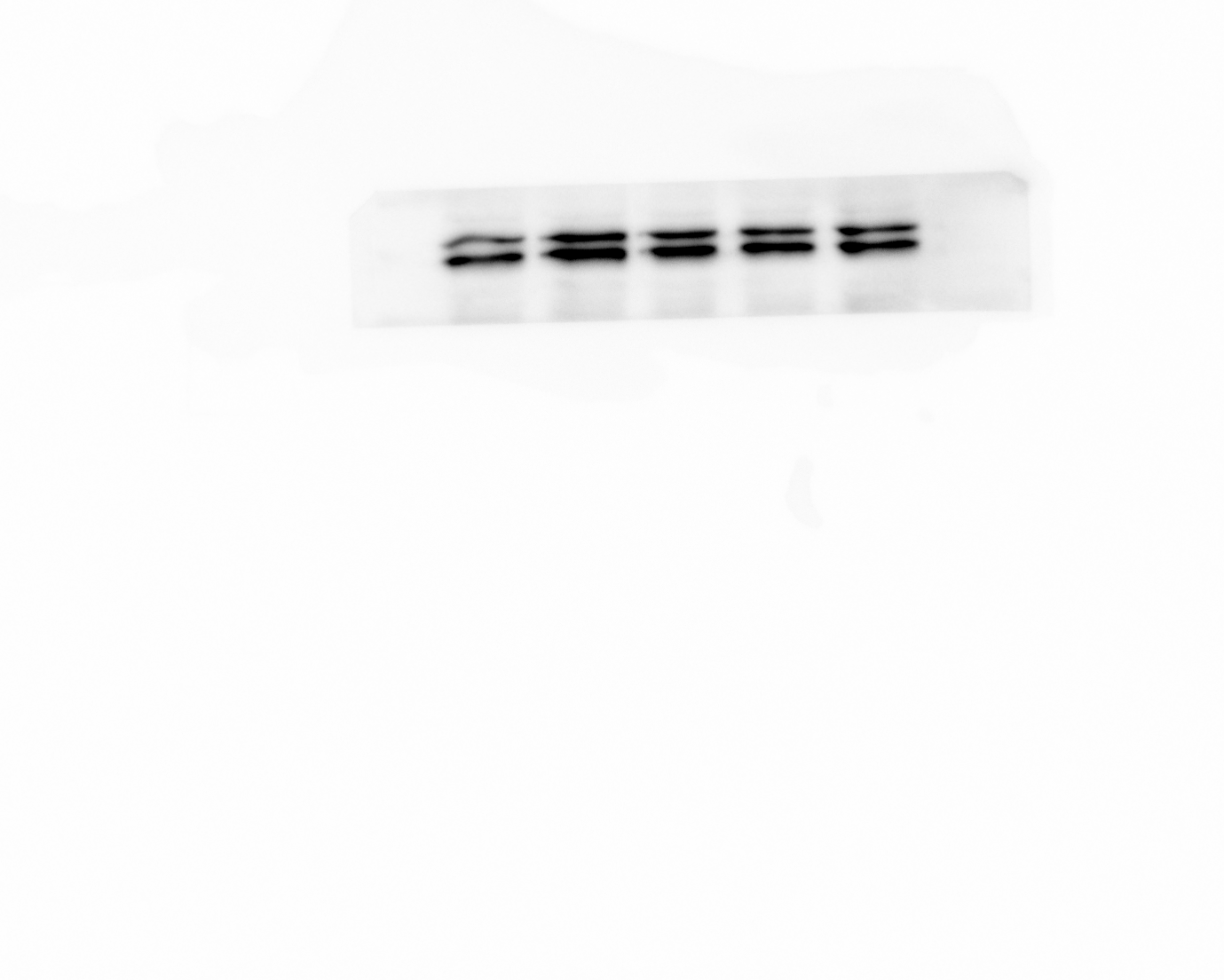

Supplement: S3 Appendix — (ZIP) [file pone.0266144.s003.zip › S3 Appendix/Western blot bands/WB LPS&P4-MAPK/PP38_2_201223_102924_00.07.000_1_6165.tif]

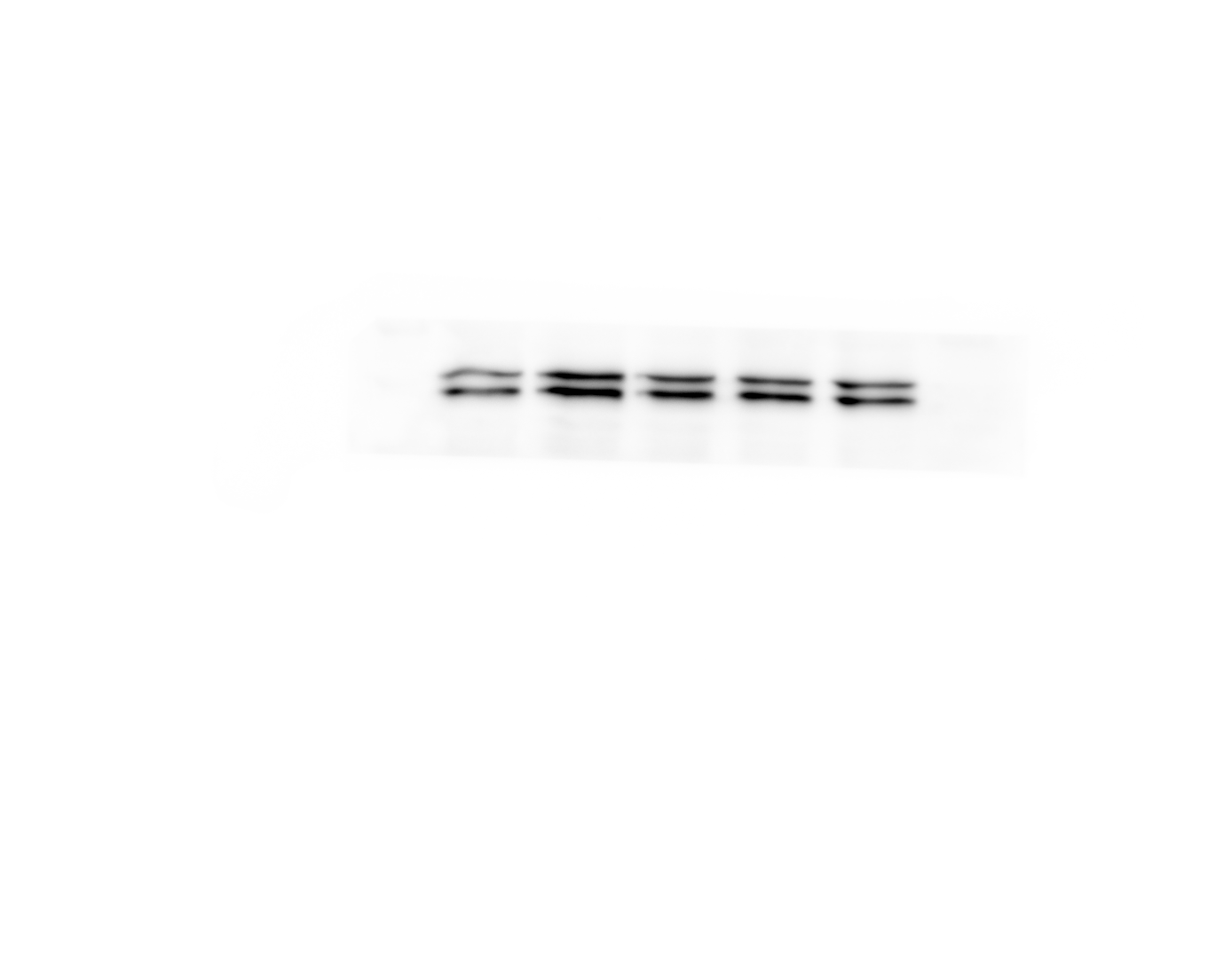

Supplement: S3 Appendix — (ZIP) [file pone.0266144.s003.zip › S3 Appendix/Western blot bands/WB LPS&P4-MAPK/P_ERK_2_201222_103143_00.08.000_1_35282.tif]

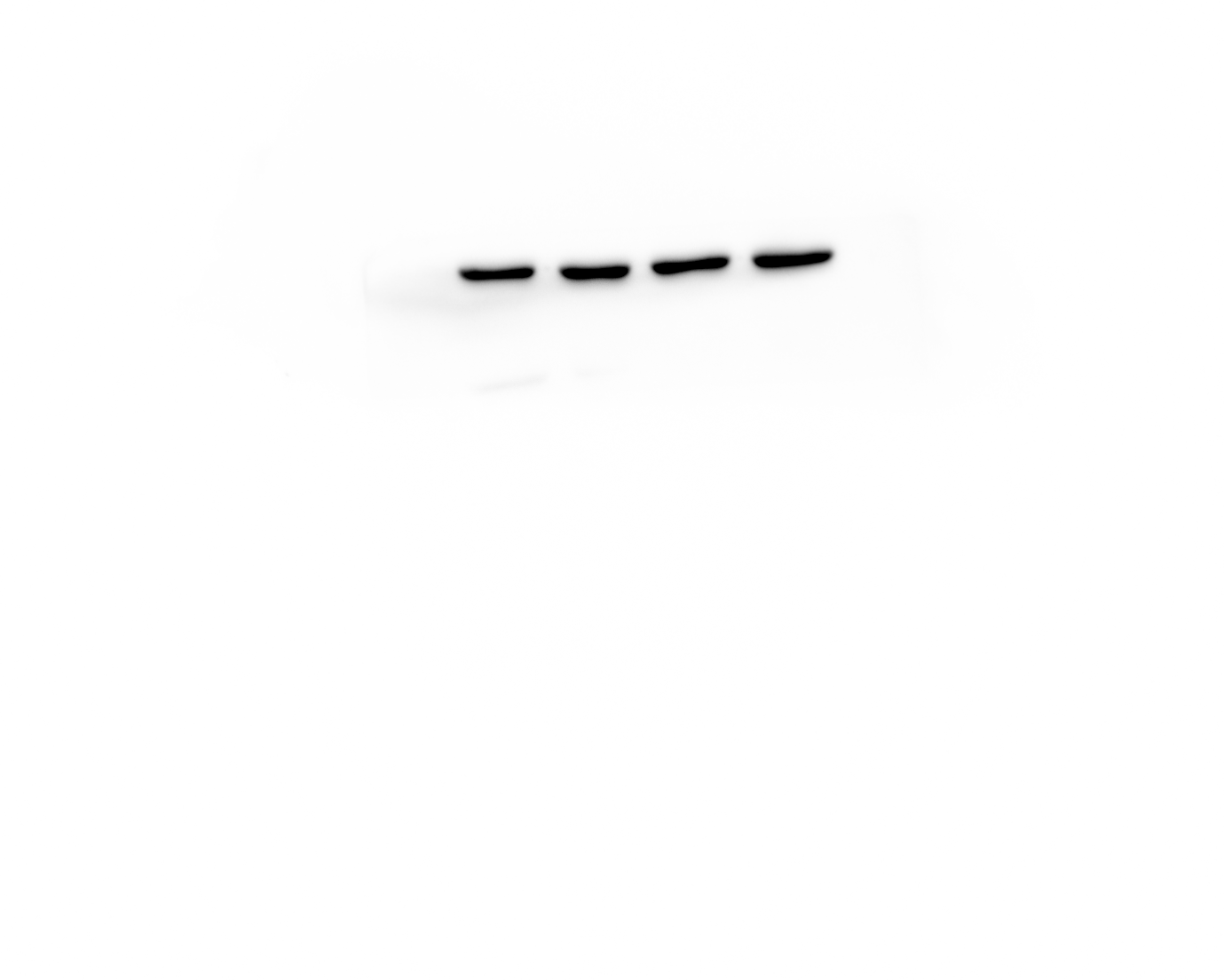

Supplement: S3 Appendix — (ZIP) [file pone.0266144.s003.zip › S3 Appendix/Western blot bands/WB P4-MAPK/BA_1_ERK-211123_124627_00.11.000_1_16190.tif]

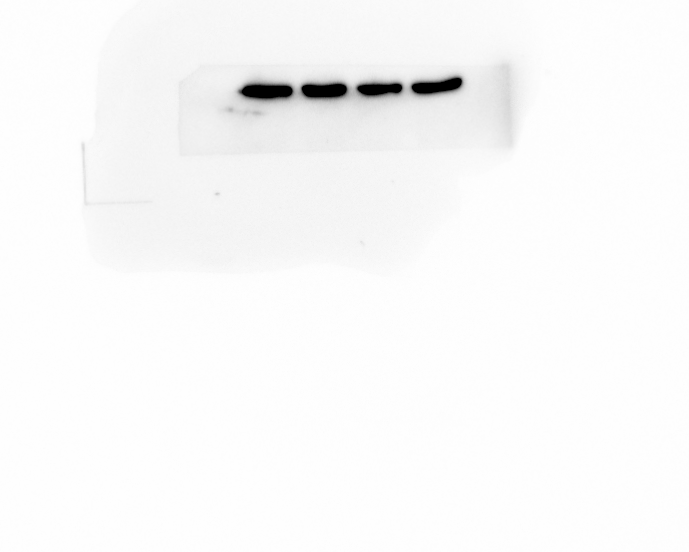

Supplement: S3 Appendix — (ZIP) [file pone.0266144.s003.zip › S3 Appendix/Western blot bands/WB P4-MAPK/BA_2_JNK-211126_132559_00.03.000_1_6422.tif]

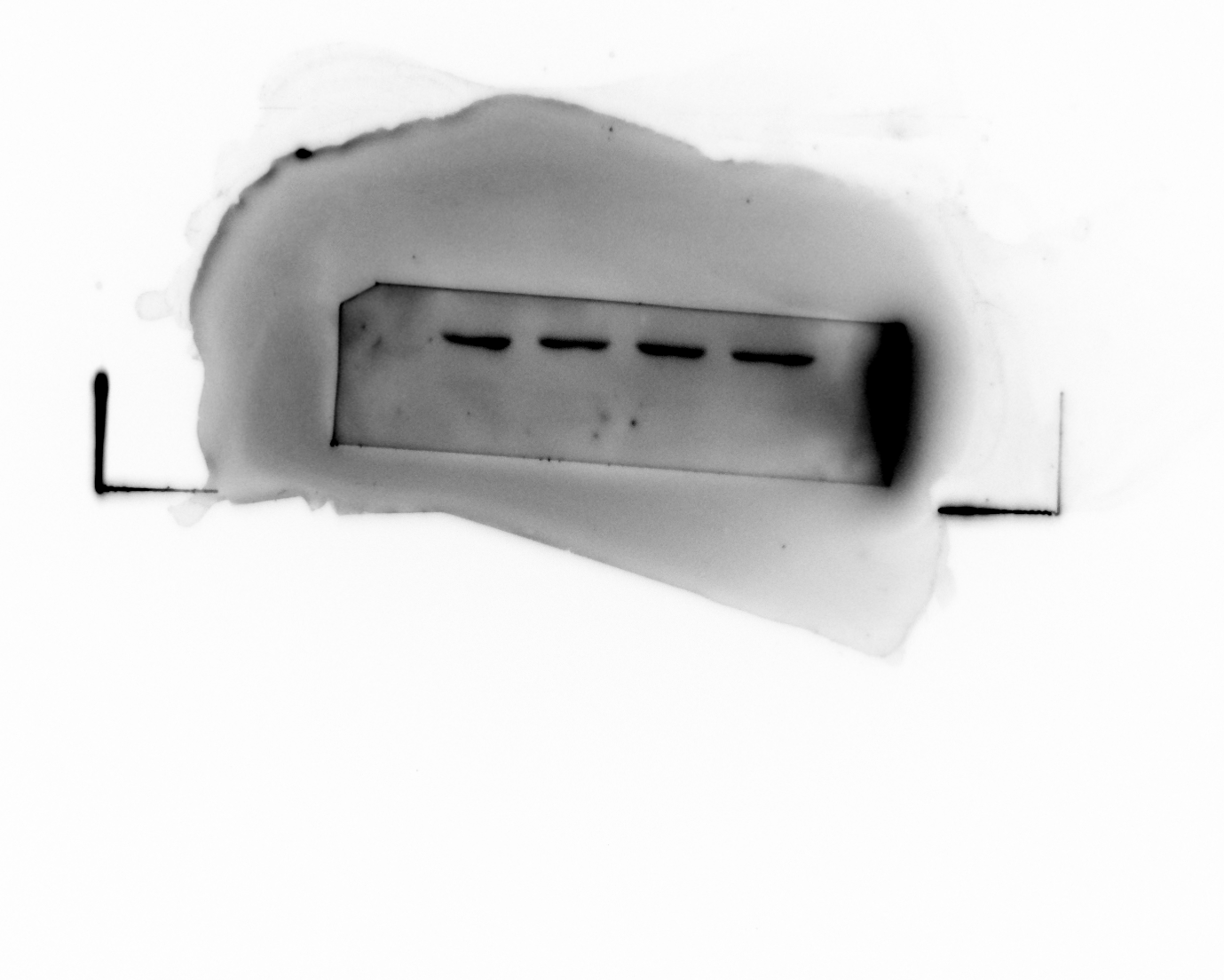

Supplement: S3 Appendix — (ZIP) [file pone.0266144.s003.zip › S3 Appendix/Western blot bands/WB P4-MAPK/BA_4_P38-211123_130330_06.39.000_1_5000.tif]

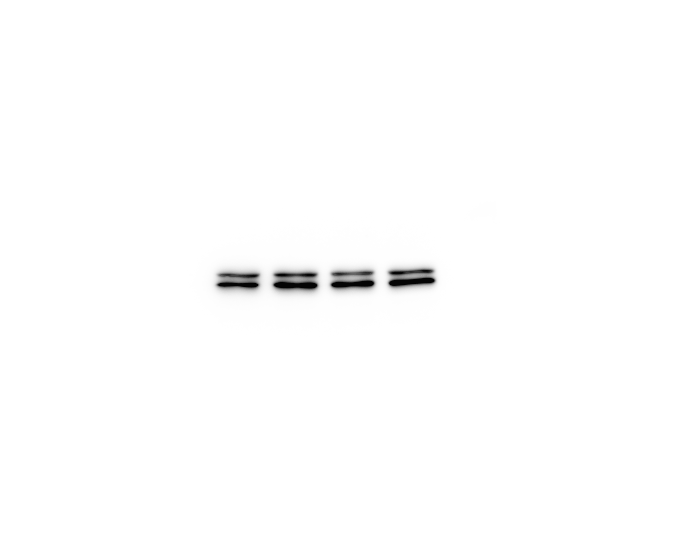

Supplement: S3 Appendix — (ZIP) [file pone.0266144.s003.zip › S3 Appendix/Western blot bands/WB P4-MAPK/ERK_1_211122_142021_00.01.000_3_131132.tif]

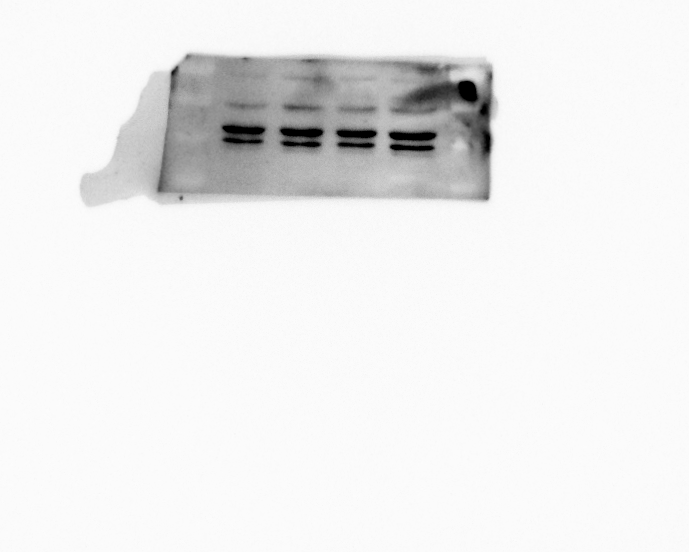

Supplement: S3 Appendix — (ZIP) [file pone.0266144.s003.zip › S3 Appendix/Western blot bands/WB P4-MAPK/JNK_1_211126_131437_00.28.000_1_3190.tif]

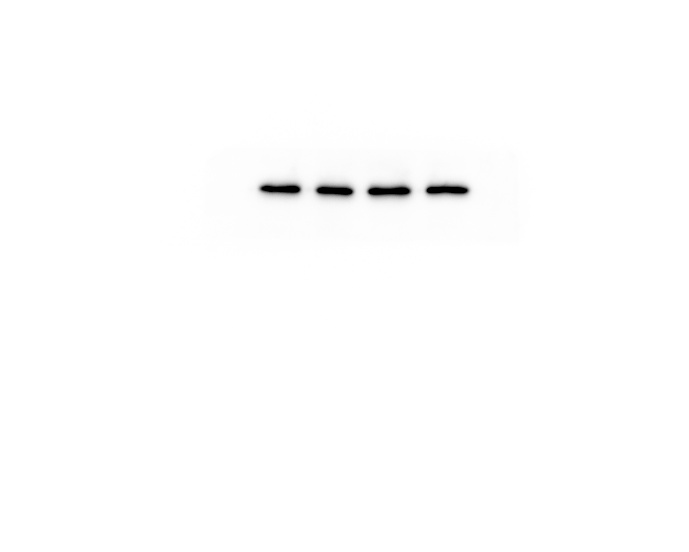

Supplement: S3 Appendix — (ZIP) [file pone.0266144.s003.zip › S3 Appendix/Western blot bands/WB P4-MAPK/P38_2_211124_114009_00.01.000_1_25576.tif]

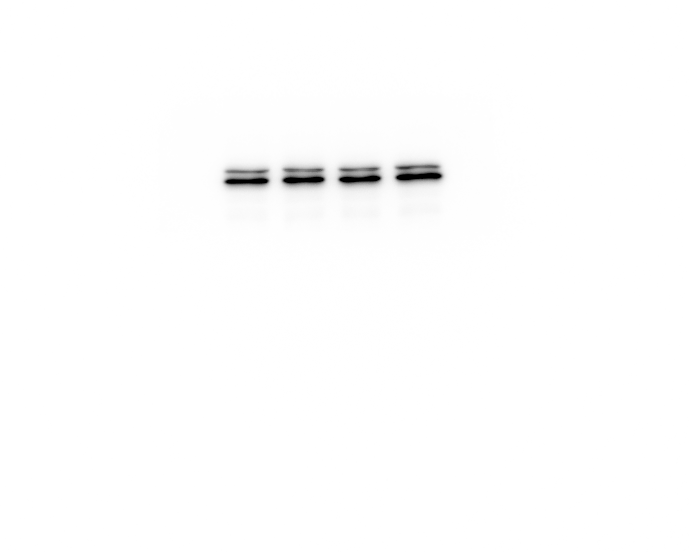

Supplement: S3 Appendix — (ZIP) [file pone.0266144.s003.zip › S3 Appendix/Western blot bands/WB P4-MAPK/PERK_2_211122_142437_00.01.000_1_20618.tif]

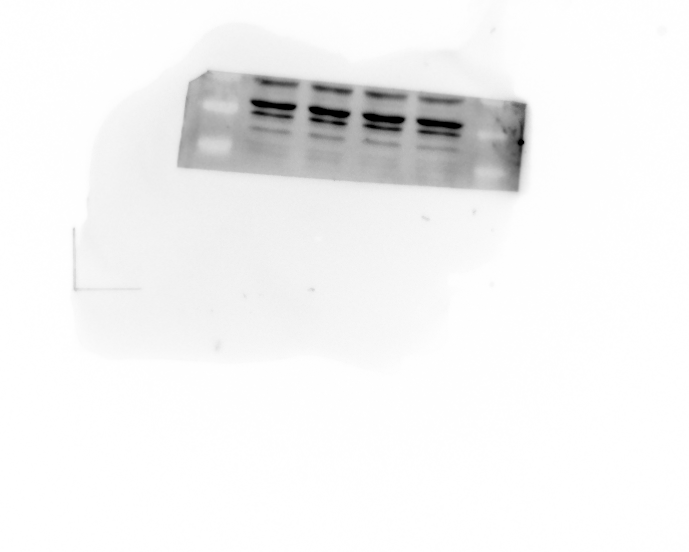

Supplement: S3 Appendix — (ZIP) [file pone.0266144.s003.zip › S3 Appendix/Western blot bands/WB P4-MAPK/PJNK_3_211126_132232_00.12.000_1_12498.tif]

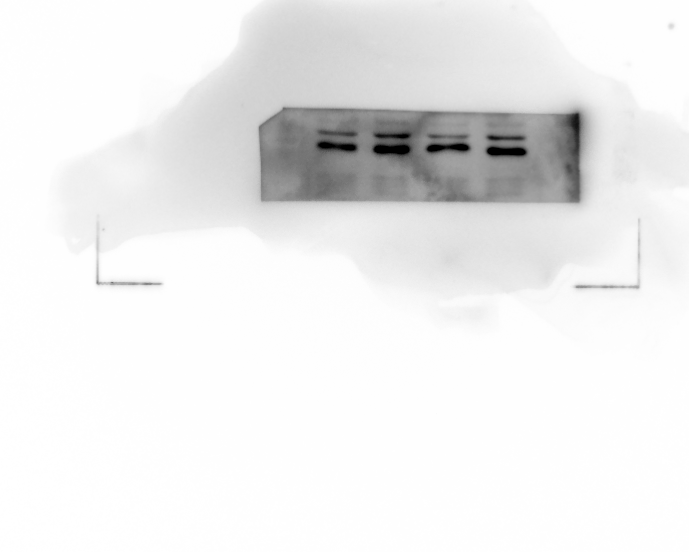

Supplement: S3 Appendix — (ZIP) [file pone.0266144.s003.zip › S3 Appendix/Western blot bands/WB P4-MAPK/PP38_2_211124_114842_00.43.000_1_16168.tif]

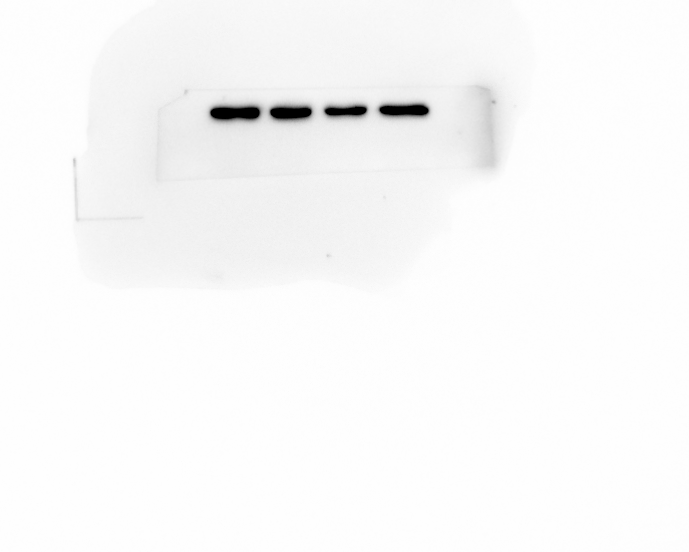

Supplement: S3 Appendix — (ZIP) [file pone.0266144.s003.zip › S3 Appendix/Western blot bands/WB P4-NFKB/BA_1_IKB-211126_132507_00.05.000_1_7296.tif]

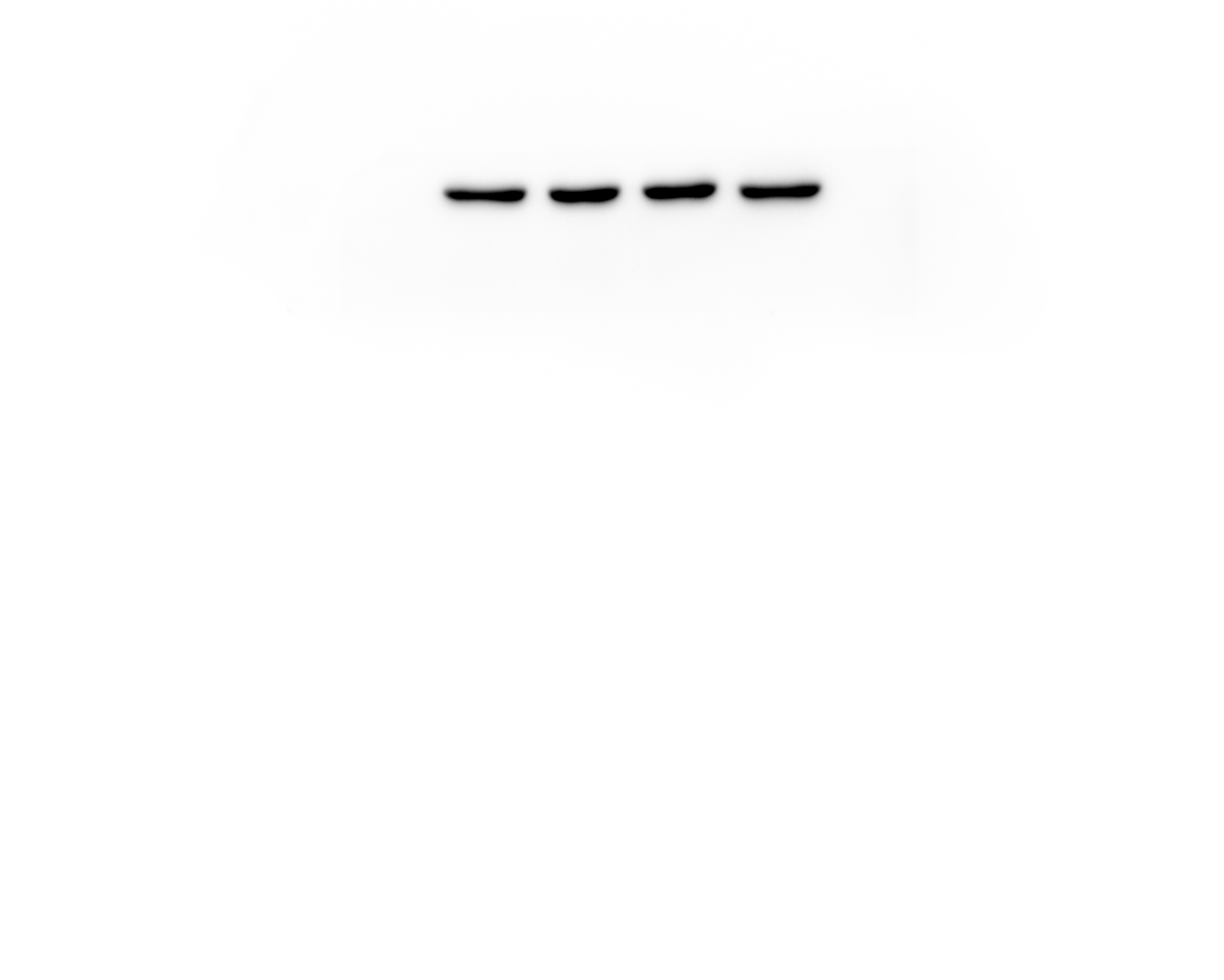

Supplement: S3 Appendix — (ZIP) [file pone.0266144.s003.zip › S3 Appendix/Western blot bands/WB P4-NFKB/BA_2_P65-211123_124730_00.15.000_1_15740.tif]

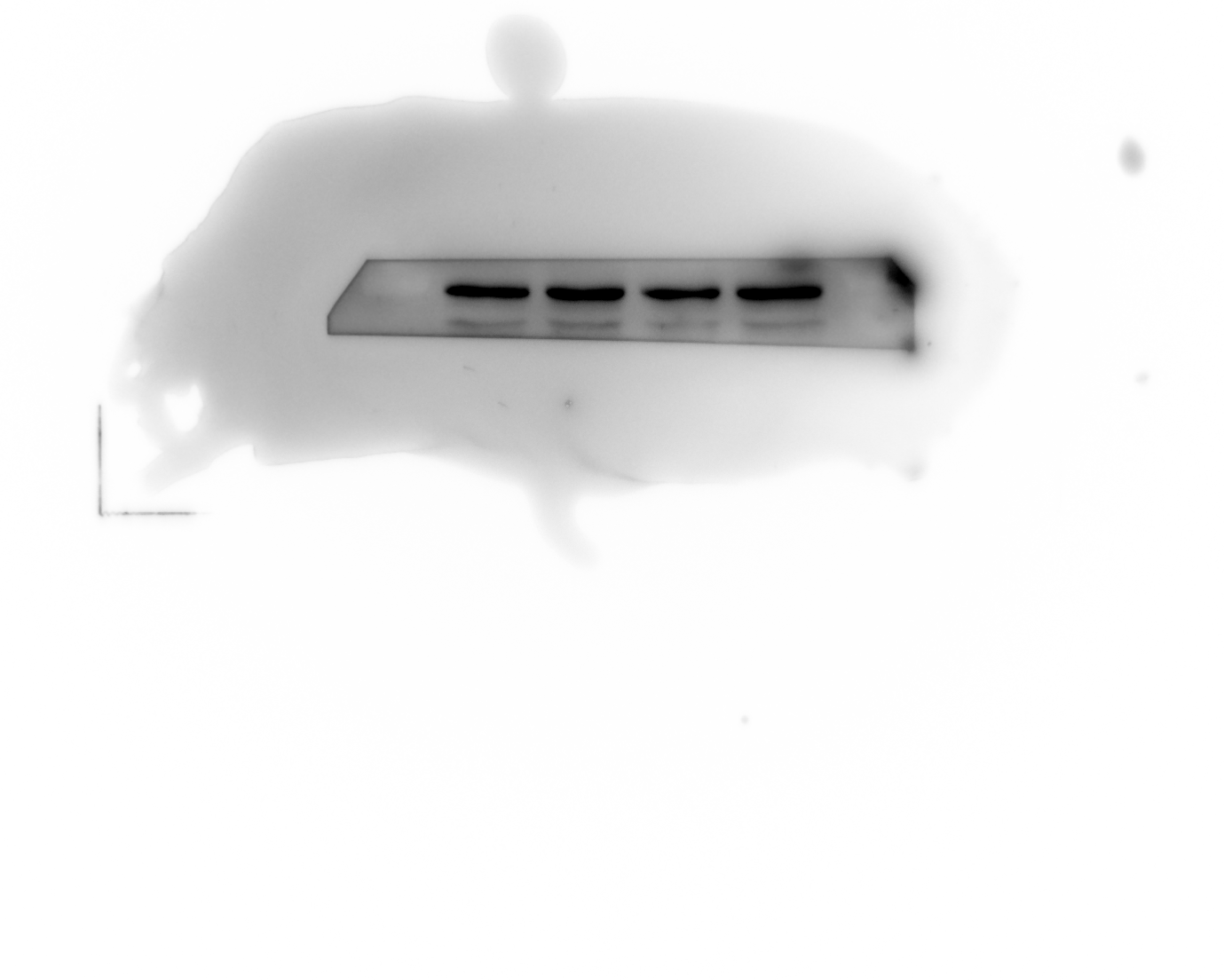

Supplement: S3 Appendix — (ZIP) [file pone.0266144.s003.zip › S3 Appendix/Western blot bands/WB P4-NFKB/IKB_2_211125_153246_00.26.000_1_17132.tif]

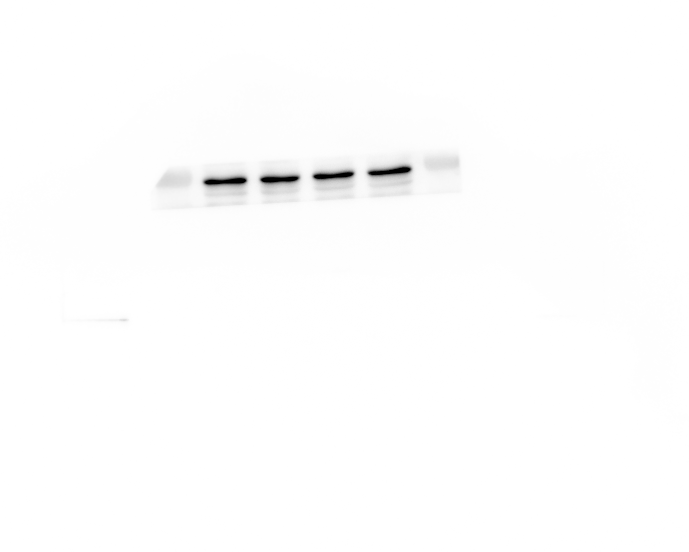

Supplement: S3 Appendix — (ZIP) [file pone.0266144.s003.zip › S3 Appendix/Western blot bands/WB P4-NFKB/P65_1_211122_143806_00.02.000_1_20433.tif]

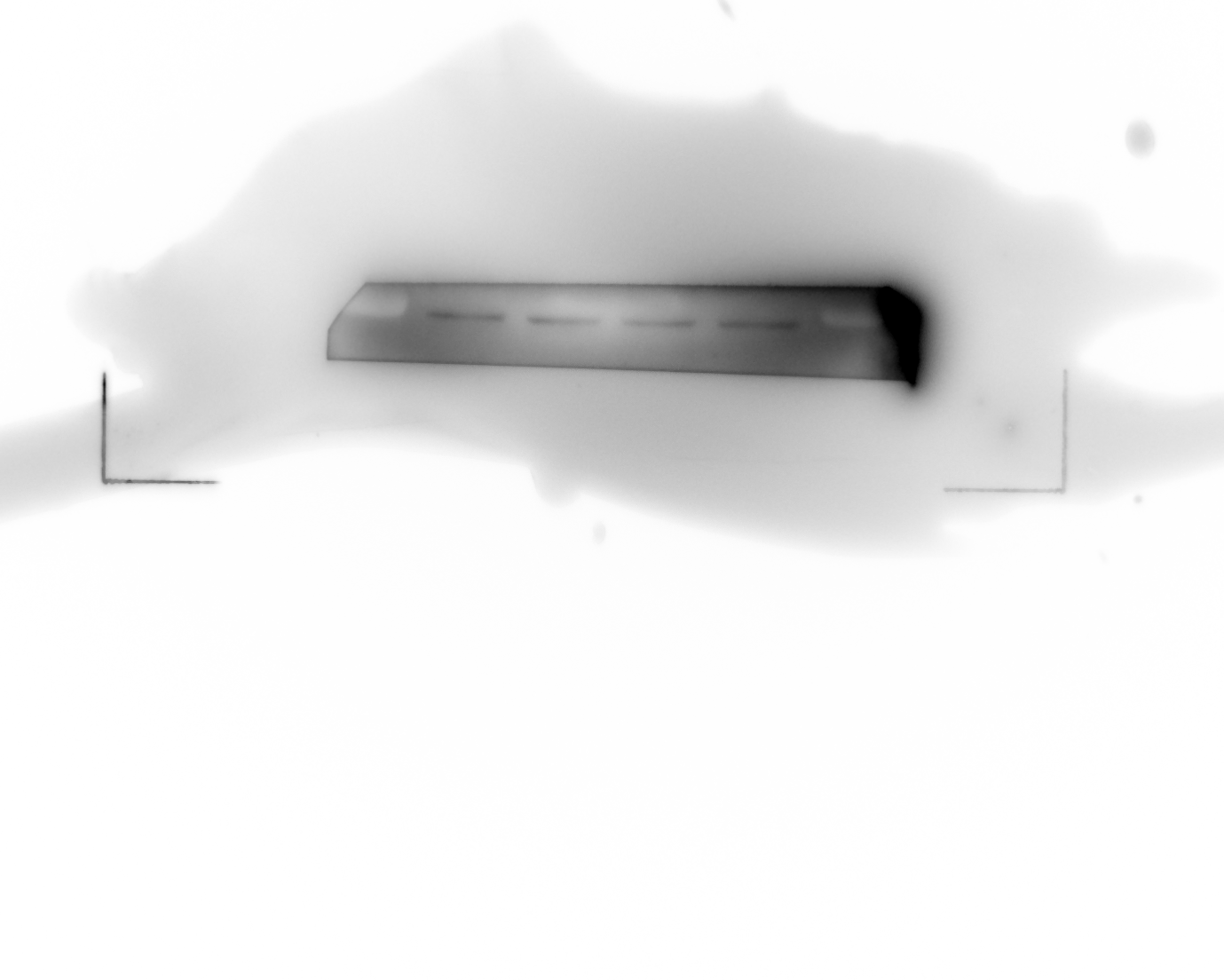

Supplement: S3 Appendix — (ZIP) [file pone.0266144.s003.zip › S3 Appendix/Western blot bands/WB P4-NFKB/PIKB_2_211125_153623_00.28.000_1_17200.tif]

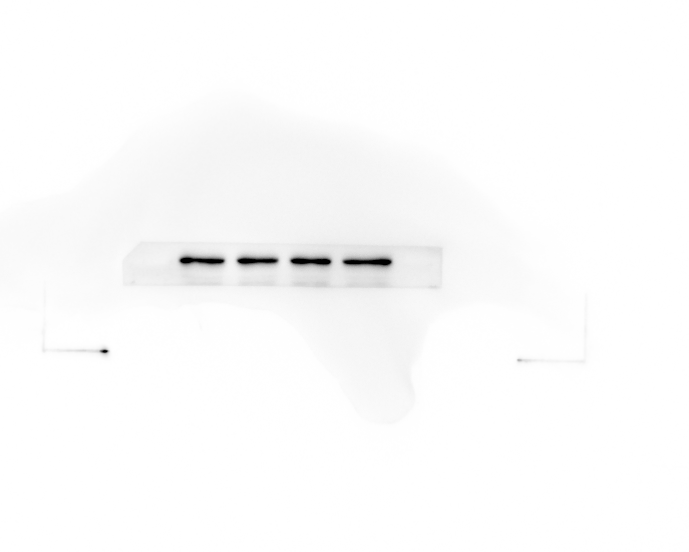

Supplement: S3 Appendix — (ZIP) [file pone.0266144.s003.zip › S3 Appendix/Western blot bands/WB P4-NFKB/PP65_2_211122_144137_00.11.000_2_16348.tif]
